# Supplementary material for: Increased DNA methylation variability in rheumatoid arthritis-discordant monozygotic twins
Source: Genome Med. 2018 Sep 4;10:64. doi: 10.1186/s13073-018-0575-9 (PMC6122744; doi:10.1186/s13073-018-0575-9)
Supplement: Supplementary file 1 — Figure S1. Multidimensional scaling plot of DMARD use in RA-discordant twins. Figure S2. Cell composition estimates for RA-discordant twins. Figure S3. Feature enrichment for differentially variable positions. Figure S4. Feature enrichment for DVPs identified in both RA and type 1 diabetes disease-discordant twins. Figure S5. Variance and range for DVPs which were hypervariable in healthy co-twins. (PDF 423 KB) (PDF 410 kb) [file 13073_2018_575_MOESM1_ESM.pdf]

Additional File 1

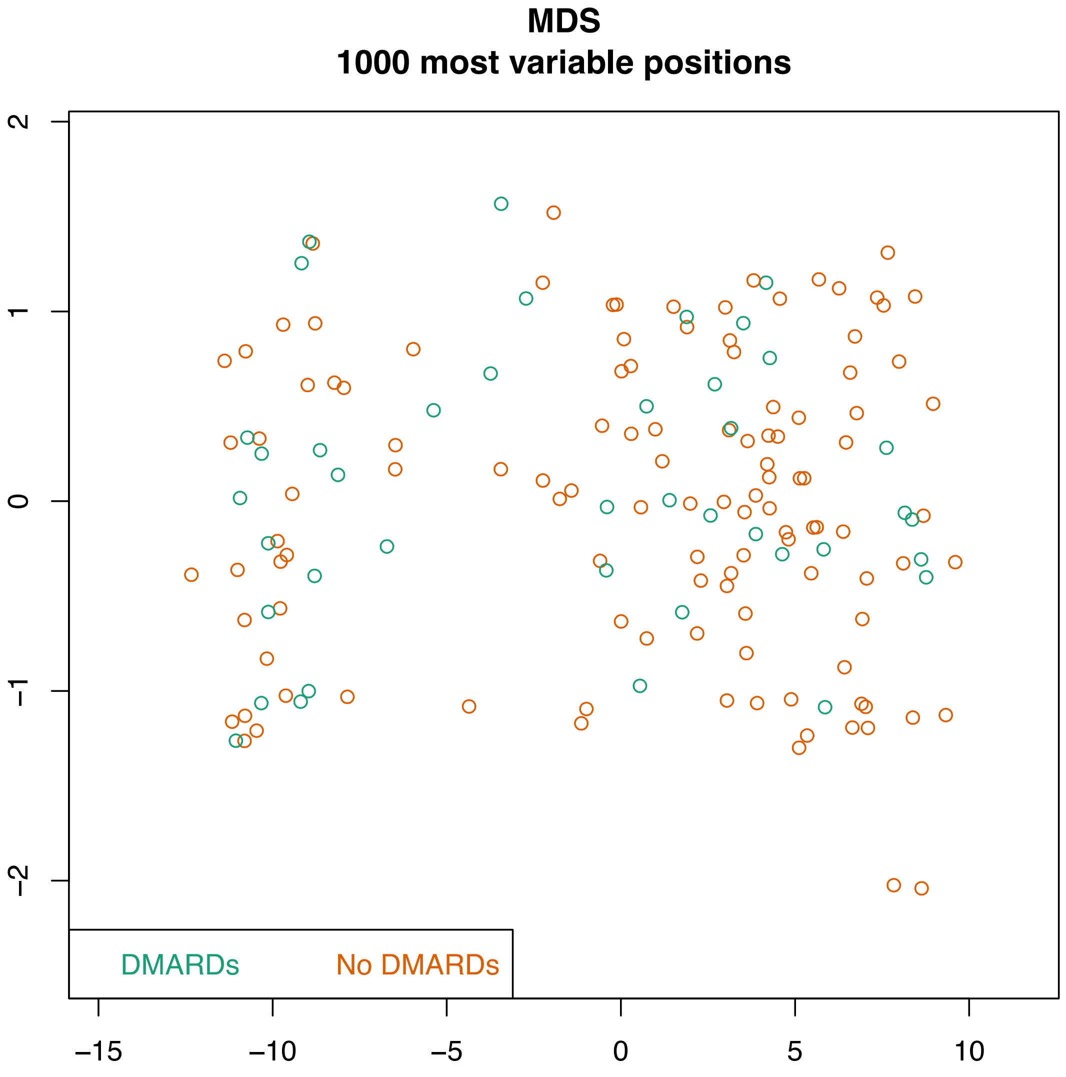

Figure S1. Multidimensional scaling plot of DMARD use in RA discordant twins. MDS plot showing similarities and differences between samples using Euclidian distances based on methylation values of the 1000 most variable positions in analysis. The samples are coloured by DMARD use and the treatment does not differentiate samples into discrete groups.

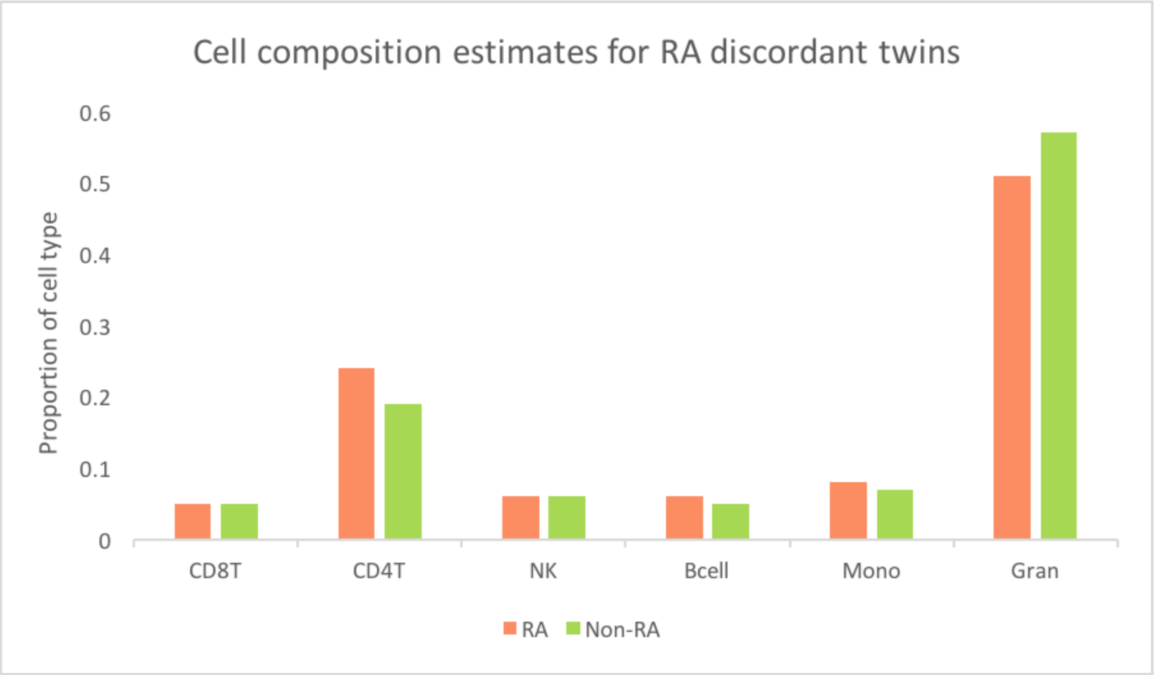

Figure S2: Cell composition estimates for RA discordant twins. Plot shows relative proportions of each cell type for RA and non-RA co-twins, estimated from DNA methylation data using bioinformatics methods. Proportions of each cell type were not found to be statistically different between the two comparison groups (p=0.05).

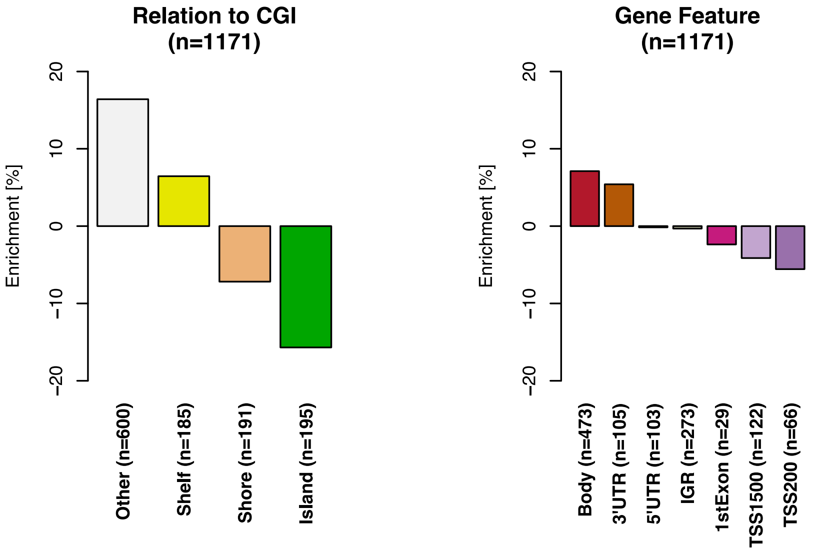

Figure S3: Feature enrichment for differentially variable positions. Differentially variable positions are primarily enriched in regions not associated with CpG Islands. Gene feature enrichment showed that differentially variable positions were enriched in gene bodies and 3'UTR regions of genes.

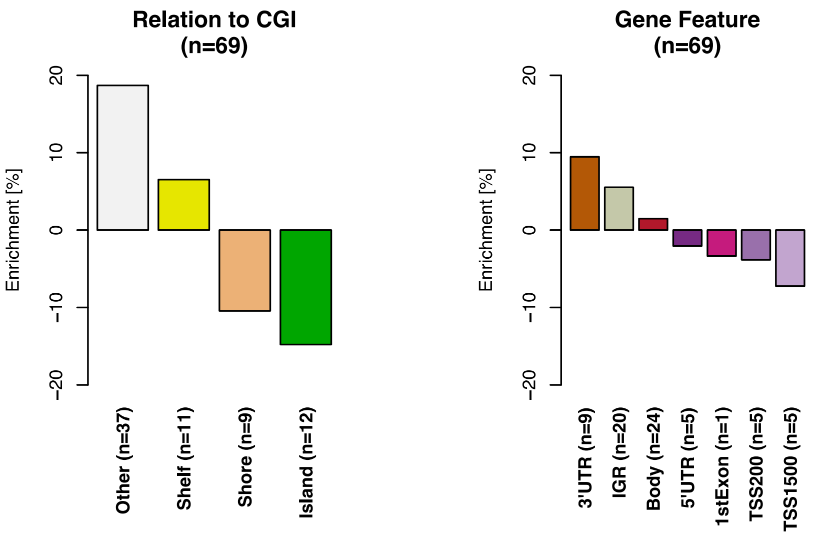

Figure S4: Feature enrichment for DVPs identified in both RA and type 1 diabetes disease-discordant twins. DVPs were not enriched in CpG Island associated features (left), but were enriched in 3'UTR sites of genes.

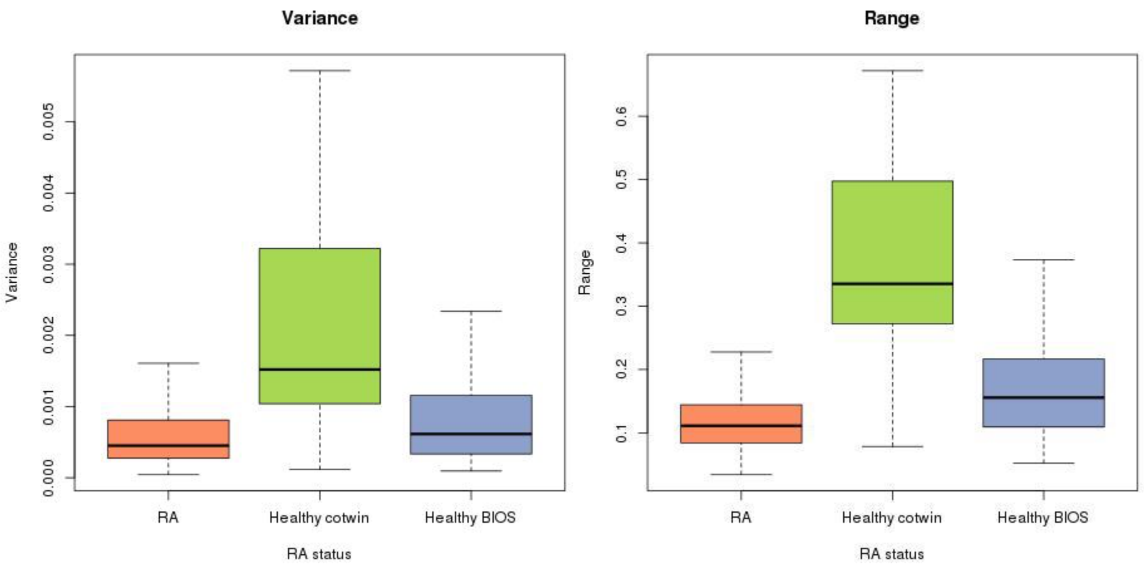

Figure S5: Variance and range for DVPs which were hypervariable in healthy co-twins. Variance and range were calculated for each of the 408 DVPs found to be hypervariable in healthy co-twins, plotted for three comparison groups; RA co-twins, healthy co-twins and an independent cohort of healthy individuals.
