# Supplementary material for: Increased DNA methylation variability in rheumatoid arthritis-discordant monozygotic twins
Source: Genome Med. 2018 Sep 4;10:64. doi: 10.1186/s13073-018-0575-9 (PMC6122744; doi:10.1186/s13073-018-0575-9)
Supplement: Supplementary file 2 — Table S1. Full list of differentially variable positions (n = 1171) between RA-affected and non-RA twins. Probe names are shown, along with t-statistic p value, Bartlett’s test for differential variability, which group was hypervariable, and probe annotation. (PDF 340 KB) (PDF 334 kb) [file 13073_2018_575_MOESM2_ESM.pdf]

Additional file 2: Table S1

Full list of differentially variable positions (n=1171) between RA affected and non-RA twins.

	Name	t	P(TT)	Av1	Av0	log[V1/V0]	P(BT)	q(BT)	Relation_to_	UCSC_RefGe	UCSC_RefGene_N	CHR
1	cg11374732	3.56602	0.00051333	0.89584	0.88816	-1.54461	4.09E-06	6.19E-05	Island	Body;Body	INPP5D;INPP5D	2
2	cg01999539	-3.51217	0.00061405	0.03237	0.038262	-1.44742	1.50E-05	0.0002014	Island			6
3	cg23280983	-3.47767	0.00068981	0.04074	0.044641	-1.42111	2.11E-05	0.0002735	Island	TSS200	C2orf42	2
4	cg20500144	-3.38125	0.00095024	0.03444	0.038065	-1.32762	6.82E-05	0.0007821	S_Shore	Body	ARL6IP5	3
5	cg26985354	3.37305	0.00100839	0.82677	0.808428	-1.89676	2.18E-08	5.06E-07	Island	Body	SFRS16	19
6	cg26827503	3.35759	0.0010324	0.90709	0.895271	-1.40244	2.68E-05	0.0003391		Body	TMEM156	4
7	cg11913894	-3.35416	0.00105369	0.05391	0.061551	-1.56879	2.93E-06	4.57E-05	Island	TSS1500;TSS	MAP3K4;MAP3K4	6
8	cg16583193	3.33378	0.00112527	0.90112	0.890407	-1.53777	4.49E-06	6.74E-05	Island	Body	PIP5K1C	19
9	cg15573998	3.32753	0.00113928	0.90809	0.895486	-1.38096	3.52E-05	0.0004332		Body;Body	NRXN1;NRXN1	2
10	cg16012388	3.31749	0.00118652	0.88389	0.858575	-1.52485	5.35E-06	7.91E-05	S_Shelf	Body	BICC1	10
11	cg16397722	3.31583	0.00119032	0.9106	0.902311	-1.48211	9.50E-06	0.0001332		Body;Body;E	TP53;TP53;TP53;T	17
12	cg20556304	3.32819	0.00119258	0.89495	0.883802	-2.2681	3.94E-11	1.44E-09				6
13	cg25173129	-3.28306	0.00134302	0.84275	0.859246	1.738848	2.51E-07	4.82E-06		TSS1500	EPX	17
14	cg01928104	3.27086	0.00138494	0.93037	0.919654	-1.56463	3.10E-06	4.81E-05		TSS1500	SNORD114-7	14
15	cg06734169	-3.24141	0.00153437	0.04848	0.057122	-1.70436	4.20E-07	7.73E-06	N_Shore	5'UTR;5'UTR	LRRFIP2;LRRFIP2;	3
16	cg04845047	3.23024	0.00156418	0.84237	0.827504	-1.3548	4.88E-05	0.0005797				13
17	cg26788916	-3.18744	0.00179286	0.04936	0.054967	-1.3218	7.32E-05	0.0008329	Island			20
18	cg00340024	3.17676	0.001861	0.92012	0.913932	-1.39327	3.01E-05	0.0003767				1
19	cg08306614	3.15804	0.00198051	0.88541	0.875822	-1.45147	1.42E-05	0.0001918		3'UTR	NUDCD3	7
20	cg19929189	-3.16593	0.00198104	0.0504	0.057913	-2.00753	3.59E-09	9.55E-08	N_Shore			6
21	cg00736681	-3.14122	0.00209443	0.86243	0.880463	1.50988	6.55E-06	9.51E-05		5'UTR;5'UTR	ST3GAL1;ST3GAL1	8
22	cg10112003	-3.13165	0.0021554	0.05317	0.059419	-1.47367	1.06E-05	0.0001472	Island	TSS200;TSS2	ACTL6A;ACTL6A;A	3
23	cg21136491	-3.12194	0.00221484	0.07529	0.082542	-1.39075	3.11E-05	0.0003878	Island	1stExon;TSS	CCT6A;PSPH;CCT6	7
24	cg24367761	3.13045	0.00221513	0.89302	0.871317	-2.0092	3.50E-09	9.31E-08		Body;3'UTR	MED23;MED23	6
25	cg03931660	3.0854	0.0024828	0.9352	0.926742	-1.3744	3.82E-05	0.000466	N_Shore	Body	ZNF423	16
26	cg17080021	3.07586	0.00259111	0.88718	0.876702	-1.69278	4.98E-07	9.04E-06		Body	NBPF7	1

27	cg24778130	-3.05766	0.00274656	0.04754	0.053246	-1.73667	2.60E-07	4.97E-06	Island	Body	CAMTA1	1
28	cg12934016	3.04869	0.00288335	0.87779	0.856664	-2.25706	4.81E-11	1.73E-09		5'UTR;TSS15	UBE3A;UBE3A	15
29	cg10597545	-3.03329	0.00291821	0.04006	0.04457	-1.35937	4.61E-05	0.0005508	Island	5'UTR;TSS20	HNRNPL;HNRNPL	19
30	cg19682395	3.03291	0.0029408	0.91028	0.897466	-1.53182	4.87E-06	7.26E-05		Body	EIF2AK4	15
31	cg25801052	3.031	0.00298365	0.87438	0.863395	-1.75078	2.10E-07	4.09E-06	Island	Body	ARID5A	2
32	cg01091261	3.03018	0.00302578	0.8402	0.821809	-2.04133	2.04E-09	5.66E-08	Island	Body	ADCY9	16
33	cg17839216	3.02093	0.0030503	0.7901	0.772872	-1.52233	5.54E-06	8.16E-05				5
34	cg23681311	-3.01634	0.00308733	0.04324	0.049487	-1.46767	1.15E-05	0.0001582	Island	1stExon;1stE	MAPK1;MAPK1;M	22
35	cg17032848	-2.99309	0.00333373	0.85709	0.86781	1.615281	1.52E-06	2.51E-05	S_Shelf	Body;Body;E	LRRFIP1;LRRFIP1;	2
36	cg09273012	2.98812	0.00335649	0.88386	0.861651	-1.38588	3.31E-05	0.00041		Body	SRBD1	2
37	cg14011077	-2.98646	0.00339101	0.88002	0.899148	1.528648	5.08E-06	7.55E-05				9
38	cg06897306	2.97698	0.00352761	0.8891	0.881419	-1.81609	7.75E-08	1.64E-06	N_Shelf	Body;Body;E	PTPRN2;PTPRN2;I	7
39	cg15024975	-2.96235	0.00363545	0.04181	0.04614	-1.42196	2.09E-05	0.000271	Island	1stExon;5'U	MDC1;MDC1	6
40	cg24855292	2.95807	0.00368448	0.88965	0.881358	-1.43352	1.80E-05	0.0002369		5'UTR	PARP4	13
41	cg16210497	-2.94635	0.00380231	0.07881	0.087691	-1.31336	8.11E-05	0.0009122	S_Shore	TSS1500	TBX18	6
42	cg21199045	-2.94694	0.00381485	0.8101	0.824861	1.463638	1.21E-05	0.000166	S_Shore			18
43	cg14154547	-2.95084	0.00394158	0.83363	0.851527	2.744734	4.29E-15	2.86E-13		Body	RXRA	9
44	cg14060719	2.92119	0.00411137	0.89801	0.884496	-1.37883	3.62E-05	0.0004436		Body	ATG2B	14
45	cg15292101	-2.9116	0.0042969	0.04919	0.056805	-1.83229	6.03E-08	1.30E-06	Island	TSS1500	CCDC136	7
46	cg02328221	-2.90515	0.00432812	0.78551	0.79901	1.477348	1.01E-05	0.000141	N_Shore	Body	GIPR	19
47	cg08497311	-2.91593	0.00433734	0.8613	0.873608	2.492731	6.08E-13	2.94E-11	S_Shelf	Body	FAM38A	16
48	cg22221575	-2.90674	0.00439113	0.78526	0.808496	2.043141	1.98E-09	5.50E-08		3'UTR	PCYT1A	3
49	cg22305516	-2.89667	0.00445087	0.84029	0.853883	1.556739	3.46E-06	5.32E-05		Body	SH3GL1	19
50	cg06009422	-2.89599	0.0044887	0.86197	0.873251	1.748741	2.17E-07	4.20E-06		5'UTR	AGPAT4	6
51	cg19747960	-2.88745	0.00454681	0.06188	0.068888	-1.36452	4.33E-05	0.00052		Body	C8orf12	8
52	cg04059146	2.88658	0.00455223	0.89305	0.883965	-1.31919	7.56E-05	0.0008567		TSS1500	MMP7	11
53	cg04773352	2.89216	0.00456194	0.88479	0.868759	-1.89147	2.37E-08	5.48E-07		Body	C2orf86	2
54	cg17444642	2.87267	0.00475723	0.90089	0.889342	-1.40379	2.64E-05	0.0003339		Body	EFNA5	5
55	cg25383057	-2.87261	0.00476346	0.78031	0.797584	1.44	1.65E-05	0.0002198	Island	Body	METRNL	17
56	cg10246038	2.87242	0.00484796	0.87537	0.853301	-1.96338	7.44E-09	1.87E-07		3'UTR;3'UTR	FAM82A1;FAM82	2

57	cg14063191	-2.86455	0.00488239	0.04955	0.055972	-1.46358	1.21E-05	0.0001661	Island	TSS1500	ZDHHC12	9
58	cg08270888	2.85798	0.00497023	0.91654	0.905548	-1.41052	2.42E-05	0.0003091				6
59	cg09820557	-2.86213	0.00498165	0.90014	0.909739	1.866922	3.50E-08	7.84E-07		3'UTR	ODF4	17
60	cg26023902	2.85741	0.00507526	0.88141	0.85998	-2.01343	3.26E-09	8.72E-08		TSS1500	LOC285735	6
61	cg05295028	2.84955	0.00509	0.89745	0.889329	-1.3732	3.88E-05	0.0004723		TSS1500	ZP1	11
62	cg09542250	2.84678	0.00515919	0.90559	0.899454	-1.54773	3.92E-06	5.95E-05	N_Shelf	Body	C8orf76	8
63	cg14975015	2.84354	0.00519849	0.81965	0.794124	-1.48412	9.25E-06	0.0001301		5'UTR;5'UTR	ATP6V0A4;ATP6V	7
64	cg19139727	2.84649	0.00521281	0.90765	0.89583	-1.84734	4.77E-08	1.04E-06				10
65	cg27529911	-2.82393	0.00553719	0.82223	0.834546	1.663121	7.67E-07	1.34E-05	S_Shelf			8
66	cg06754047	-2.81513	0.0056326	0.04114	0.047136	-1.37203	3.94E-05	0.0004785	N_Shore	TSS200	SMYD5	2
67	cg14582220	-2.81222	0.00571132	0.05637	0.062368	-1.55576	3.51E-06	5.38E-05	Island	TSS200	CLN6	15
68	cg09839874	2.81275	0.00573561	0.91145	0.903668	-1.74652	2.24E-07	4.33E-06	S_Shelf	Body;Body;B	CMTM1;CMTM1;	16
69	cg04126335	2.81744	0.00577059	0.92654	0.913163	-2.39844	3.61E-12	1.55E-10		Body	ITGA10	1
70	cg05184917	-2.80442	0.00583847	0.03831	0.04201	-1.52971	5.01E-06	7.45E-05	Island	Body	PSMB10	16
71	cg09014626	2.80273	0.00586539	0.87238	0.857221	-1.5188	5.81E-06	8.52E-05				5
72	cg14231974	-2.80645	0.00590265	0.83109	0.84464	2.087894	9.30E-10	2.71E-08		Body	ODZ3	4
73	cg08774350	2.79594	0.00596497	0.93448	0.927882	-1.41638	2.24E-05	0.0002889		Body	SCFD2	4
74	cg24723421	2.79154	0.00602916	0.88227	0.865479	-1.3398	5.88E-05	0.0006856		Body;Body;B	FN1;FN1;FN1;FN1	2
75	cg06971253	-2.79402	0.00604493	0.05524	0.063168	-1.68149	5.87E-07	1.05E-05	Island	1stExon;5'U	JUB;JUB	14
76	cg16872847	-2.79084	0.00604628	0.06738	0.072611	-1.36898	4.09E-05	0.0004949	Island			19
77	cg26878734	2.79594	0.00608669	0.86476	0.833882	-2.09756	7.88E-10	2.32E-08		3'UTR	KIAA1715	2
78	cg18693004	2.78773	0.00615388	0.88825	0.872844	-1.66867	7.08E-07	1.25E-05		Body	SP4	7
79	cg11263705	-2.78635	0.00617166	0.05504	0.059477	-1.63024	1.23E-06	2.07E-05	Island	3'UTR;1stEx	IER5;IER5	1
80	cg08568987	-2.78368	0.00617475	0.05505	0.062639	-1.3758	3.76E-05	0.0004589	Island	TSS200;TSS2	U2AF2;U2AF2	19
81	cg26561082	2.78694	0.00621279	0.86557	0.842927	-1.91223	1.70E-08	4.03E-07	S_Shore	Body	DIP2C	10
82	cg02559274	2.7779	0.0062799	0.92571	0.917184	-1.37968	3.58E-05	0.0004396		Body	EXOC6B	2
83	cg22921703	2.77559	0.00635153	0.85913	0.850961	-1.54519	4.05E-06	6.14E-05		1stExon	PSMB11	14
84	cg09894631	2.77016	0.0064206	0.8969	0.886624	-1.36898	4.09E-05	0.0004949				16
85	cg14716933	2.7729	0.00642095	0.86624	0.856532	-1.65367	8.80E-07	1.52E-05	N_Shore	TSS1500	PSMB6	17
86	cg20691612	2.76726	0.00646632	0.88474	0.86824	-1.32069	7.42E-05	0.0008428		Body;Body	MAST4;MAST4	5

87	cg05822618	2.77179	0.00650529	0.90258	0.882591	-1.99199	4.65E-09	1.21E-07		Body	FAM20B	1
88	cg05942111	-2.76331	0.00657877	0.75502	0.777648	1.533374	4.77E-06	7.12E-05		Body	RASSF3	12
89	cg16270148	-2.77225	0.00661248	0.86689	0.878959	2.627111	4.48E-14	2.57E-12		Body	GTF2A2	15
90	cg22772462	2.75991	0.00661334	0.9064	0.89559	-1.36584	4.25E-05	0.0005124	S_Shore	TSS1500	TNIP1	5
91	cg15710961	-2.75423	0.00674738	0.84872	0.862347	1.501483	7.34E-06	0.0001054		Body;Body;E	DST;DST;DST;DST	6
92	cg14611258	-2.74473	0.00690012	0.83178	0.8468	1.315559	7.90E-05	0.0008909	S_Shelf	Body	C17orf39	17
93	cg08470053	2.74189	0.00702157	0.84727	0.836773	-1.66047	7.97E-07	1.39E-05	N_Shore	5'UTR;5'UTR	ZNF160;ZNF160;Z	19
94	cg10456121	-2.73691	0.00707254	0.80353	0.815979	1.40006	2.76E-05	0.0003485	Island			2
95	cg04169881	-2.73442	0.00716724	0.0456	0.050267	-1.62899	1.25E-06	2.10E-05	Island	5'UTR;1stEx	TUT1;TUT1	11
96	cg09634659	2.72969	0.00721182	0.89615	0.887441	-1.35323	4.98E-05	0.0005898				1
97	cg15681326	2.72976	0.00726848	0.91299	0.901247	-1.65438	8.71E-07	1.51E-05		3'UTR;3'UTR	NCEH1;NCEH1;NC	3
98	cg10596956	2.7247	0.00740024	0.87554	0.853091	-1.78337	1.28E-07	2.60E-06		TSS200	OR52M1	11
99	cg25597667	2.71692	0.00750736	0.93026	0.923214	-1.49345	8.17E-06	0.0001162		Body;Body;E	ZNF160;ZNF160;Z	19
100	cg25726128	-2.70581	0.00772201	0.84163	0.856913	1.358021	4.69E-05	0.0005593	N_Shelf	Body	ICOSLG	21
101	cg21240492	-2.70218	0.00782095	0.03398	0.039434	-1.45327	1.39E-05	0.0001877	Island	1stExon;5'U	EIF4A1;EIF4A1	17
102	cg19651199	2.70665	0.0078339	0.90567	0.895025	-1.9949	4.43E-09	1.16E-07	Island			18
103	cg16760843	2.70033	0.00784475	0.93065	0.924477	-1.3641	4.35E-05	0.0005225	N_Shore	Body	RASA3	13
104	cg20738274	-2.70229	0.00788481	0.74003	0.761129	1.774982	1.46E-07	2.92E-06		5'UTR;5'UTR	C1orf116;C1orf11	1
105	cg25334215	2.69962	0.00802046	0.91827	0.903867	-2.13386	4.22E-10	1.30E-08		3'UTR;Body;	CSTF3;CSTF3;CSTI	11
106	cg09481537	2.69034	0.00814094	0.93631	0.929445	-1.70622	4.08E-07	7.54E-06				12
107	cg07076091	2.70022	0.00815618	0.9042	0.87757	-2.89426	2.00E-16	1.62E-14		Body;Body	SC4MOL;SC4MOL	4
108	cg19091779	-2.68605	0.00818256	0.74143	0.762562	1.430316	1.87E-05	0.0002459	S_Shelf	Body	KIAA1467	12
109	cg14163966	2.6898	0.00821787	0.91312	0.902742	-2.00615	3.68E-09	9.76E-08	S_Shore	TSS1500	C1orf89	1
110	cg04707299	2.68201	0.00828019	0.89354	0.878284	-1.44844	1.48E-05	0.000199		3'UTR	PEX3	6
111	cg19486507	2.68359	0.00828411	0.86065	0.849841	-1.64269	1.03E-06	1.76E-05	Island	Body;Body	CABP2;CABP2	11
112	cg02660165	-2.68085	0.00828948	0.81438	0.828903	1.359624	4.60E-05	0.0005493	S_Shore	Body	VANGL2	1
113	cg16660345	-2.67824	0.00836148	0.05135	0.055645	-1.41179	2.38E-05	0.0003046	Island	TSS1500;TSS	FGFRL1;FGFRL1	4
114	cg14983306	2.67719	0.0083865	0.81504	0.804266	-1.41312	2.34E-05	0.0003	S_Shelf			7
115	cg06872243	-2.67604	0.0084265	0.04119	0.045257	-1.4747	1.05E-05	0.0001455	S_Shore	Body;Body;T	LOC81691;LOC81	16
116	cg19442480	-2.66991	0.00853919	0.01961	0.022323	-1.30687	8.77E-05	0.0009775	Island	5'UTR;1stEx	DDX39;DDX39	19

117	cg06902828	2.66961	0.0085504	0.88465	0.867876	-1.32784	6.80E-05	0.0007801				8
118	cg14819132	-2.6686	0.00858354	0.04393	0.048067	-1.37016	4.03E-05	0.0004886	Island	TSS200	PEMT	17
119	cg18450540	2.66851	0.00868524	0.92744	0.915861	-1.83203	6.05E-08	1.30E-06		3'UTR;3'UTR	MCM8;MCM8	20
120	cg12937434	-2.66315	0.0087129	0.06117	0.070706	-1.35345	4.96E-05	0.0005884	S_Shelf	1stExon;5'U	BZRAP1;BZRAP1;E	17
121	cg13459205	-2.66772	0.00872848	0.03413	0.037947	-1.93969	1.09E-08	2.67E-07	Island	Body;Body	FAM189B;FAM18	1
122	cg15325154	2.66094	0.00883528	0.8627	0.844876	-1.66976	6.97E-07	1.23E-05		Body;TSS20(	ZNF804B;MGC26(	7
123	cg00268644	2.65736	0.00886579	0.91888	0.911211	-1.40004	2.76E-05	0.0003486	S_Shore	TSS1500	FERMT1	20
124	cg04309375	-2.65457	0.00896041	0.80415	0.815156	1.516669	5.98E-06	8.75E-05		Body;Body;E	ACOT7;ACOT7;AC	1
125	cg23149560	-2.65338	0.00900439	0.04902	0.054466	-1.57975	2.51E-06	3.97E-05	S_Shore	TSS1500	MOBKL1B	2
126	cg27075917	-2.65214	0.00908339	0.84034	0.851947	1.7926	1.11E-07	2.28E-06	N_Shelf			17
127	cg06663648	-2.64747	0.00909738	0.0355	0.039363	-1.31437	8.01E-05	0.0009022		Body	UQCRC2	16
128	cg05602439	-2.64621	0.00924539	0.79334	0.808888	1.837425	5.56E-08	1.20E-06		5'UTR;TSS15	CREM;CREM;CREI	10
129	cg22889620	-2.64006	0.0093026	0.84275	0.855755	1.382859	3.44E-05	0.0004242	N_Shelf	Body	TMED10	14
130	cg26323746	2.64881	0.00934744	0.88838	0.870821	-2.59424	8.55E-14	4.69E-12	N_Shelf			19
131	cg20033401	2.64133	0.00936879	0.94067	0.933819	-1.82399	6.86E-08	1.46E-06	N_Shore	Body;Body;E	ABHD11;ABHD11;	7
132	cg07166601	2.63536	0.00944192	0.87111	0.859673	-1.45696	1.32E-05	0.0001797		Body	FLRT2	14
133	cg20898305	2.63233	0.00964654	0.91176	0.904539	-1.99524	4.41E-09	1.15E-07		Body	TSSC1	2
134	cg21783047	2.63436	0.00966326	0.17122	0.149953	2.301355	2.16E-11	8.24E-10	N_Shore			12
135	cg21239317	-2.63918	0.00975112	0.7994	0.818727	3.404033	3.20E-21	5.33E-19	S_Shelf	5'UTR	CCDC126	7
136	cg10534873	-2.62466	0.0097664	0.74921	0.771199	1.625659	1.32E-06	2.19E-05		Body	PELI2	14
137	cg21233506	2.62558	0.00979957	0.93186	0.920489	-1.87241	3.21E-08	7.24E-07		Body	ACVR2A	2
138	cg03228985	2.62072	0.00981702	0.89602	0.887051	-1.37602	3.75E-05	0.0004578				1
139	cg17759806	2.62098	0.00987005	0.9154	0.908226	-1.64042	1.06E-06	1.81E-05	Island			1
140	cg18098053	2.61976	0.00992381	0.88656	0.874635	-1.72534	3.07E-07	5.80E-06				5
141	cg06030649	-2.62377	0.00994035	0.04127	0.046663	-2.26033	4.53E-11	1.64E-09	Island	Body	HEXB	5
142	cg12682862	-2.61509	0.00999221	0.01872	0.02132	-1.46587	1.18E-05	0.0001616	Island	5'UTR;1stEx(	RARS;RARS	5
143	cg18838346	-2.61696	0.01000048	0.0481	0.053722	-1.72306	3.18E-07	5.98E-06	S_Shore	Body;Body;E	C6orf136;C6orf13	6
144	cg00053927	2.61382	0.0100018	0.89399	0.88395	-1.35129	5.10E-05	0.0006027	N_Shelf	Body;Body	MCF2L;MCF2L	13
145	cg00987136	-2.61601	0.0100035	0.87542	0.885868	1.625372	1.32E-06	2.20E-05		3'UTR;Body	PSMB7;LOC10012	9
146	cg19784622	2.61305	0.01002675	0.91355	0.908857	-1.36833	4.12E-05	0.0004984		Body	DOCK1	10

147	cg10876928	-2.61783	0.01006604	0.73401	0.753552	2.097193	7.93E-10	2.34E-08	S_Shore	TSS1500	SDC4	20
148	cg06970076	2.60975	0.01014219	0.89128	0.88382	-1.47048	1.11E-05	0.000153	S_Shore	3'UTR	IFT140	16
149	cg13725340	2.6059	0.01023667	0.91332	0.902994	-1.4081	2.49E-05	0.0003177		Body	C9orf171	9
150	cg10730291	2.60406	0.01029223	0.87932	0.870628	-1.4215	2.10E-05	0.0002724	S_Shelf	Body	FKBP4	12
151	cg18625289	-2.60319	0.01031191	0.54612	0.563086	1.399856	2.77E-05	0.0003493	N_Shore	Body	MBD3	19
152	cg01770400	-2.60301	0.01033259	0.87629	0.888793	1.466776	1.16E-05	0.0001598		5'UTR;1stEx	SERPINC1;SERPIN	1
153	cg26321126	2.60365	0.01033507	0.88653	0.873832	-1.55577	3.50E-06	5.38E-05		Body	MEGF11	15
154	cg13073030	2.60196	0.01037044	0.87375	0.85555	-1.50041	7.44E-06	0.0001068				14
155	cg14517119	2.59715	0.01047305	0.84	0.820093	-1.34416	5.57E-05	0.0006533		Body	HCN1	5
156	cg10786880	-2.59678	0.01048934	0.02315	0.026641	-1.36841	4.12E-05	0.0004979	Island	1stExon;1stE	PRKAA1;PRKAA1	5
157	cg02335700	-2.59655	0.01054425	0.85721	0.86894	1.574117	2.72E-06	4.26E-05	N_Shore			12
158	cg03965827	-2.59961	0.01055246	0.04493	0.050514	-1.97103	6.56E-09	1.67E-07	S_Shore	TSS1500	TRPM7	15
159	cg25368824	-2.59515	0.01057526	0.76816	0.789965	1.533402	4.76E-06	7.12E-05		TSS200;TSS2	IL4;IL4	5
160	cg17205233	2.59073	0.01068756	0.92534	0.918627	-1.46083	1.26E-05	0.0001717		Body;Body	MIR548F5;DCLK1	13
161	cg17537403	-2.58828	0.01074308	0.07139	0.077506	-1.3882	3.21E-05	0.0003991	Island	TSS200	GPR160	3
162	cg23537419	-2.59126	0.01087278	0.7676	0.787107	2.2774	3.33E-11	1.23E-09	S_Shelf	Body;Body	KCP;KCP	7
163	cg12229979	-2.58495	0.01088799	0.74733	0.76819	1.57997	2.51E-06	3.96E-05	S_Shelf	5'UTR;5'UTR	MYO9B;MYO9B	19
164	cg03859684	-2.58179	0.01093313	0.03119	0.035794	-1.37262	3.91E-05	0.0004754	Island	TSS1500	ANKRD10	13
165	cg26523175	-2.5894	0.01093755	0.76335	0.781574	2.315298	1.67E-11	6.50E-10		TSS1500	PDZK1IP1	1
166	cg16243195	-2.57818	0.01115773	0.8211	0.835255	1.840789	5.28E-08	1.14E-06		Body	SORCS2	4
167	cg02708341	-2.57898	0.01122621	0.04138	0.048275	-2.20758	1.16E-10	3.94E-09	Island	TSS1500;TSS	PTMA;PTMA	2
168	cg13433729	-2.57187	0.01125995	0.6689	0.690287	1.473907	1.06E-05	0.0001468		Body;Body	EZR;EZR	6
169	cg12045715	-2.57487	0.01132037	0.78297	0.800608	2.080391	1.06E-09	3.05E-08	S_Shelf	Body	C19orf22	19
170	cg00280761	-2.56845	0.01143261	0.02944	0.034166	-1.73923	2.50E-07	4.79E-06	Island	Body	DGCR14	22
171	cg06630567	2.56713	0.01146228	0.92476	0.919354	-1.69465	4.84E-07	8.82E-06		1stExon	AMBP	9
172	cg24469412	2.57197	0.01147076	0.85424	0.833022	-2.32083	1.51E-11	5.91E-10	S_Shore	TSS1500;3'U	LYPD1;NCKAP5;N	2
173	cg06925387	-2.56424	0.0115187	0.08854	0.103123	-1.55776	3.41E-06	5.25E-05	N_Shore	5'UTR	TRIM68	11
174	cg05187247	2.56894	0.0116208	0.91913	0.901436	-2.54823	2.09E-13	1.09E-11		TSS1500;TSS	SNORD114-29;SN	14
175	cg24997562	-2.5583	0.01165252	0.04044	0.044761	-1.33592	6.16E-05	0.0007152		1stExon;1stE	OASL;OASL	12
176	cg11817804	2.55877	0.01169673	0.8088	0.789326	-1.57604	2.65E-06	4.16E-05	N_Shelf	Body	KIAA0895	7

177	cg13529101	2.55817	0.01179686	0.92855	0.924077	-1.88927	2.46E-08	5.66E-07		Body;Body;E	CUX1;CUX1;CUX1	7
178	cg10213059	2.5534	0.01187509	0.89639	0.885146	-1.59905	1.92E-06	3.10E-05		Body	KLHL1	13
179	cg17758905	2.54633	0.01208202	0.86157	0.846142	-1.50557	6.95E-06	0.0001003		TSS1500	SKINTL	1
180	cg14110548	-2.54519	0.01212061	0.34033	0.354853	-1.50922	6.61E-06	9.59E-05	S_Shelf	Body;Body	HCST;HCST	19
181	cg18553202	2.5435	0.01216695	0.88015	0.863811	-1.4719	1.09E-05	0.0001504		TSS200	LOC649395	7
182	cg10006897	-2.54448	0.01217491	0.8535	0.863525	1.62963	1.24E-06	2.08E-05	S_Shelf	Body;Body	PCBP3;PCBP3	21
183	cg14914246	2.54524	0.01217904	0.921	0.912414	-1.74124	2.42E-07	4.67E-06				11
184	cg25152115	2.54839	0.01218314	0.9329	0.923513	-2.14965	3.21E-10	1.01E-08				2
185	cg03977443	-2.54447	0.0122175	0.85758	0.867145	1.789951	1.16E-07	2.37E-06		Body	ACSL1	4
186	cg03406131	2.54578	0.01231623	0.86884	0.850985	-2.33027	1.27E-11	5.03E-10				17
187	cg01166180	-2.53593	0.01240996	0.05353	0.058157	-1.43262	1.82E-05	0.0002394	Island	TSS1500;1st	POLR2I;TBCB	19
188	cg01420889	2.53785	0.01250473	0.86757	0.853637	-2.03623	2.23E-09	6.13E-08	S_Shelf	Body;Body;E	GFRA2;GFRA2;GF	8
189	cg05100846	-2.53476	0.01251916	0.81148	0.822622	1.700082	4.47E-07	8.20E-06		Body;Body;E	WDR20;WDR20;V	14
190	cg13398658	-2.53226	0.01254629	0.83587	0.846456	1.480815	9.67E-06	0.0001352		Body;Body;E	ZDHHC3;ZDHHC3	3
191	cg15043711	-2.53547	0.01257228	0.06349	0.072307	-1.98787	4.98E-09	1.29E-07	S_Shore	3'UTR;1stEx	HSPA1B;HSPA1B	6
192	cg03379894	-2.53134	0.01261381	0.83465	0.846911	1.618576	1.45E-06	2.41E-05	N_Shore			5
193	cg24312843	2.53165	0.01268467	0.87268	0.860914	-1.92134	1.47E-08	3.51E-07				13
194	cg05359518	2.52496	0.01276297	0.87474	0.859253	-1.34648	5.41E-05	0.0006363		Body;Body;E	TCF12;TCF12;TCF	15
195	cg19850728	-2.52632	0.01277978	0.6481	0.66576	1.59382	2.06E-06	3.31E-05	N_Shelf	5'UTR;5'UTR	MINA;MINA;MIN	3
196	cg01122318	2.52472	0.01280282	0.90328	0.891495	-1.47072	1.11E-05	0.0001525	N_Shore	TSS1500	NIPSNAP3B	9
197	cg25960567	-2.52336	0.01280834	0.03956	0.044662	-1.30631	8.83E-05	0.0009835	N_Shore	Body	ITPR2	12
198	cg13991631	-2.52661	0.01281632	0.79753	0.808642	1.766843	1.65E-07	3.27E-06				5
199	cg09681286	-2.52601	0.01286021	0.86907	0.878711	1.85209	4.42E-08	9.74E-07	Island	Body	ABCA3	16
200	cg22949004	-2.5225	0.01289419	0.81781	0.826892	1.525467	5.31E-06	7.85E-05		Body;Body;5	NUP62;NUP62;IL	19
201	cg13829638	-2.51797	0.01300787	0.80242	0.813723	1.351568	5.08E-05	0.000601	N_Shelf	Body	RPL35	9
202	cg04152151	2.51984	0.01305116	0.88348	0.865054	-1.76214	1.77E-07	3.49E-06		TSS200	MBD5	2
203	cg19642007	-2.5171	0.01306769	0.83941	0.849672	1.465082	1.19E-05	0.0001631	N_Shelf	Body;Body;E	TNNT3;TNNT3;TN	11
204	cg19788600	-2.51925	0.01308961	0.07384	0.080028	-1.82608	6.64E-08	1.42E-06	Island	Body	ZNF429	19
205	cg08647091	-2.52129	0.01313978	0.8183	0.829431	2.274042	3.54E-11	1.30E-09		Body	GLIS2	16
206	cg14472810	2.51044	0.01327626	0.69143	0.674342	-1.35476	4.88E-05	0.00058	N_Shore	Body;Body;E	MAD1L1;MAD1L1	7

207	cg22038333	2.5144	0.01334162	0.89472	0.877171	-2.11598	5.75E-10	1.74E-08				6
208	cg11676109	-2.50738	0.01338492	0.05009	0.056485	-1.34953	5.21E-05	0.0006148	Island	5'UTR;5'UTR	HINFP;HINFP	11
209	cg11727029	-2.50551	0.01349189	0.0311	0.036816	-1.49985	7.50E-06	0.0001076	N_Shore	TSS200;5'UT	UNC45A;UNC45A	15
210	cg22126690	-2.50244	0.01362057	0.06565	0.070632	-1.56105	3.26E-06	5.04E-05	Island	TSS200	EEF1G	11
211	cg16175294	2.50375	0.01365515	0.84191	0.823409	-1.85752	4.06E-08	8.99E-07		Body;Body;E	CD44;CD44;CD44	11
212	cg25684392	2.50497	0.01368387	0.92171	0.911703	-2.11954	5.40E-10	1.64E-08	N_Shelf			6
213	cg10156739	2.49823	0.01372254	0.90752	0.899059	-1.36369	4.37E-05	0.0005249		TSS200	LOC100287718	6
214	cg20201971	2.50493	0.01385307	0.89483	0.875512	-2.75458	3.51E-15	2.38E-13	S_Shore			2
215	cg13726814	-2.49438	0.01388408	0.86497	0.877453	1.43461	1.77E-05	0.0002339		Body	SCTR	2
216	cg16143325	-2.49106	0.01398945	0.73025	0.75248	1.364256	4.34E-05	0.0005216	N_Shore			3
217	cg23352747	-2.49158	0.01399889	0.09854	0.105954	-1.47164	1.09E-05	0.0001509	N_Shore	TSS1500	ALPL	1
218	cg12617225	-2.49149	0.01402744	0.78262	0.795173	1.563426	3.15E-06	4.89E-05				6
219	cg12135769	2.48993	0.01405347	0.91607	0.903709	-1.44427	1.56E-05	0.000209	S_Shelf	5'UTR;5'UTR	ZNF197;ZNF197	3
220	cg17032005	-2.49315	0.01409663	0.84997	0.860429	2.028113	2.55E-09	6.95E-08				1
221	cg17877485	2.49452	0.01413285	0.88213	0.859899	-2.34228	1.02E-11	4.09E-10		3'UTR;3'UTR	CCNG1;CCNG1	5
222	cg04266873	2.48926	0.01414184	0.93689	0.928696	-1.67261	6.69E-07	1.18E-05		3'UTR;3'UTR	LUC7L3;LUC7L3	17
223	cg10214968	2.48838	0.01419011	0.84235	0.820852	-1.72539	3.07E-07	5.80E-06	N_Shelf			16
224	cg10746447	-2.4844	0.01425493	0.80101	0.819957	1.41451	2.30E-05	0.0002952		Body	MAML2	11
225	cg22819824	2.48771	0.01425816	0.89493	0.873152	-1.87334	3.17E-08	7.14E-07	S_Shelf	Body	NHLRC2	10
226	cg15558969	2.48481	0.01427752	0.94163	0.934698	-1.5539	3.60E-06	5.51E-05				2
227	cg06220521	-2.48304	0.01435762	0.034	0.038299	-1.59715	1.97E-06	3.17E-05	Island	1stExon	GPR180	13
228	cg10665848	2.47957	0.01441425	0.86108	0.845449	-1.31561	7.89E-05	0.0008904		Body;Body;E	MOG;MOG;MOG;	6
229	cg19813724	2.47941	0.01449377	0.93479	0.924546	-1.5841	2.36E-06	3.75E-05	N_Shelf			13
230	cg06800421	2.47514	0.01459526	0.91726	0.908987	-1.35138	5.09E-05	0.0006022		Body	CPEB3	10
231	cg13720472	-2.47583	0.01462689	0.08649	0.094074	-1.56294	3.17E-06	4.92E-05	Island	TSS1500	ICAM1	19
232	cg11979779	2.4771	0.0146713	0.93185	0.925303	-1.88938	2.45E-08	5.65E-07		Body;Body;E	CAMKK1;CAMKK1	17
233	cg16653495	-2.48005	0.01468068	0.04222	0.0473	-2.32108	1.51E-11	5.89E-10	N_Shore			2
234	cg14340619	-2.4763	0.0147616	0.88328	0.89469	2.09249	8.60E-10	2.52E-08	N_Shelf			3
235	cg02157002	2.47112	0.01476761	0.9108	0.903906	-1.4054	2.58E-05	0.0003278				1
236	cg02786267	2.47108	0.01477264	0.90591	0.896527	-1.41808	2.20E-05	0.0002832		TSS1500	HLA-DQA2	6

237	cg18965322	2.47104	0.0147743	0.91041	0.898737	-1.41833	2.19E-05	0.0002824		5'UTR;5'UTR	TSGA10;TSGA10	2
238	cg02985445	-2.47699	0.01483003	0.78135	0.800392	2.429337	2.02E-12	9.06E-11	N_Shore			7
239	cg25400358	-2.46879	0.01486811	0.80014	0.811721	1.435598	1.75E-05	0.0002313	N_Shore	Body;Body;1	GPR137;GPR137;(	11
240	cg15032393	-2.468	0.01487444	0.04809	0.052068	-1.34424	5.56E-05	0.0006527	Island	TSS1500	ZNF8	19
241	cg16162536	2.46918	0.01487558	0.84145	0.827041	-1.51788	5.88E-06	8.62E-05	Island	Body	LOC100133612	1
242	cg09500565	2.46718	0.01494475	0.92347	0.915166	-1.48117	9.62E-06	0.0001346		Body;Body	REV1;REV1	2
243	cg27271298	2.46402	0.01505677	0.88636	0.872147	-1.43147	1.85E-05	0.0002426		Body;Body	RIMS2;RIMS2	8
244	cg18238718	2.46386	0.01508222	0.89895	0.891878	-1.49961	7.52E-06	0.0001079	Island	Body	ZNF362	1
245	cg18553223	-2.46328	0.01509841	0.04882	0.054336	-1.47384	1.06E-05	0.0001469		TSS1500;TSS	RAP1GAP2;RAP1(	17
246	cg08171933	2.46346	0.01515979	0.88221	0.87269	-1.71189	3.75E-07	6.97E-06		Body	ATF6	1
247	cg23419328	-2.4713	0.01525778	0.02934	0.033715	-3.24634	1.06E-19	1.40E-17	Island			6
248	cg17955635	-2.45813	0.01529675	0.02304	0.025323	-1.44003	1.65E-05	0.0002197	S_Shore	TSS1500	ZBTB44	11
249	cg09439260	2.45747	0.01530031	0.92885	0.918872	-1.35563	4.83E-05	0.0005745	N_Shore	Body	DAZL	3
250	cg21188731	-2.46131	0.01535079	0.8258	0.84075	2.063986	1.40E-09	3.96E-08	S_Shelf	Body	TMPRSS6	22
251	cg17853089	2.45474	0.01548623	0.87226	0.864607	-1.61765	1.47E-06	2.44E-05		Body;Body;E	ABLIM2;ABLIM2;/	4
252	cg02527375	-2.45149	0.01563422	0.87461	0.882382	1.664799	7.49E-07	1.31E-05		Body	ULK4	3
253	cg07808859	2.45178	0.01565789	0.90598	0.893766	-1.78478	1.25E-07	2.55E-06				5
254	cg18663874	-2.45142	0.01566581	0.04469	0.048619	-1.7613	1.79E-07	3.53E-06	N_Shore	Body;TSS15(	RAD1;BRIX1;RAD:	5
255	cg06795425	2.45024	0.01567331	0.88445	0.876156	-1.62265	1.37E-06	2.28E-05				2
256	cg17125141	2.44673	0.01575584	0.82821	0.819652	-1.40313	2.66E-05	0.0003364		Body;Body	AGAP1;AGAP1	2
257	cg03716388	2.45264	0.01581465	0.88457	0.869973	-2.43428	1.84E-12	8.31E-11		Body;Body	MDFIC;MDFIC	7
258	cg05722371	2.44523	0.01592137	0.90437	0.895148	-1.75202	2.06E-07	4.02E-06		3'UTR;3'UTR	PEX19;PEX19	1
259	cg13189979	2.44651	0.01594331	0.87475	0.86302	-2.0029	3.88E-09	1.03E-07		Body	PTPRQ	12
260	cg15492982	-2.4448	0.01597262	0.86512	0.876266	1.862864	3.73E-08	8.32E-07	S_Shelf			6
261	cg07124686	-2.43982	0.01602089	0.06655	0.072312	-1.31105	8.34E-05	0.0009347	Island	Body;TSS15(	RGS20;RGS20	8
262	cg15248889	-2.44026	0.01602634	0.80432	0.822601	1.396492	2.89E-05	0.0003632	S_Shelf			4
263	cg19768607	2.43778	0.01613801	0.90714	0.900697	-1.41854	2.18E-05	0.0002817	N_Shelf			11
264	cg02570501	2.43868	0.01621143	0.86223	0.841358	-1.79495	1.07E-07	2.21E-06	N_Shore	TSS1500;TSS	ZNF107;ZNF107	7
265	cg23316191	-2.44569	0.01628036	0.8032	0.821414	3.101167	2.51E-18	2.67E-16				6
266	cg08838602	2.43323	0.01633769	0.91086	0.894645	-1.43547	1.75E-05	0.0002316				8

267	cg14198950	2.43332	0.01639198	0.91026	0.905179	-1.63167	1.21E-06	2.03E-05	N_Shore	Body	DENND4B	1
268	cg17959962	2.43679	0.01640806	0.89938	0.885279	-2.17325	2.13E-10	6.89E-09		Body	TMTC1	12
269	cg03439065	2.43211	0.01644967	0.89384	0.884242	-1.64998	9.28E-07	1.60E-05		Body	EYA3	1
270	cg20001666	-2.43475	0.01646355	0.88449	0.894902	2.067881	1.31E-09	3.72E-08	S_Shelf	3'UTR;3'UTR	KIF17;KIF17	1
271	cg18023771	-2.43593	0.01654948	0.9016	0.912596	2.529416	3.01E-13	1.53E-11		Body;Body;E	OSBPL3;OSBPL3;(	7
272	cg07118196	-2.42984	0.01655998	0.85481	0.864865	1.688273	5.32E-07	9.60E-06		5'UTR	ANKRD11	16
273	cg07575204	2.42987	0.01659846	0.85926	0.845515	-1.81787	7.54E-08	1.60E-06	N_Shelf	Body	EFNA5	5
274	cg24089400	2.43107	0.01663893	0.89022	0.873065	-2.11923	5.43E-10	1.65E-08	N_Shelf			1
275	cg18125326	2.42989	0.01668482	0.90624	0.894493	-2.10102	7.43E-10	2.20E-08	S_Shelf			3
276	cg26210364	-2.42604	0.016747	0.73391	0.750569	1.755746	1.95E-07	3.81E-06				11
277	cg10309642	2.42196	0.01686891	0.9215	0.912177	-1.56645	3.02E-06	4.70E-05		Body;Body;E	DYNC1I1;DYNC1I1	7
278	cg08128826	-2.42018	0.01691006	0.15131	0.175206	-1.4404	1.65E-05	0.0002188		Body;Body	LHX9;LHX9	1
279	cg11553919	2.41996	0.01700277	0.86876	0.847555	-1.71362	3.66E-07	6.81E-06		3'UTR;3'UTR	GNRHR;GNRHR	4
280	cg02374294	-2.41598	0.01710385	0.7821	0.793183	1.461611	1.25E-05	0.0001701				5
281	cg08616182	-2.41837	0.01712125	0.84124	0.852695	1.865099	3.61E-08	8.05E-07	N_Shelf	Body	SPATA2L	16
282	cg13441193	2.41657	0.01722933	0.90013	0.886665	-1.95215	8.93E-09	2.22E-07	N_Shelf			6
283	cg16648094	2.41966	0.017261	0.89832	0.885565	-2.5114	4.26E-13	2.10E-11		3'UTR	CLVS1	8
284	cg26085385	2.41191	0.01731777	0.8749	0.865964	-1.56097	3.26E-06	5.04E-05				2
285	cg13312195	-2.41485	0.01735134	0.82349	0.835355	2.093973	8.38E-10	2.46E-08	N_Shelf	Body	TRPM5	11
286	cg00145093	2.40845	0.01741953	0.91056	0.897753	-1.37658	3.72E-05	0.000455				10
287	cg24354784	2.41184	0.0174347	0.86966	0.847361	-1.92567	1.37E-08	3.30E-07	S_Shelf			6
288	cg26068180	-2.40858	0.01748145	0.72992	0.74889	1.601174	1.86E-06	3.02E-05	S_Shelf	Body;5'UTR;	NASP;NASP;NASP	1
289	cg08244656	2.40989	0.01750279	0.88495	0.867066	-1.85904	3.97E-08	8.80E-07		TSS1500	OR13A1	10
290	cg08230658	-2.40572	0.01753146	0.04086	0.044511	-1.33089	6.55E-05	0.0007553	N_Shore	5'UTR;1stEx	ZNF319;ZNF319	16
291	cg11865883	-2.40931	0.01756102	0.04406	0.049866	-1.95935	7.95E-09	1.99E-07	N_Shore	5'UTR;1stEx	TIMM50;TIMM50	19
292	cg04874463	-2.40665	0.01759956	0.72777	0.746223	1.696507	4.71E-07	8.60E-06				16
293	cg18210526	2.4042	0.01762755	0.83922	0.822971	-1.42046	2.13E-05	0.0002756		5'UTR;5'UTR	SYT1;SYT1	12
294	cg16991298	2.40497	0.01763018	0.95251	0.948547	-1.54607	4.01E-06	6.08E-05	S_Shelf	Body	ATP1B2	17
295	cg16177693	-2.40306	0.01773508	0.80789	0.821656	1.600323	1.88E-06	3.05E-05		TSS200	NR0B2	1
296	cg21982660	2.40343	0.01775603	0.87755	0.868959	-1.72096	3.28E-07	6.16E-06		3'UTR;3'UTR	SNX5;SNX5	20

297	cg24980904	-2.40296	0.01779192	0.03875	0.04427	-1.76625	1.66E-07	3.30E-06	S_Shore	5'UTR	MYL12A	18
298	cg22920289	-2.39846	0.01787376	0.05317	0.057757	-1.35246	5.03E-05	0.0005949	Island	TSS200	FEM1A	19
299	cg03724759	-2.39861	0.01788122	0.0422	0.04564	-1.40177	2.70E-05	0.0003417	S_Shore	5'UTR;TSS15	UBXN11;UBXN11,	1
300	cg25140501	-2.39844	0.01788448	0.03139	0.036874	-1.38553	3.32E-05	0.0004117	Island	5'UTR;5'UTR	ACIN1;ACIN1;ACII	14
301	cg05674990	-2.39627	0.0179708	0.04114	0.048633	-1.33402	6.31E-05	0.0007303	Island	Body	MMP25	16
302	cg25002129	-2.39646	0.01798405	0.88555	0.893976	1.408974	2.47E-05	0.0003146		Body;Body;E	NTM;NTM;NTM;N	11
303	cg03906304	2.39583	0.01799188	0.90414	0.894411	-1.33512	6.22E-05	0.0007216				10
304	cg27158011	2.39802	0.01804026	0.86046	0.83925	-1.82135	7.14E-08	1.52E-06		Body	FMN1	15
305	cg12417744	-2.3951	0.01806779	0.66831	0.687674	1.473747	1.06E-05	0.0001471				2
306	cg03442467	-2.39676	0.01809254	0.07597	0.08281	-1.79989	9.95E-08	2.06E-06		Body;TSS20(	PDE4B;PDE4B;PD	1
307	cg17892588	-2.39349	0.01813739	0.06189	0.067139	-1.45331	1.39E-05	0.0001876				8
308	cg26319800	-2.39522	0.01814535	0.88736	0.896932	1.738388	2.53E-07	4.85E-06		TSS200	SLC4A5	2
309	cg11807040	2.39064	0.01825246	0.88982	0.883086	-1.38647	3.28E-05	0.0004072				13
310	cg23011886	-2.38989	0.01827515	0.79541	0.817963	1.34276	5.66E-05	0.0006631	S_Shore	Body;TSS20(	MGAT4B;MGAT4I	5
311	cg18432572	-2.40065	0.01828596	0.89585	0.904244	3.066016	5.34E-18	5.43E-16		Body;Body;E	CYFIP2;CYFIP2;CY	5
312	cg15152201	-2.39124	0.01832433	0.03047	0.033561	-1.70904	3.92E-07	7.25E-06	Island	TSS200	RPS21	20
313	cg26152388	-2.38777	0.01837501	0.06714	0.073274	-1.33814	6.00E-05	0.000698	Island	TSS1500;TSS	PTPN12;PTPN12;I	7
314	cg15287987	2.38754	0.01839462	0.85451	0.832841	-1.36797	4.14E-05	0.0005003				7
315	cg12008613	2.38727	0.01840634	0.8398	0.823494	-1.36401	4.35E-05	0.0005231	N_Shelf	Body;Body;E	MBD1;MBD1;MBI	18
316	cg02332492	-2.38587	0.01855886	0.83921	0.851334	1.638071	1.10E-06	1.87E-05	S_Shore	Body	C8G	9
317	cg14616574	-2.38385	0.0185589	0.06963	0.074969	-1.32239	7.27E-05	0.0008276	S_Shore			3
318	cg07536419	2.38268	0.01864753	0.89011	0.879145	-1.42982	1.89E-05	0.0002473		5'UTR;5'UTR	PC;PC;PC	11
319	cg18160302	-2.38632	0.01870759	0.83113	0.844259	2.163903	2.50E-10	8.02E-09		Body;Body	CADM1;CADM1	11
320	cg27637842	2.38642	0.01874556	0.88624	0.876594	-2.29873	2.26E-11	8.61E-10		3'UTR	ASB5	4
321	cg07870378	-2.37979	0.0187509	0.81418	0.826346	1.307037	8.76E-05	0.0009757	Island	TSS1500	FAM100A	16
322	cg09684245	2.38275	0.01876485	0.8829	0.873663	-1.80845	8.72E-08	1.82E-06		Body	C1orf168	1
323	cg08204155	2.38175	0.01876992	0.90805	0.900329	-1.67453	6.50E-07	1.15E-05	N_Shore	3'UTR	ZDHHC8	22
324	cg14527213	-2.37813	0.01888019	0.05612	0.060389	-1.46443	1.20E-05	0.0001644	Island	1stExon;5'U	MLL5;MLL5;MLL5	7
325	cg01745867	-2.37915	0.01891875	0.08276	0.089052	-1.74082	2.44E-07	4.69E-06	Island	TSS1500	FLOT1	6
326	cg15772797	-2.38006	0.0189269	0.03234	0.036606	-1.90233	2.00E-08	4.66E-07	Island	TSS200	SETD8	12

327	cg24838136	-2.37771	0.0190116	0.85861	0.869038	1.809255	8.61E-08	1.80E-06		Body	SLC12A8	3
328	cg26942952	-2.37437	0.01908278	0.84405	0.858206	1.520548	5.67E-06	8.34E-05	S_Shore			12
329	cg14182730	-2.37738	0.01912513	0.88465	0.893235	2.105103	6.93E-10	2.07E-08				13
330	cg14096899	-2.37284	0.01918505	0.65238	0.669344	1.604406	1.78E-06	2.89E-05				2
331	cg00905312	2.36876	0.01938013	0.92929	0.923131	-1.57807	2.57E-06	4.06E-05		Body	TRIP12	2
332	cg15906409	-2.3666	0.01941874	0.07092	0.083283	-1.35646	4.78E-05	0.0005693	Island	5'UTR	RAI1	17
333	cg18857216	-2.36865	0.01942688	0.06979	0.078575	-1.70395	4.22E-07	7.78E-06		Body;Body	C1orf127;C1orf12	1
334	cg04305912	2.36706	0.01949	0.836	0.813516	-1.65347	8.82E-07	1.52E-05	N_Shelf	3'UTR;Body;	BCL11A;BCL11A;E	2
335	cg14513448	-2.36647	0.01952894	0.79729	0.809831	1.682247	5.81E-07	1.04E-05	S_Shore			9
336	cg14201717	2.36651	0.01956678	0.85873	0.849432	-1.80126	9.74E-08	2.02E-06				7
337	cg02100365	2.3675	0.01958452	0.91472	0.898542	-2.00515	3.74E-09	9.92E-08	N_Shore	Body	GLMN	1
338	cg27281765	2.366	0.01966546	0.90013	0.886926	-2.0214	2.85E-09	7.72E-08		Body	PMP2	8
339	cg14563115	2.36151	0.01970517	0.9125	0.899959	-1.45028	1.45E-05	0.0001946		Body;Body;5	PRDM2;PRDM2;P	1
340	cg22918949	-2.36563	0.01976039	0.91886	0.9259	2.252048	5.26E-11	1.88E-09				10
341	cg14858951	-2.35965	0.01977258	0.02686	0.031158	-1.36215	4.46E-05	0.0005339	Island	TSS200	NR1D1	17
342	cg20226253	-2.36034	0.01977799	0.86047	0.872136	1.492373	8.29E-06	0.0001177	S_Shore	Body	GDF5	20
343	cg00818480	-2.36079	0.01980582	0.08679	0.096328	-1.64745	9.62E-07	1.65E-05	N_Shore	TSS1500	NXPH4	12
344	cg02336442	-2.36178	0.01981776	0.86451	0.873503	1.835227	5.76E-08	1.24E-06	N_Shore			2
345	cg12318408	-2.36108	0.01984849	0.0519	0.056297	-1.81998	7.30E-08	1.55E-06		1stExon;5'U	RAP1GAP2;RAP1(	17
346	cg19639021	2.36021	0.01988099	0.90641	0.895524	-1.78532	1.24E-07	2.53E-06		Body;Body	MLLT10;MLLT10	10
347	cg13915754	-2.35622	0.01995119	0.06481	0.071074	-1.37107	3.99E-05	0.0004836	Island	Body	SLC32A1	20
348	cg17333424	-2.35534	0.01999364	0.06201	0.06755	-1.36255	4.43E-05	0.0005315	Island	5'UTR;1stEx	RAB5C;RAB5C;RA	17
349	cg23814734	-2.35763	0.02000427	0.89794	0.907275	1.75832	1.87E-07	3.68E-06				10
350	cg19977501	-2.35875	0.02000803	0.85134	0.862953	1.939635	1.10E-08	2.68E-07	N_Shelf			14
351	cg19397801	2.3548	0.02001109	0.92874	0.922277	-1.32837	6.76E-05	0.0007758		3'UTR	PIP4K2A	10
352	cg09849798	2.3602	0.02002598	0.88593	0.87093	-2.21682	9.86E-11	3.38E-09				14
353	cg13455110	2.35505	0.02005077	0.9464	0.94101	-1.49573	7.93E-06	0.000113	Island	3'UTR	PANK4	1
354	cg05954147	2.35827	0.02009091	0.86113	0.837101	-2.11443	5.90E-10	1.78E-08		Body;Body;E	GRM8;GRM8;GRI	7
355	cg01712425	2.35294	0.02012943	0.88289	0.872693	-1.39903	2.80E-05	0.0003528	N_Shelf	Body;Body	GOPC;GOPC	6
356	cg01578265	2.3589	0.02017066	0.84231	0.823818	-2.45878	1.16E-12	5.38E-11				5

357	cg14110885	2.35266	0.02021703	0.90697	0.900794	-1.6247	1.33E-06	2.22E-05		TSS1500	WFIKKN2	17
358	cg03529555	2.35006	0.02025358	0.92527	0.917621	-1.3179	7.68E-05	0.000869		Body	NAV2	11
359	cg07842594	-2.35055	0.02026217	0.03511	0.040588	-1.42613	1.98E-05	0.0002581	Island	5'UTR;1stEx	BMS1;BMS1	10
360	cg11114211	-2.35479	0.02027321	0.85494	0.865797	2.121533	5.22E-10	1.59E-08	S_Shore			7
361	cg26906737	-2.35081	0.02039293	0.65205	0.668712	1.862145	3.78E-08	8.41E-07		Body	TMEM206	1
362	cg05270106	2.34627	0.02047282	0.93035	0.924754	-1.38388	3.39E-05	0.0004194		Body	EEFSEC	3
363	cg08262269	2.34941	0.02048466	0.90865	0.896171	-1.9161	1.60E-08	3.80E-07		Body	STK39	2
364	cg19672209	2.34649	0.02049661	0.90166	0.891628	-1.49447	8.06E-06	0.0001147		TSS1500	C14orf105	14
365	cg11878146	2.34527	0.02065586	0.82545	0.809438	-1.77771	1.40E-07	2.81E-06				7
366	cg13097102	-2.34592	0.02072699	0.86934	0.877785	2.087524	9.36E-10	2.73E-08				1
367	cg20358902	2.34685	0.0207553	0.88058	0.856596	-2.31944	1.55E-11	6.05E-10	N_Shelf	Body;Body	LCORL;LCORL	4
368	cg08919443	2.34072	0.02079059	0.90549	0.89956	-1.4572	1.32E-05	0.0001792	Island	Body	COL6A1	21
369	cg14627538	2.3395	0.02081222	0.8811	0.875044	-1.32104	7.39E-05	0.0008396	S_Shore	Body;Body	PRMT2;PRMT2	21
370	cg19637591	2.3411	0.02082189	0.83394	0.819792	-1.61415	1.55E-06	2.55E-05		Body	PCDH17	13
371	cg12562245	2.33858	0.02088866	0.88989	0.882867	-1.40724	2.52E-05	0.000321		3'UTR	SLC5A5	19
372	cg03292648	2.33801	0.02094196	0.90802	0.902718	-1.47662	1.02E-05	0.0001422	N_Shore	TSS1500	EXO1	1
373	cg27596766	2.34244	0.02094658	0.90791	0.896295	-2.18919	1.61E-10	5.31E-09				2
374	cg07762347	-2.34234	0.02096463	0.84984	0.861542	2.22763	8.14E-11	2.82E-09				7
375	cg01719895	-2.33616	0.0210051	0.06104	0.065739	-1.36547	4.27E-05	0.0005145	Island	5'UTR	CHST12	7
376	cg27661869	-2.33471	0.02107135	0.04635	0.051123	-1.32629	6.93E-05	0.0007933	Island	TSS200	DCAF7	17
377	cg24687051	-2.33709	0.02113256	0.05002	0.05587	-1.89356	2.30E-08	5.31E-07	Island	1stExon;1stE	KCNQ5;KCNQ5;KC	6
378	cg26836382	2.33388	0.02127331	0.84526	0.833982	-1.79867	1.01E-07	2.09E-06	N_Shelf			5
379	cg04557294	-2.3371	0.02139714	0.79413	0.810941	2.694719	1.17E-14	7.30E-13		5'UTR	KIAA0247	14
380	cg06716698	-2.33012	0.02154301	0.73118	0.750131	1.984876	5.23E-09	1.35E-07		5'UTR	TEAD1	11
381	cg22950210	2.32586	0.02158042	0.03656	0.033281	1.409082	2.46E-05	0.0003142	Island	1stExon;5'U	ARHGAP29;ARHG	1
382	cg01178070	2.32561	0.02161611	0.88622	0.871883	-1.47545	1.04E-05	0.0001442		Body	PLXDC2	10
383	cg26964901	2.33051	0.02163175	0.88725	0.872575	-2.30665	1.96E-11	7.53E-10		Body;Body;E	PDE8B;PDE8B;PD	5
384	cg08798148	2.32608	0.02163599	0.92362	0.918926	-1.61073	1.63E-06	2.67E-05	S_Shore	Body	ALKBH4	7
385	cg08268892	-2.33192	0.02165088	0.7927	0.810323	2.602055	7.33E-14	4.06E-12		Body;Body;E	ARID1B;ARID1B;A	6
386	cg10501629	2.3308	0.02166879	0.90144	0.89064	-2.46704	9.93E-13	4.65E-11		1stExon;1stE	ATP2B1;ATP2B1	12

387	cg02756170	2.32742	0.02171519	0.89037	0.878191	-2.05232	1.70E-09	4.76E-08		1stExon	HISPPD1	5
388	cg17407677	-2.32293	0.02172785	0.08894	0.097282	-1.3626	4.43E-05	0.0005312	S_Shore			5
389	cg01324963	-2.33079	0.0217779	0.81866	0.835106	2.815142	1.02E-15	7.46E-14		5'UTR	GPR160	3
390	cg22686716	2.32186	0.02194876	0.91128	0.893219	-1.8383	5.49E-08	1.19E-06		Body;Body	TRIM33;TRIM33	1
391	cg20406758	-2.31932	0.02203093	0.8043	0.820466	1.666773	7.28E-07	1.28E-05		Body;Body;E	KCNQ1;KCNQ1OT	11
392	cg06174606	2.31797	0.02204035	0.91171	0.900132	-1.47144	1.10E-05	0.0001512	S_Shelf			11
393	cg02622809	-2.31877	0.02204847	0.89742	0.905727	1.628342	1.27E-06	2.12E-05	S_Shelf			13
394	cg09514366	-2.31578	0.02210958	0.73669	0.749686	1.306573	8.81E-05	0.0009807				16
395	cg00809917	-2.31659	0.02217007	0.81545	0.825252	1.62498	1.33E-06	2.21E-05		Body	AGPAT4	6
396	cg04826284	-2.31772	0.02226532	0.04857	0.052263	-2.07898	1.08E-09	3.12E-08	Island	TSS200	TMBIM4	12
397	cg01293346	2.3155	0.02237491	0.75802	0.725923	-2.03389	2.32E-09	6.35E-08		3'UTR;1stEx	TAS2R20;TAS2R2	12
398	cg27609489	2.3143	0.02247227	0.87732	0.865606	-2.11668	5.68E-10	1.72E-08		TSS1500	KRTAP4-3	17
399	cg05874309	2.31281	0.0224935	0.91356	0.901232	-1.93548	1.17E-08	2.85E-07		Body;Body;E	CPEB1;CPEB1;CPE	15
400	cg13153796	2.31087	0.02250221	0.90682	0.895339	-1.64744	9.63E-07	1.65E-05		TSS1500	SNORD113-6	14
401	cg04735886	2.31217	0.02251848	0.90833	0.899474	-1.90364	1.95E-08	4.57E-07				3
402	cg00863913	-2.31208	0.02257977	0.90036	0.910146	2.062084	1.44E-09	4.08E-08	N_Shore			8
403	cg21436444	2.30815	0.02261591	0.76262	0.750206	-1.52491	5.35E-06	7.90E-05	Island			18
404	cg27351449	2.31047	0.02262013	0.90912	0.896751	-1.91612	1.60E-08	3.80E-07		3'UTR;3'UTR	AHI1;AHI1;AHI1	6
405	cg21792983	-2.3127	0.02262427	0.88123	0.890252	2.292357	2.54E-11	9.58E-10		Body	SEL1L3	4
406	cg05514531	2.30705	0.02269139	0.87753	0.85533	-1.56161	3.23E-06	5.00E-05		Body	GBE1	3
407	cg23784046	-2.31152	0.02272448	0.78791	0.804228	2.388538	4.34E-12	1.84E-10		TSS1500	HIGD1C	12
408	cg03822267	-2.31322	0.02275651	0.77577	0.793998	2.790322	1.70E-15	1.20E-13	S_Shore	TSS1500	NFKBIE	6
409	cg17501982	-2.30633	0.02278345	0.71239	0.727503	1.705422	4.13E-07	7.62E-06		5'UTR;5'UTR	NCK2;NCK2	2
410	cg24768245	-2.30702	0.02286207	0.06485	0.07111	-2.03985	2.10E-09	5.79E-08	Island	5'UTR;5'UTR	IFNAR2;IFNAR2;IF	21
411	cg16519495	2.3016	0.02293552	0.92422	0.914787	-1.34994	5.18E-05	0.000612		Body;Body	ENAH;ENAH	1
412	cg02039485	-2.30436	0.02294693	0.79189	0.803882	1.844703	4.97E-08	1.08E-06		Body;Body;E	ITPK1;ITPK1;ITPK1	14
413	cg18159684	2.3021	0.02296416	0.89217	0.876531	-1.52189	5.57E-06	8.20E-05		3'UTR;3'UTR	AZIN1;AZIN1	8
414	cg25206451	2.30183	0.02301222	0.85212	0.836374	-1.61425	1.55E-06	2.55E-05		TSS1500	OR2A2	7
415	cg09007244	-2.29904	0.02307591	0.03701	0.04282	-1.32173	7.33E-05	0.0008335	N_Shore	Body;5'UTR;	RPL23AP82;RABL	22
416	cg04664179	-2.30151	0.02320582	0.80495	0.820034	2.106259	6.79E-10	2.03E-08	S_Shelf	Body;Body	PRX;PRX	19

417	cg23731595	-2.29865	0.02321466	0.73683	0.754278	1.661278	7.88E-07	1.37E-05	N_Shore	5'UTR	KIAA1462	10
418	cg16676200	-2.29838	0.02327989	0.77534	0.787259	1.799124	1.01E-07	2.08E-06	N_Shelf			2
419	cg14941654	2.29648	0.02346888	0.85449	0.844434	-2.01385	3.24E-09	8.66E-08	N_Shore	TSS1500	HLA-G	6
420	cg17640684	2.29032	0.02360278	0.87207	0.852549	-1.35741	4.73E-05	0.0005633		Body	LRBA	4
421	cg27536084	2.29136	0.02360693	0.83102	0.81068	-1.55152	3.72E-06	5.68E-05		3'UTR;3'UTR	LARP4;LARP4;LAR	12
422	cg03303300	2.29239	0.0236657	0.92105	0.911202	-1.88626	2.58E-08	5.91E-07		3'UTR	IBTK	6
423	cg22866875	-2.28867	0.02372006	0.58516	0.59924	1.413284	2.33E-05	0.0002994	N_Shelf	Body	XKR6	8
424	cg04782396	-2.28854	0.02373322	0.05011	0.053568	-1.42937	1.90E-05	0.0002486	Island	1stExon	SF3B2	11
425	cg00846968	-2.29046	0.02376519	0.90074	0.909207	1.842737	5.12E-08	1.11E-06	S_Shore	Body;TSS150	NHP2L1;NHP2L1	22
426	cg23280064	-2.28704	0.02382368	0.05184	0.056423	-1.43013	1.88E-05	0.0002464	Island	Body	EDC4	16
427	cg13976610	2.28754	0.02401201	0.90959	0.894978	-2.04061	2.07E-09	5.72E-08				7
428	cg22997768	2.28536	0.02411431	0.84726	0.824573	-1.9599	7.88E-09	1.97E-07		TSS1500;Bo	TAS2R14;PRR4;PF	12
429	cg25061697	2.28268	0.02428737	0.90047	0.892735	-1.98766	4.99E-09	1.29E-07	Island			4
430	cg19982471	-2.28171	0.02430053	0.05164	0.056273	-1.86237	3.76E-08	8.38E-07	N_Shore	Body;5'UTR	MIR1258;ZNF385	2
431	cg22450342	-2.28004	0.02430166	0.68928	0.705892	1.583128	2.40E-06	3.80E-05		Body	MR1	1
432	cg02320356	-2.28198	0.02434888	0.72072	0.738568	2.039733	2.10E-09	5.80E-08				8
433	cg15779843	2.28842	0.02435817	0.91758	0.90247	-3.2874	4.30E-20	6.01E-18	N_Shore	Body	RPA3	7
434	cg04414865	2.28104	0.02441535	0.90965	0.894984	-2.06406	1.39E-09	3.95E-08		Body	BLZF1	1
435	cg11038507	2.27644	0.02449466	0.90244	0.891853	-1.50524	6.98E-06	0.0001007		TSS1500;Bo	MITF;MITF;MITF;	3
436	cg13276570	-2.27903	0.02449678	0.85821	0.879903	1.949547	9.32E-09	2.31E-07	S_Shore	Body	ANKRD33B	5
437	cg09302724	-2.27649	0.02451208	0.85461	0.862694	1.562043	3.21E-06	4.97E-05	N_Shelf	Body;Body;E	TMEM205;TMEM	19
438	cg23641514	2.27781	0.02451353	0.85572	0.840234	-1.79021	1.15E-07	2.36E-06	N_Shelf	Body	TSPAN10	17
439	cg07874484	-2.27531	0.02452383	0.79569	0.806644	1.387649	3.24E-05	0.0004017	S_Shelf	Body;Body	CACNA1H;CACNA	16
440	cg06891858	2.27688	0.02458416	0.92929	0.923507	-1.82788	6.46E-08	1.38E-06	S_Shore	Body	MIB1	18
441	cg17407668	-2.27372	0.0246054	0.03153	0.035293	-1.3382	5.99E-05	0.0006976	Island	TSS200	WIPF2	17
442	cg23590262	2.27768	0.02465536	0.88167	0.861422	-2.15647	2.85E-10	9.05E-09		Body	GALNTL6	4
443	cg24538947	-2.27679	0.02473146	0.08664	0.093908	-2.21578	1.00E-10	3.43E-09	Island	TSS200	C2orf70	2
444	cg20102585	2.2754	0.02473731	0.87641	0.860434	-1.99449	4.46E-09	1.16E-07		TSS1500	MEP1B	18
445	cg13769204	-2.27069	0.02482491	0.04608	0.05122	-1.42999	1.88E-05	0.0002468	Island	Body	GALNT2	1
446	cg26224473	2.27115	0.02491617	0.82035	0.796723	-1.76377	1.73E-07	3.41E-06		TSS1500	PER4	7

447	cg03746115	2.26779	0.02497106	0.81958	0.800283	-1.32834	6.76E-05	0.0007761		3'UTR;3'UTR	ELF2;ELF2	4
448	cg14377370	-2.26773	0.02499284	0.03489	0.039137	-1.3798	3.57E-05	0.000439	Island	TSS1500;1st	NCAPG;DCAF16;D	4
449	cg18544938	2.26811	0.02500308	0.90927	0.901837	-1.47785	1.01E-05	0.0001401		TSS1500	SOSTDC1	7
450	cg26422935	-2.26681	0.02507004	0.8122	0.825851	1.436404	1.73E-05	0.0002292				6
451	cg17315873	2.26665	0.02509403	0.90879	0.899851	-1.47606	1.03E-05	0.0001431				14
452	cg02946185	-2.26654	0.02524358	0.06215	0.072136	-1.8675	3.47E-08	7.78E-07				7
453	cg04972566	-2.26731	0.0252981	0.10867	0.119407	-2.14603	3.42E-10	1.07E-08	Island	Body	FGF19	11
454	cg06826760	-2.26188	0.02534271	0.74601	0.764739	1.322741	7.24E-05	0.0008244	S_Shore	TSS1500	GLRX2	1
455	cg16602816	-2.26156	0.02536976	0.8104	0.821181	1.343525	5.61E-05	0.0006577				5
456	cg02386983	-2.26129	0.02538039	0.06921	0.078987	-1.32355	7.17E-05	0.0008173	Island	5'UTR;5'UTR	KDM3A;KDM3A	2
457	cg23079727	2.26225	0.02544905	0.89837	0.892954	-1.68846	5.30E-07	9.58E-06		3'UTR;3'UTR	VGLL4;ATG7;VGLl	3
458	cg06833564	-2.26167	0.02548828	0.85252	0.864551	1.693556	4.92E-07	8.95E-06	Island	TSS1500	FAM100A	16
459	cg17645598	-2.261	0.02553747	0.71637	0.731684	1.711063	3.80E-07	7.06E-06	S_Shore	Body;Body;E	UBTF;UBTF;UBTF	17
460	cg27388035	2.26215	0.0255566	0.92563	0.913723	-1.96009	7.85E-09	1.97E-07		Body	BLZF1	1
461	cg22976242	2.26067	0.025596	0.84149	0.817516	-1.81229	8.22E-08	1.73E-06	N_Shelf			6
462	cg25415265	2.25818	0.02560122	0.81035	0.794394	-1.38939	3.16E-05	0.0003938	N_Shore			15
463	cg02245534	-2.25836	0.02566671	0.81961	0.831048	1.603621	1.80E-06	2.92E-05	N_Shore	Body	METRNL	17
464	cg02276695	-2.25885	0.0256804	0.06189	0.067271	-1.72748	2.98E-07	5.64E-06	Island	TSS200	ATP13A1	19
465	cg14118453	-2.25598	0.0257241	0.0333	0.037205	-1.33614	6.15E-05	0.0007133	Island			2
466	cg01767599	2.25794	0.02574669	0.90896	0.894388	-1.7482	2.18E-07	4.23E-06	N_Shelf	5'UTR;5'UTR	CNR1;CNR1;CNR1	6
467	cg06584784	2.25783	0.02576162	0.69894	0.686697	-1.76948	1.58E-07	3.15E-06	N_Shelf			16
468	cg15769921	-2.25512	0.02576921	0.03544	0.039293	-1.30594	8.87E-05	0.0009874	Island	Body	TOP2A	17
469	cg24642916	-2.25423	0.02590116	0.03435	0.037885	-1.51849	5.83E-06	8.55E-05	Island	Body;TSS20(	NOTCH4;GPSM3	6
470	cg13647486	-2.25309	0.02590644	0.89477	0.903229	1.324245	7.11E-05	0.0008109				5
471	cg24717078	-2.25402	0.02590697	0.80637	0.817231	1.496834	7.81E-06	0.0001115		Body;Body	KIRREL3;KIRREL3	11
472	cg15174951	-2.25424	0.0259154	0.03982	0.043746	-1.55878	3.36E-06	5.18E-05	S_Shore			6
473	cg16576160	-2.2555	0.02591621	0.87792	0.889166	1.780271	1.34E-07	2.71E-06	S_Shelf			16
474	cg22799510	2.25546	0.02597162	0.82576	0.80337	-1.92251	1.44E-08	3.45E-07		3'UTR;3'UTR	PROK2;PROK2	3
475	cg06618322	-2.25573	0.02604453	0.03979	0.045671	-2.16278	2.55E-10	8.17E-09	Island	TSS200;TSS2	AKAP13;AKAP13	15
476	cg19162768	-2.2533	0.02607582	0.11423	0.124091	-1.8274	6.50E-08	1.39E-06	N_Shore			15

477	cg04905443	2.24965	0.02616597	0.82286	0.799466	-1.42587	1.99E-05	0.0002589		Body;Body	LRP12;LRP12	8
478	cg07519485	-2.24861	0.02622305	0.72183	0.74096	1.394986	2.95E-05	0.0003695		3'UTR;3'UTR	MLN;MLN	6
479	cg15075241	-2.24872	0.02622762	0.77757	0.797142	1.42901	1.91E-05	0.0002496	N_Shore	TSS200;5'UT	SNORA80B;ODC1	2
480	cg02098905	2.24928	0.02624837	0.81969	0.799521	-1.58751	2.25E-06	3.60E-05				8
481	cg19120125	2.25055	0.02629536	0.85815	0.841367	-1.93338	1.21E-08	2.94E-07		TSS200	TAS2R10	12
482	cg12779575	-2.24669	0.02631777	0.83653	0.847791	1.304999	8.97E-05	0.000997	S_Shore	TSS1500	ERN1	17
483	cg17252489	-2.24942	0.02633619	0.78246	0.801317	1.846942	4.80E-08	1.05E-06		Body	STK39	2
484	cg26071414	2.25278	0.0263524	0.89008	0.870948	-2.48501	7.05E-13	3.37E-11		3'UTR	HINT3	6
485	cg20539961	2.25195	0.026457	0.92348	0.917787	-2.6308	4.17E-14	2.40E-12	Island	Body;Body;E	NCK2;NCK2;NCK2	2
486	cg09236780	2.24826	0.02646221	0.94333	0.934274	-1.97786	5.87E-09	1.50E-07		Body	MYO16	13
487	cg21500092	-2.24577	0.02648906	0.79494	0.811713	1.614772	1.54E-06	2.53E-05		Body	UBE2G1	17
488	cg14410395	-2.24408	0.02649362	0.05664	0.061109	-1.31798	7.67E-05	0.0008681	Island			5
489	cg17868307	-2.24541	0.0265708	0.85057	0.861729	1.770123	1.57E-07	3.13E-06		Body;Body	EIF4H;EIF4H	7
490	cg21198586	-2.24667	0.02659596	0.73613	0.751955	2.055849	1.60E-09	4.50E-08		Body	TEAD1	11
491	cg19781376	-2.24431	0.02660644	0.06802	0.075055	-1.67211	6.73E-07	1.19E-05	Island	Body;Body;E	RGMA;RGMA;RGI	15
492	cg02094071	2.2467	0.02660788	0.91383	0.903683	-2.0924	8.61E-10	2.52E-08		TSS1500	LRRC69	8
493	cg16628205	-2.24681	0.02665136	0.76946	0.785254	2.227725	8.12E-11	2.82E-09		TSS1500	TFR2	7
494	cg19869091	2.24278	0.0266514	0.86818	0.861044	-1.5193	5.77E-06	8.47E-05	N_Shore			1
495	cg03060158	-2.24713	0.02667046	0.85316	0.863952	2.337279	1.12E-11	4.46E-10	S_Shore	TSS1500;TSS	IP6K2;IP6K2;IP6K2	3
496	cg22721170	2.24393	0.02672824	0.86997	0.846363	-1.92713	1.34E-08	3.23E-07				8
497	cg10668189	-2.24201	0.02679908	0.87302	0.882901	1.776873	1.41E-07	2.85E-06		Body	GPR84	12
498	cg04049225	-2.23926	0.02691873	0.8237	0.832531	-1.60936	1.66E-06	2.71E-05	S_Shore	5'UTR;Body	KIAA0182;KIAA01	16
499	cg10869730	2.23936	0.02693306	0.88269	0.867568	-1.66396	7.58E-07	1.33E-05				14
500	cg25935831	2.24202	0.02697151	0.88438	0.865182	-2.23413	7.25E-11	2.54E-09		3'UTR;3'UTR	PAN3;PAN3	13
501	cg23852646	-2.24521	0.02702983	0.91638	0.923878	3.031314	1.12E-17	1.08E-15		3'UTR	PAAF1	11
502	cg08957018	-2.23639	0.02703726	0.03958	0.04348	-1.4077	2.51E-05	0.0003192	S_Shore	TSS1500	NDUFA6	22
503	cg08559459	2.2375	0.02708175	0.88118	0.865317	-1.72801	2.95E-07	5.59E-06	S_Shelf	Body;Body	ARL13B;ARL13B	3
504	cg06428797	-2.23536	0.02712015	0.75562	0.774837	1.443942	1.57E-05	0.0002098	N_Shelf			19
505	cg20948956	2.23467	0.02718222	0.90098	0.888185	-1.48782	8.81E-06	0.0001244		Body	LTBP1	2
506	cg23024136	2.23458	0.02720965	0.12941	0.116189	1.544677	4.08E-06	6.18E-05	Island			5

507	cg25798341	2.2348	0.02724588	0.85005	0.834521	-1.68101	5.91E-07	1.06E-05		Body	TAP2	6
508	cg26409748	2.23732	0.02725009	0.88879	0.872148	-2.13792	3.93E-10	1.22E-08		3'UTR;Body	SPEF2;SPEF2	5
509	cg18581929	-2.23434	0.02727762	0.70735	0.720894	1.684057	5.66E-07	1.02E-05				7
510	cg24886710	-2.2322	0.02730335	0.76872	0.779213	1.36008	4.57E-05	0.0005464		Body	CHD5	1
511	cg00044372	-2.23416	0.02730906	0.73558	0.753715	1.733902	2.71E-07	5.16E-06		5'UTR	KIAA0513	16
512	cg18376227	2.23626	0.02736737	0.88691	0.868626	-2.25975	4.58E-11	1.65E-09	S_Shelf	5'UTR;5'UTR	ZFYVE16;ZFYVE16	5
513	cg17105689	-2.23159	0.02738596	0.02801	0.031444	-1.47436	1.05E-05	0.000146	Island	TSS1500	NAP1L4	11
514	cg13817265	-2.23182	0.02741307	0.73783	0.754968	1.589939	2.18E-06	3.48E-05		5'UTR	SOX5	12
515	cg13548664	2.23097	0.0274195	0.88385	0.873094	-1.45031	1.45E-05	0.0001945		Body	GFOD1	6
516	cg08784317	2.23022	0.02743283	0.92848	0.921393	-1.34666	5.40E-05	0.0006351		Body	RALA	7
517	cg23163722	2.2296	0.02747223	0.90853	0.895891	-1.33847	5.97E-05	0.0006955	N_Shelf	Body;Body	CGGBP1;CGGBP1	3
518	cg06331700	-2.22992	0.0274982	0.85088	0.86316	1.47143	1.10E-05	0.0001513				13
519	cg04165366	2.22926	0.02754724	0.91435	0.906014	-1.4818	9.54E-06	0.0001337		Body	ASAP1	8
520	cg03138863	2.23105	0.02756066	0.89825	0.884601	-1.84064	5.29E-08	1.15E-06		TSS200	TAS2R10	12
521	cg13890972	-2.23113	0.02759525	0.90755	0.915664	1.944761	1.01E-08	2.48E-07				12
522	cg03727333	-2.23193	0.02759916	0.81255	0.828522	2.095529	8.16E-10	2.40E-08	N_Shelf	5'UTR	SGMS1	10
523	cg14211310	-2.22986	0.02765242	0.8176	0.831348	1.867274	3.48E-08	7.80E-07		Body	COL28A1	7
524	cg14577066	2.23393	0.02766005	0.89917	0.883437	-2.63482	3.85E-14	2.23E-12		Body;3'UTR;	ZFP161;ZFP161;Z	18
525	cg00732775	2.22668	0.02767453	0.86738	0.854649	-1.34501	5.51E-05	0.0006471	N_Shore	3'UTR	EGR3	8
526	cg00359465	-2.2264	0.02769941	0.78495	0.799728	1.360336	4.56E-05	0.0005448	N_Shore	TSS1500;Bo	PRKCZ;PRKCZ	1
527	cg25558218	2.22768	0.02779045	0.89834	0.883744	-1.83836	5.48E-08	1.19E-06				5
528	cg11505533	-2.22554	0.02780532	0.82549	0.835065	1.49038	8.51E-06	0.0001206	N_Shelf			1
529	cg11337388	-2.22604	0.02782138	0.74562	0.763474	1.625684	1.31E-06	2.19E-05	S_Shore	5'UTR;TSS20	B4GALT2;B4GALT	1
530	cg10163122	-2.23254	0.02782908	0.87978	0.894885	2.857127	4.32E-16	3.32E-14		Body;Body	C6orf106;C6orf10	6
531	cg23836020	2.22547	0.0278586	0.8687	0.856159	-1.61891	1.45E-06	2.40E-05				6
532	cg15675643	-2.22328	0.02791973	0.88174	0.890385	1.376128	3.74E-05	0.0004572				2
533	cg05565282	-2.22241	0.02801056	0.04942	0.054343	-1.46084	1.26E-05	0.0001716	Island	TSS200	ZMPSTE24	1
534	cg18854392	2.22409	0.02808854	0.89765	0.878687	-1.96864	6.83E-09	1.73E-07		TSS200	SERPINI2	3
535	cg24047926	-2.22346	0.02809962	0.77997	0.792474	1.88549	2.61E-08	5.98E-07		3'UTR	SOX13	1
536	cg07482451	2.22047	0.0281071	0.93729	0.932469	-1.35599	4.81E-05	0.0005721		TSS1500	ALOX12B	17

537	cg02624809	-2.22036	0.02811402	0.10185	0.108557	-1.35403	4.93E-05	0.0005846	Island	Body	COMP	19
538	cg01709689	-2.21938	0.02816775	0.75921	0.775786	1.312276	8.22E-05	0.0009226	N_Shore			7
539	cg20143773	-2.22697	0.0281701	0.82294	0.840169	2.730671	5.69E-15	3.74E-13	N_Shore	Body;Body	GFI1B;GFI1B	9
540	cg13254809	2.22145	0.02825708	0.88749	0.877693	-1.93092	1.26E-08	3.05E-07		3'UTR	FBXO33	14
541	cg18515940	-2.21825	0.02826764	0.85179	0.861701	1.372194	3.93E-05	0.0004776				8
542	cg05193662	2.22129	0.02827209	0.92945	0.923934	-1.94179	1.06E-08	2.59E-07		Body;Body	BANP;BANP	16
543	cg17979863	-2.21791	0.02827435	0.04597	0.050312	-1.32504	7.04E-05	0.000804	Island			2
544	cg27175085	2.21824	0.0282873	0.9073	0.898974	-1.4243	2.03E-05	0.0002637	N_Shelf	TSS1500	PABPN1	14
545	cg07368317	-2.21767	0.02830213	0.03798	0.041145	-1.35471	4.89E-05	0.0005803	Island	5'UTR	PJA2	5
546	cg10218347	-2.21837	0.02830626	0.90238	0.910806	1.500347	7.45E-06	0.0001069	S_Shelf			19
547	cg17174660	2.22401	0.02831727	0.9183	0.903033	-2.56443	1.53E-13	8.11E-12		Body;3'UTR;	ATP2C1;ATP2C1;A	3
548	cg21131493	2.22034	0.02832648	0.92401	0.916739	-1.91113	1.73E-08	4.09E-07				3
549	cg02864626	2.21715	0.02834638	0.83431	0.826295	-1.37647	3.72E-05	0.0004555	S_Shelf	Body;Body	ZNF167;ZNF167	3
550	cg24975342	-2.22226	0.02835249	0.84492	0.856208	2.32777	1.33E-11	5.25E-10		Body	IL31RA	5
551	cg08125824	2.22065	0.0283737	0.88318	0.862907	-2.08943	9.06E-10	2.65E-08		Body	HECTD1	14
552	cg22950111	-2.21652	0.02838	0.8365	0.846266	1.348383	5.29E-05	0.0006226	N_Shore	Body;3'UTR	SDCCAG8;AKT3	1
553	cg14071137	-2.21591	0.02842796	0.81579	0.824116	1.362659	4.43E-05	0.0005309	S_Shore			12
554	cg23610752	2.215	0.02850116	0.89285	0.875935	-1.38853	3.20E-05	0.0003977		Body;3'UTR	GORAB;GORAB	1
555	cg13491023	2.21399	0.02857062	0.91964	0.910982	-1.38349	3.41E-05	0.0004212	S_Shelf			1
556	cg09822923	2.21923	0.02859061	0.91922	0.908869	-2.39903	3.57E-12	1.54E-10		Body	CCDC76	1
557	cg05262173	2.2146	0.02867148	0.89317	0.883208	-1.76242	1.76E-07	3.48E-06				5
558	cg04574282	2.22197	0.02874133	0.90067	0.884618	-3.48316	5.35E-22	9.89E-20		Body;Body;E	TTC8;TTC8;TTC8	14
559	cg07374465	2.21315	0.02878771	0.903	0.895858	-1.7986	1.01E-07	2.09E-06	S_Shelf	Body	ATP5F1	1
560	cg21897413	-2.21152	0.02885722	0.039	0.042514	-1.68012	5.99E-07	1.07E-05	Island	TSS200	DDX41	5
561	cg05180182	-2.21031	0.02899334	0.73082	0.74752	1.808607	8.70E-08	1.82E-06				10
562	cg06219408	-2.20864	0.02902814	0.7351	0.750739	1.591216	2.14E-06	3.43E-05				8
563	cg12158165	2.21389	0.02904083	0.91568	0.905508	-2.60142	7.43E-14	4.11E-12	N_Shore			7
564	cg20461275	2.2079	0.02907117	0.88768	0.879197	-1.56582	3.05E-06	4.74E-05		Body;Body;E	PARD3B;PARD3B;	2
565	cg01082437	-2.20848	0.02907998	0.74991	0.76039	1.695389	4.79E-07	8.73E-06	S_Shelf			21
566	cg14142743	2.21015	0.0290999	0.88463	0.866574	-2.05078	1.74E-09	4.88E-08	N_Shelf	5'UTR	NFIL3	9

567	cg03060457	-2.21309	0.02910309	0.03817	0.042053	-2.61731	5.44E-14	3.08E-12	Island	TSS1500;1st	FOXRED1;SRPR	11
568	cg16956007	2.20522	0.02916855	0.88043	0.875104	-1.31098	8.35E-05	0.0009353		Body;Body	ATP11A;ATP11A	13
569	cg11434762	2.20627	0.02917071	0.84546	0.824701	-1.52145	5.60E-06	8.25E-05		Body;Body	ROBO2;ROBO2	3
570	cg18515343	-2.20792	0.02917267	0.87148	0.881684	1.829944	6.25E-08	1.34E-06	S_Shore	1stExon	C17orf105	17
571	cg21873672	2.20529	0.02921322	0.87746	0.868298	-1.44705	1.51E-05	0.0002023				5
572	cg15615443	2.20777	0.02932681	0.92223	0.915125	-2.1969	1.40E-10	4.69E-09				10
573	cg12087627	-2.20774	0.02936675	0.83807	0.848223	2.296197	2.37E-11	8.98E-10	S_Shelf	Body	ZZEF1	17
574	cg06861037	-2.20356	0.0294278	0.75326	0.767545	1.681254	5.89E-07	1.05E-05	N_Shore			16
575	cg16175560	2.20553	0.02949776	0.82179	0.803826	-2.22265	8.89E-11	3.07E-09		Body	SEC24D	4
576	cg00723840	2.20143	0.02951809	0.89614	0.890573	-1.51696	5.96E-06	8.72E-05	Island	Body;Body	ZNF74;ZNF74	22
577	cg03853620	2.20401	0.02952711	0.88392	0.86865	-2.01743	3.05E-09	8.20E-08	N_Shelf	Body	PDK4	7
578	cg16983759	-2.1995	0.02958342	0.05057	0.054216	-1.31507	7.95E-05	0.0008955	Island	Body	C18orf22	18
579	cg08359717	-2.19913	0.02961071	0.90739	0.915173	1.315378	7.92E-05	0.0008926		5'UTR;5'UTR	ZBTB20;ZBTB20	3
580	cg09509089	2.20636	0.02970151	0.89954	0.88767	-2.96241	4.81E-17	4.27E-15		3'UTR	ZCCHC10	5
581	cg02835561	2.19797	0.02973927	0.90354	0.891026	-1.43659	1.73E-05	0.0002286		3'UTR	CNN3	1
582	cg24037795	2.201	0.02977284	0.87683	0.86663	-2.08553	9.68E-10	2.82E-08	S_Shelf	TSS1500;Bo	MIR335;MEST;ME	7
583	cg15001618	2.1967	0.02980748	0.88519	0.878859	-1.37021	4.03E-05	0.0004883	Island			18
584	cg27463953	-2.19627	0.02982038	0.05801	0.064203	-1.31754	7.71E-05	0.0008724	Island	TSS1500;TSS	ATRN;ATRN	20
585	cg13846507	-2.19663	0.02982753	0.0751	0.081114	-1.41076	2.41E-05	0.0003082	Island	5'UTR;1stEx	ST6GALNAC4;ST6	9
586	cg05554594	-2.19576	0.02988219	0.88437	0.897322	1.384494	3.37E-05	0.0004166		Body	WDR45L	17
587	cg13064897	-2.19473	0.02994216	0.82535	0.836375	1.342811	5.66E-05	0.0006628				8
588	cg09675001	2.19445	0.03001641	0.88939	0.875369	-1.48652	8.96E-06	0.0001263		Body	GBP7	1
589	cg06856275	-2.19644	0.03003568	0.87836	0.886738	1.908682	1.80E-08	4.24E-07	N_Shelf			7
590	cg00995324	-2.19728	0.03006664	0.86948	0.87854	2.142273	3.65E-10	1.14E-08		Body;Body	HIPK2;HIPK2	7
591	cg15565306	2.19317	0.03009444	0.8215	0.80475	-1.44294	1.59E-05	0.0002124	S_Shelf			7
592	cg15067350	2.19393	0.03011274	0.8471	0.831427	-1.63756	1.11E-06	1.88E-05		3'UTR;3'UTR	PCDH9;PCDH9	13
593	cg22307009	-2.19216	0.0301371	0.05398	0.059678	-1.35731	4.73E-05	0.0005639	Island	TSS1500	NAPA	19
594	cg09470958	-2.19192	0.03021793	0.07753	0.086516	-1.52628	5.25E-06	7.77E-05				6
595	cg01070095	-2.19048	0.03026417	0.68589	0.702225	1.365031	4.30E-05	0.000517	N_Shore	TSS1500	SETD1A	16
596	cg27299033	-2.19518	0.03028333	0.87942	0.890486	2.302342	2.12E-11	8.11E-10	S_Shelf	Body	RTN4RL1	17

597	cg16907527	-2.19579	0.0302851	0.83742	0.850062	2.425474	2.18E-12	9.69E-11		Body;Body;E	VEGFA;VEGFA;VE	6
598	cg18949521	2.18957	0.03033692	0.85745	0.844863	-1.37913	3.60E-05	0.0004421		TSS1500	OR5V1	6
599	cg18848012	2.18846	0.03040461	0.91492	0.905455	-1.34005	5.86E-05	0.0006836	Island	Body	SLC12A7	5
600	cg00903371	-2.18834	0.03043359	0.85571	0.864651	1.392806	3.03E-05	0.0003788				2
601	cg06592065	2.1895	0.03055497	0.85945	0.848634	-1.92368	1.42E-08	3.39E-07	Island	Body	SLC12A7	5
602	cg13135654	2.18652	0.03057567	0.90544	0.900352	-1.40952	2.45E-05	0.0003126		Body	SHANK2	11
603	cg14328669	-2.19088	0.03059396	0.07993	0.087086	-2.28263	3.03E-11	1.13E-09	Island	Body	MYLK2	20
604	cg26532905	2.18785	0.03060019	0.88486	0.871362	-1.72841	2.94E-07	5.56E-06		TSS1500	RNASEL	1
605	cg22691776	-2.18725	0.0306288	0.7504	0.769201	1.687844	5.35E-07	9.66E-06		5'UTR	NR2C2	3
606	cg11398081	2.18679	0.03068507	0.86459	0.844636	-1.7436	2.34E-07	4.52E-06		TSS1500	NPVF	7
607	cg10678718	2.18588	0.03069875	0.92998	0.922373	-1.60648	1.73E-06	2.82E-05		Body	ABI3BP	3
608	cg19289599	-2.1837	0.03080876	0.04822	0.051266	-1.46745	1.15E-05	0.0001586	Island	5'UTR;5'UTR	ISYNA1;ISYNA1;IS	19
609	cg13406762	2.18461	0.03081046	0.89588	0.886945	-1.64817	9.52E-07	1.63E-05	N_Shelf			13
610	cg19856771	2.18373	0.03098363	0.90094	0.888723	-1.91398	1.66E-08	3.92E-07		Body;Body	DGKH;DGKH	13
611	cg25595541	2.18007	0.03103501	0.86842	0.850512	-1.33897	5.94E-05	0.0006917		TSS1500;Bo	FLJ10213;PPP4R2	3
612	cg21678795	2.18052	0.03106606	0.04331	0.039886	1.512234	6.35E-06	9.23E-05	Island	TSS200	CSRP2	12
613	cg03354009	-2.18884	0.03107538	0.03545	0.040301	-3.23496	1.37E-19	1.78E-17	N_Shore	TSS1500	NUP107	12
614	cg21467203	2.18008	0.03109926	0.87938	0.872755	-1.51102	6.45E-06	9.37E-05	S_Shelf			3
615	cg10717691	-2.18338	0.03113813	0.06764	0.07658	-2.2349	7.15E-11	2.50E-09				1
616	cg16190575	2.17995	0.03114571	0.91409	0.909313	-1.60456	1.77E-06	2.89E-05	Island			11
617	cg11281377	2.18296	0.03115001	0.88406	0.866363	-2.18499	1.73E-10	5.69E-09	N_Shore	3'UTR	ZDHHC20	13
618	cg16635196	-2.17824	0.03130496	0.83977	0.857064	1.678559	6.13E-07	1.09E-05	S_Shore	5'UTR	GPR160	3
619	cg03495625	2.17643	0.03133775	0.90908	0.904039	-1.40812	2.49E-05	0.0003176	N_Shore			7
620	cg13204432	2.17528	0.03141681	0.77114	0.757154	-1.38581	3.31E-05	0.0004104		Body;Body	NRXN1;NRXN1	2
621	cg17087232	2.17628	0.03141778	0.90915	0.904316	-1.58678	2.28E-06	3.63E-05	S_Shelf	Body	MAN2C1	15
622	cg11643457	2.17534	0.03142114	0.81384	0.794361	-1.40899	2.47E-05	0.0003145		Body	PTPRQ	12
623	cg00167916	2.17693	0.03146731	0.85082	0.83995	-1.83412	5.86E-08	1.26E-06		3'UTR;Body	TAP2;TAP2	6
624	cg18128212	-2.17636	0.03148894	0.88846	0.896429	1.779768	1.35E-07	2.73E-06	N_Shelf	TSS1500	PAQR7	1
625	cg00128054	2.17425	0.03150686	0.9149	0.903332	-1.41385	2.32E-05	0.0002975	S_Shore			3
626	cg09536315	2.17491	0.03151566	0.91333	0.907046	-1.56817	2.95E-06	4.60E-05		Body	CCDC141	2

627	cg20858526	-2.17746	0.03152986	0.71656	0.731458	2.08867	9.17E-10	2.68E-08		Body	CTRC	1
628	cg07710907	2.17445	0.03158409	0.9056	0.898268	-1.65178	9.04E-07	1.56E-05	N_Shelf	Body	XKR5	8
629	cg04236924	-2.18083	0.03159267	0.87564	0.884722	2.950824	6.14E-17	5.37E-15		Body;Body;E	PDHX;PDHX;PDHX	11
630	cg07916058	2.1753	0.03161606	0.9038	0.891876	-1.89269	2.33E-08	5.38E-07		Body;Body	XPR1;XPR1	1
631	cg06982805	-2.17757	0.03185353	0.06737	0.074954	-2.98684	2.87E-17	2.64E-15	Island	TSS200	FAR1	11
632	cg06451957	2.16947	0.03190652	0.86363	0.853281	-1.49525	7.98E-06	0.0001137	N_Shore	TSS1500	IL12B	5
633	cg25398044	-2.17212	0.03194293	0.87875	0.88723	2.093988	8.38E-10	2.46E-08		Body	FLJ23834	7
634	cg02733918	-2.16665	0.03210758	0.71182	0.727999	1.447449	1.50E-05	0.0002013	N_Shelf	5'UTR;5'UTR	STAU1;STAU1;STA	20
635	cg03303433	-2.16942	0.032114	0.79636	0.807169	1.999842	4.08E-09	1.07E-07	N_Shelf			16
636	cg20902425	2.16878	0.03214039	0.93563	0.931209	-1.94252	1.04E-08	2.57E-07	Island	Body	CELSR1	22
637	cg10257407	2.1673	0.0321497	0.81293	0.795237	-1.68254	5.78E-07	1.04E-05	N_Shelf			12
638	cg03843493	2.16627	0.03216591	0.86498	0.854175	-1.52131	5.62E-06	8.26E-05		Body	SEC24D	4
639	cg00656991	2.16735	0.03218985	0.91729	0.906702	-1.79033	1.15E-07	2.35E-06		5'UTR;5'UTR	FAM135A;FAM13	6
640	cg19196918	-2.16728	0.03219148	0.04134	0.045658	-1.78171	1.31E-07	2.66E-06	Island	1stExon;5'U	ESD;ESD	13
641	cg06395634	-2.16596	0.0321958	0.7135	0.73214	1.536166	4.59E-06	6.87E-05	N_Shore	TSS1500	LDLRAP1	1
642	cg24719912	2.16725	0.03221148	0.90374	0.888048	-1.82505	6.75E-08	1.44E-06		3'UTR;3'UTR	KRR1;GLIPR1	12
643	cg20705829	2.16959	0.03223454	0.8161	0.797561	-2.33389	1.19E-11	4.73E-10	N_Shelf	Body	CBR4	4
644	cg04864755	2.16475	0.03227017	0.92726	0.919858	-1.48366	9.31E-06	0.0001308	S_Shelf	Body	BRD1	22
645	cg08512490	-2.16574	0.03228567	0.03585	0.041039	-1.71715	3.47E-07	6.49E-06	Island	Body;Body	ZFHX3;ZFHX3	16
646	cg25064274	2.16286	0.03236444	0.90205	0.890397	-1.34347	5.62E-05	0.0006581				1
647	cg25924602	2.16607	0.03240205	0.90694	0.8976	-2.06454	1.38E-09	3.93E-08		Body;5'UTR	C1QTNF7;C1QTNI	4
648	cg10998227	-2.16571	0.03241746	0.80158	0.818123	2.034769	2.28E-09	6.27E-08	N_Shore			12
649	cg26254404	2.16811	0.03243836	0.88694	0.876139	-2.56395	1.54E-13	8.18E-12	S_Shelf			2
650	cg20810398	-2.16372	0.03244589	0.02545	0.027364	-1.72234	3.22E-07	6.04E-06	Island	1stExon;1stE	EXOSC10;EXOSC1	1
651	cg20294640	2.16233	0.03246542	0.87414	0.852064	-1.49725	7.77E-06	0.0001109	S_Shelf	Body	SELT	3
652	cg00672712	-2.16623	0.03249193	0.83206	0.84261	2.320454	1.52E-11	5.95E-10	N_Shelf	3'UTR	C7orf43	7
653	cg20666019	2.1623	0.03249754	0.87393	0.857894	-1.57257	2.78E-06	4.35E-05				7
654	cg26675906	2.16547	0.03258935	0.91815	0.905731	-2.41779	2.51E-12	1.11E-10	S_Shelf	5'UTR	TAOK1	17
655	cg23421390	2.16125	0.03260651	0.90348	0.896385	-1.63781	1.11E-06	1.87E-05		3'UTR;3'UTR	SEC23B;SEC23B;S	20
656	cg04181189	-2.15996	0.03261973	0.77673	0.795382	1.413401	2.33E-05	0.000299		5'UTR;5'UTR	PRDM10;PRDM1(	11

657	cg22565623	2.16123	0.03266486	0.90498	0.893379	-1.778	1.39E-07	2.80E-06				8
658	cg23096401	2.15909	0.03267739	0.88234	0.87561	-1.38449	3.37E-05	0.0004166		Body	CREB3L2	7
659	cg23476276	2.1592	0.03272257	0.9321	0.926356	-1.52201	5.56E-06	8.19E-05				10
660	cg14597822	2.16157	0.0328412	0.90064	0.883231	-2.27625	3.40E-11	1.25E-09	N_Shelf			19
661	cg07937999	2.15684	0.032867	0.90557	0.896075	-1.41149	2.39E-05	0.0003056				14
662	cg21904719	2.1592	0.03289054	0.8509	0.838963	-1.93591	1.16E-08	2.83E-07				8
663	cg10653000	2.1591	0.03295342	0.8575	0.844582	-2.06898	1.28E-09	3.66E-08	Island	Body	GALNT9	12
664	cg04420991	2.15994	0.03297809	0.89653	0.880804	-2.29503	2.42E-11	9.16E-10	N_Shore	Body	NUP54	4
665	cg13883232	-2.15493	0.03304649	0.85142	0.860358	1.482385	9.47E-06	0.0001327	S_Shore	TSS1500	MAMDC4	9
666	cg03713923	2.15386	0.03311317	0.89642	0.885867	-1.43223	1.83E-05	0.0002404		Body	IQCH	15
667	cg26000536	-2.15551	0.03313352	0.8612	0.869703	1.811548	8.31E-08	1.75E-06	S_Shore	Body;Body	PRDM16;PRDM16	1
668	cg16662787	-2.15282	0.03320768	0.04067	0.043995	-1.46232	1.23E-05	0.0001686	Island	Body;Body;E	ATXN2L;ATXN2L;A	16
669	cg26884161	2.15306	0.03327552	0.86877	0.848176	-1.67832	6.15E-07	1.10E-05	S_Shelf			1
670	cg15999098	2.15716	0.0333344	0.88619	0.871366	-2.64481	3.16E-14	1.86E-12		Body	PLA2G4A	1
671	cg09227119	-2.15153	0.03336545	0.78886	0.802499	1.596751	1.98E-06	3.19E-05		TSS200	C20orf118	20
672	cg05887421	-2.1513	0.03337403	0.71612	0.729451	1.571425	2.82E-06	4.42E-05	S_Shore	TSS1500;TSS	ATPBD4;ATPBD4	15
673	cg15001633	2.15211	0.033386	0.8516	0.831317	-1.76232	1.76E-07	3.48E-06		3'UTR	UBR5	8
674	cg07495027	-2.15305	0.03338726	0.85601	0.864318	1.947799	9.59E-09	2.37E-07	N_Shore	Body;Body;E	EDNRB;EDNRB;ED	13
675	cg15625467	-2.14985	0.0333964	0.07215	0.079205	-1.32982	6.64E-05	0.0007641				6
676	cg20468999	-2.15291	0.03340282	0.89081	0.899389	1.958682	8.03E-09	2.01E-07	Island			18
677	cg13720278	2.15161	0.0334266	0.89033	0.883193	-1.76212	1.77E-07	3.49E-06		3'UTR	DNAJC19	3
678	cg17280398	-2.14994	0.03346335	0.79272	0.808842	1.521397	5.61E-06	8.25E-05		TSS200	PIK3CB	3
679	cg14741366	2.15348	0.03349487	0.92999	0.918774	-2.29669	2.35E-11	8.91E-10	N_Shelf	Body	DPM1	20
680	cg25486039	-2.15181	0.0335388	0.83625	0.845327	2.072631	1.21E-09	3.45E-08		TSS200;TSS2	PGC;PGC	6
681	cg11547764	2.15279	0.03354746	0.92209	0.915408	-2.28805	2.75E-11	1.03E-09		TSS1500	OR2Z1	19
682	cg18284590	-2.15033	0.03356767	0.76404	0.779877	1.854131	4.28E-08	9.45E-07		Body;Body	CLCNKB;CLCNKB	1
683	cg11755796	-2.14777	0.03356789	0.09173	0.099898	-1.33961	5.89E-05	0.0006868	Island	TSS1500	SLC24A3	20
684	cg23694389	-2.15181	0.03357577	0.87468	0.883319	2.16334	2.53E-10	8.09E-09	N_Shore	Body;Body	MKNK2;MKNK2	19
685	cg04260557	-2.15114	0.03361598	0.84196	0.852487	2.128606	4.62E-10	1.42E-08	S_Shelf	Body	VAT1L	16
686	cg02515133	2.14777	0.03363247	0.88778	0.87957	-1.50467	7.03E-06	0.0001014	Island	Body	GRM3	7

687	cg03727481	-2.15137	0.03364403	0.89063	0.898877	2.242977	6.19E-11	2.19E-09		Body	TMPRSS13	11
688	cg19753914	2.14782	0.03369346	0.87459	0.862409	-1.66576	7.39E-07	1.30E-05		5'UTR	GULP1	2
689	cg16228380	2.14795	0.0337069	0.81196	0.800886	-1.72383	3.14E-07	5.92E-06				8
690	cg19537932	2.14806	0.03371724	0.91446	0.899804	-1.77048	1.56E-07	3.11E-06		1stExon	OR6C68	12
691	cg20138604	2.14659	0.03374752	0.91483	0.910151	-1.55269	3.66E-06	5.59E-05		Body	NCAPD3	11
692	cg25043602	2.14975	0.03376089	0.76956	0.756302	-2.20878	1.14E-10	3.86E-09	N_Shelf	Body;Body	ATP11A;ATP11A	13
693	cg06294856	-2.14513	0.03378385	0.85104	0.860511	1.3411	5.78E-05	0.0006756	N_Shore	Body	ADAMTSL1	9
694	cg26992028	-2.1522	0.03383117	0.87924	0.888321	2.918915	1.20E-16	1.00E-14	Island			7
695	cg09926135	2.14586	0.03384031	0.88847	0.881825	-1.63423	1.16E-06	1.96E-05	N_Shelf			10
696	cg03114244	-2.15019	0.03385507	0.04292	0.047016	-2.5365	2.63E-13	1.35E-11	Island	5'UTR;TSS15	FEN1;C11orf10;M	11
697	cg22920404	2.14455	0.03385748	0.93103	0.925172	-1.41026	2.43E-05	0.00031		TSS1500	C9	5
698	cg14056738	2.14454	0.03387545	0.86311	0.852235	-1.45343	1.39E-05	0.0001874				2
699	cg21411962	2.14631	0.03389084	0.8237	0.802354	-1.84585	4.88E-08	1.07E-06	N_Shelf	3'UTR	KIAA0562	1
700	cg11610702	-2.1446	0.03389588	0.05822	0.062138	-1.51848	5.83E-06	8.55E-05	Island	Body	LOC349114	7
701	cg07759223	-2.14846	0.03391112	0.82688	0.843061	2.321016	1.51E-11	5.89E-10	N_Shore	Body;Body;E	ANK1;ANK1;ANK1	8
702	cg09326135	-2.1478	0.03393527	0.89358	0.900808	2.246524	5.81E-11	2.06E-09	N_Shelf	Body	EBF3	10
703	cg19796974	-2.14669	0.03394092	0.9153	0.923833	2.040414	2.08E-09	5.74E-08	S_Shore	Body	WDR88	19
704	cg02777447	2.14377	0.03400784	0.89899	0.889813	-1.62753	1.28E-06	2.14E-05		Body	CD2AP	6
705	cg16598977	-2.14161	0.03406271	0.05337	0.057542	-1.31784	7.68E-05	0.0008694	Island			10
706	cg11960784	2.1435	0.03407685	0.90506	0.891429	-1.73988	2.47E-07	4.75E-06	N_Shelf	3'UTR;3'UTR	SEH1L;SEH1L	18
707	cg17465881	-2.14606	0.0341164	0.05301	0.057124	-2.34379	9.94E-12	3.99E-10	Island	TSS1500	PER3	1
708	cg16534579	2.14253	0.03414799	0.87391	0.85902	-1.7205	3.30E-07	6.20E-06	N_Shelf	Body;Body;E	PARK2;PARK2;PAI	6
709	cg21206021	2.14872	0.03422554	0.91473	0.903912	-3.2695	6.39E-20	8.73E-18				8
710	cg25952663	-2.143	0.03426707	0.86787	0.877371	2.099338	7.65E-10	2.26E-08	N_Shelf	TSS1500	FLJ12825	12
711	cg26985447	-2.13949	0.03433222	0.5924	0.60483	1.558035	3.40E-06	5.23E-05		5'UTR;Body	KIAA0284;KIAA02	14
712	cg20791744	2.14241	0.03433777	0.89518	0.878793	-2.15297	3.03E-10	9.57E-09		3'UTR;3'UTR	NR1D2;NR1D2	3
713	cg04256065	2.14189	0.03434361	0.90986	0.894462	-2.06407	1.39E-09	3.95E-08	S_Shore	3'UTR;3'UTR	TNRC6C;TNRC6C	17
714	cg07012725	2.14374	0.03436181	0.89303	0.883982	-2.48547	6.99E-13	3.35E-11				7
715	cg05302218	-2.13914	0.03439154	0.78579	0.795824	1.632276	1.20E-06	2.01E-05	N_Shelf	Body;Body	SCARB1;SCARB1	12
716	cg02553663	-2.14249	0.03442752	0.03666	0.042132	-2.38947	4.27E-12	1.81E-10	Island	5'UTR;1stEx	SECTM1;SECTM1	17

717	cg21566278	2.14154	0.03444001	0.91467	0.903342	-2.22624	8.34E-11	2.89E-09		3'UTR;3'UTR	C13orf31;C13orf3	13
718	cg05886789	-2.1395	0.03446078	0.84741	0.858445	1.872202	3.22E-08	7.26E-07		Body	PLXDC2	10
719	cg24881993	-2.13784	0.03448777	0.7825	0.800135	1.604832	1.77E-06	2.88E-05		Body	RBMS2	12
720	cg27313007	2.13624	0.03455435	0.82032	0.810103	-1.44124	1.63E-05	0.0002167		Body;Body	RPTOR;RPTOR	17
721	cg05133323	-2.13552	0.03459905	0.73264	0.750178	1.402528	2.68E-05	0.0003387		Body	MED13L	12
722	cg00405789	2.13666	0.03460796	0.88175	0.866817	-1.65842	8.21E-07	1.43E-05		Body;Body;T	ZFAT;ZFAT;ZFATA	8
723	cg17114257	-2.13752	0.03463624	0.05385	0.059817	-1.89784	2.14E-08	4.99E-07	Island	TSS1500	FCHSD2	11
724	cg23554917	-2.13777	0.03464661	0.89207	0.900414	1.973295	6.32E-09	1.61E-07	S_Shelf	Body	UBASH3B	11
725	cg03032677	-2.13823	0.03465668	0.78959	0.80559	2.088971	9.13E-10	2.67E-08	S_Shore	5'UTR;5'UTR	CDKN1A;CDKN1A	6
726	cg00987534	-2.1348	0.03468889	0.70321	0.720679	1.477529	1.01E-05	0.0001407				7
727	cg20272287	2.13845	0.03470385	0.88438	0.875817	-2.24697	5.76E-11	2.05E-09	Island	TSS200;Body	MIR1180;B9D1	17
728	cg00975791	2.13658	0.03477225	0.90542	0.894814	-2.03663	2.21E-09	6.09E-08		Body;TSS150	SNORD114-18;SN	14
729	cg19578751	-2.13375	0.03478838	0.85033	0.860143	1.50744	6.77E-06	9.80E-05	N_Shore	Body	EGFL8	6
730	cg13568415	-2.13239	0.03483256	0.7805	0.79708	1.329596	6.66E-05	0.000766	N_Shore	Body;Body;E	LFNG;LFNG;LFNG,	7
731	cg13602547	-2.13259	0.034853	0.7493	0.767678	1.426029	1.98E-05	0.0002584		Body;Body;E	ARID1B;ARID1B;A	6
732	cg11180316	2.1332	0.03494929	0.86193	0.84469	-1.7857	1.24E-07	2.51E-06				14
733	cg24429974	-2.13081	0.03499514	0.64793	0.664657	1.408736	2.47E-05	0.0003154		TSS1500	TOMM40L	1
734	cg04043957	2.13211	0.03499682	0.93065	0.924375	-1.67977	6.02E-07	1.07E-05		TSS1500;TSS	TNNI2;TNNI2;TNN	11
735	cg08515811	-2.13177	0.03502105	0.87493	0.883957	1.669356	7.01E-07	1.23E-05	N_Shore	Body	TBC1D16	17
736	cg16765531	2.13112	0.03502893	0.82748	0.807234	-1.55698	3.45E-06	5.30E-05				7
737	cg22549437	2.13057	0.03503771	0.88591	0.874095	-1.46479	1.20E-05	0.0001637		3'UTR	RNF144B	6
738	cg03719155	-2.1313	0.03511407	0.06552	0.073434	-1.79838	1.02E-07	2.10E-06	S_Shore	TSS1500;TSS	ACP5;ACP5;ACP5;	19
739	cg26836026	2.12916	0.03512241	0.9049	0.900533	-1.37767	3.67E-05	0.0004494	N_Shore	Body	WHSC2	4
740	cg01613249	2.12952	0.03518912	0.84941	0.834392	-1.61773	1.47E-06	2.43E-05		5'UTR	SPTBN1	2
741	cg05405742	-2.13217	0.03520554	0.81562	0.830802	2.191252	1.55E-10	5.14E-09	S_Shore	TSS1500;Bo	ZBTB11;LOC1000	3
742	cg01503450	-2.12997	0.03521862	0.76005	0.772941	1.781126	1.33E-07	2.68E-06	S_Shelf			10
743	cg11309785	2.13028	0.03523397	0.90998	0.901679	-1.87904	2.89E-08	6.57E-07		Body	EXOC6B	2
744	cg07915117	-2.13167	0.03524531	0.79725	0.813778	2.184558	1.74E-10	5.73E-09				1
745	cg16419009	2.12687	0.03537054	0.17291	0.159234	1.514194	6.18E-06	9.01E-05	Island	Body	EN1	2
746	cg02183491	-2.13204	0.03540981	0.78401	0.799733	2.677788	1.64E-14	1.00E-12		3'UTR	FARP1	13

747	cg01043374	-2.13269	0.03549088	0.87072	0.880864	3.065794	5.36E-18	5.45E-16	N_Shelf	Body	PELP1	17
748	cg26945780	-2.12454	0.03550002	0.71764	0.732801	1.342728	5.67E-05	0.0006633		Body	SRPK1	6
749	cg01514487	-2.12993	0.03550093	0.87621	0.884628	2.453006	1.29E-12	5.96E-11		TSS200	MYO1A	12
750	cg22489583	-2.12457	0.03558376	0.06162	0.067412	-1.55766	3.41E-06	5.25E-05	N_Shore	TSS1500	IGLON5	19
751	cg00164894	2.1236	0.03561027	0.86793	0.860577	-1.41984	2.15E-05	0.0002776		Body	USP24	1
752	cg00967294	2.12531	0.03564185	0.9126	0.904136	-1.84681	4.81E-08	1.05E-06		Body;Body	ARHGAP26;ARHG	5
753	cg19157647	2.12651	0.03566687	0.88257	0.875108	-2.14848	3.28E-10	1.03E-08	Island	TSS200;Body	MIR1180;B9D1	17
754	cg19593600	2.12923	0.03567383	0.85557	0.83641	-2.74938	3.90E-15	2.62E-13	N_Shelf	Body;Body;B	HBS1L;HBS1L;HBS	6
755	cg18729186	2.12381	0.0356925	0.93088	0.924926	-1.66458	7.51E-07	1.32E-05		TSS1500;TSS	SNORD126;MIR12	14
756	cg10406264	2.12554	0.03573761	0.89224	0.88264	-2.11982	5.38E-10	1.63E-08		Body;Body	DIAPH3;DIAPH3	13
757	cg11368438	2.12337	0.03577545	0.91235	0.902891	-1.77229	1.52E-07	3.03E-06		Body	SR140	3
758	cg11367404	2.1263	0.03578311	0.86507	0.853663	-2.38909	4.30E-12	1.83E-10		Body	SH3RF1	4
759	cg15547672	2.12195	0.0357916	0.92706	0.922572	-1.51794	5.88E-06	8.61E-05		Body;Body	RPTOR;RPTOR	17
760	cg19725098	-2.12532	0.03583035	0.88535	0.893437	2.298675	2.27E-11	8.62E-10		5'UTR;3'UTR	ACACA;C17orf78;	17
761	cg26132900	2.12619	0.03583119	0.88984	0.879652	-2.48424	7.16E-13	3.42E-11				4
762	cg09636727	2.12338	0.03585905	0.87888	0.86012	-1.97019	6.66E-09	1.69E-07		Body	ST6GALNAC5	1
763	cg07454154	-2.12242	0.03590082	0.80137	0.819338	1.875627	3.05E-08	6.90E-07		Body	CCDC147	10
764	cg03292992	-2.11958	0.03593842	0.64508	0.660408	1.377343	3.68E-05	0.000451	S_Shore	TSS1500;TSS	CD59;CD59;CD59	11
765	cg26347359	2.11986	0.03594675	0.90857	0.894471	-1.459	1.29E-05	0.0001755		Body;Body	PHF17;PHF17	4
766	cg13983864	2.11973	0.03600887	0.8697	0.857615	-1.58362	2.38E-06	3.78E-05	S_Shelf			1
767	cg26145511	-2.11817	0.03606415	0.81276	0.822004	1.389625	3.16E-05	0.0003928	Island			20
768	cg15175577	-2.12379	0.03611719	0.06203	0.067535	-2.69613	1.14E-14	7.12E-13	Island	5'UTR;1stExo	C22orf28;C22orf2	22
769	cg16324899	2.11884	0.03612079	0.82784	0.805044	-1.66879	7.07E-07	1.24E-05				2
770	cg09779576	-2.11816	0.03616921	0.72692	0.743966	1.643002	1.03E-06	1.75E-05				15
771	cg09106984	-2.12167	0.03619816	0.90134	0.91007	2.434669	1.83E-12	8.25E-11	S_Shore	Body	GRM4	6
772	cg01703581	-2.11593	0.03622845	0.10266	0.107782	-1.31373	8.08E-05	0.0009085	Island	3'UTR	PYY	17
773	cg03611551	-2.11588	0.03631264	0.73127	0.746156	1.513172	6.27E-06	9.13E-05	S_Shelf			11
774	cg13966095	-2.11966	0.03632092	0.83175	0.847914	2.311036	1.81E-11	6.99E-10	N_Shelf			6
775	cg02596645	-2.11404	0.03643324	0.81931	0.827074	1.417073	2.22E-05	0.0002866		Body	FAT1	4
776	cg22456479	2.11571	0.03645415	0.90083	0.888824	-1.81556	7.81E-08	1.65E-06	N_Shelf			12

777	cg01323212	-2.11661	0.03645823	0.76155	0.776354	2.009088	3.50E-09	9.32E-08	S_Shelf			6
778	cg09334074	2.11333	0.03648161	0.90808	0.900694	-1.38257	3.45E-05	0.0004257	S_Shore	Body;Body;E	PPP2R5C;PPP2R5	14
779	cg18085280	2.11411	0.03651333	0.90459	0.897203	-1.62658	1.30E-06	2.17E-05	S_Shelf			1
780	cg08024887	-2.1171	0.03655725	0.05298	0.058857	-2.34602	9.54E-12	3.84E-10	N_Shore	Body	CCDC88B	11
781	cg08166758	2.12195	0.03662005	0.90955	0.890615	-3.81641	2.42E-25	7.46E-23		Body;Body	ARHGAP25;ARHG	2
782	cg03099832	2.11274	0.03663215	0.86369	0.849496	-1.62432	1.34E-06	2.23E-05		5'UTR;5'UTR	IL15;IL15	4
783	cg16672083	-2.11285	0.03663745	0.91824	0.926027	1.660468	7.98E-07	1.39E-05		3'UTR;3'UTR	SENP8;SENP8	15
784	cg27010927	2.11289	0.0366605	0.88291	0.873577	-1.72423	3.13E-07	5.89E-06		Body;Body;E	BPHL;BPHL;BPHL;	6
785	cg19839601	2.11199	0.03666075	0.88315	0.870506	-1.53683	4.55E-06	6.82E-05				4
786	cg08844849	-2.11153	0.03668068	0.73358	0.750258	1.48738	8.86E-06	0.000125	N_Shelf	TSS1500;TSS	SIGLEC10;SIGLEC1	19
787	cg03108928	-2.11395	0.0366926	0.86445	0.872702	2.014427	3.21E-09	8.59E-08		Body	IPO13	1
788	cg21838013	2.11069	0.03669297	0.70035	0.682093	-1.33438	6.28E-05	0.0007276		TSS1500	CRTAM	11
789	cg26141247	2.11043	0.03677616	0.90317	0.894851	-1.48598	9.03E-06	0.0001272	S_Shelf	Body;Body	PPP4R4;PPP4R4	14
790	cg01659632	2.11295	0.03681422	0.92406	0.912566	-2.09506	8.23E-10	2.42E-08		3'UTR;3'UTR	PLAGL1;PLAGL1;P	6
791	cg06404662	2.10939	0.03681798	0.91326	0.907687	-1.36183	4.47E-05	0.0005359	Island	Body;Body;E	KCNQ2;KCNQ2;K(	20
792	cg26694724	-2.10926	0.03688551	0.86095	0.871453	1.501205	7.37E-06	0.0001058	Island	Body;Body;E	MAD1L1;MAD1L1	7
793	cg02795976	-2.11007	0.0368969	0.89587	0.903832	1.697593	4.64E-07	8.47E-06		Body	GRK5	10
794	cg08415378	-2.11777	0.03690225	0.03509	0.039753	-3.50466	3.28E-22	6.27E-20	Island	5'UTR;1stEx	UMPS;UMPS	3
795	cg21245306	2.10948	0.03696981	0.87367	0.864671	-1.74763	2.20E-07	4.27E-06				9
796	cg23668057	-2.10665	0.03705598	0.73001	0.751155	1.35304	4.99E-05	0.0005911	N_Shore	TSS1500	SYNGR2	17
797	cg07565710	2.10741	0.0370937	0.8959	0.882583	-1.60908	1.66E-06	2.72E-05	N_Shelf	Body	ZNF235	19
798	cg08228672	-2.10685	0.03711217	0.86274	0.870299	1.534762	4.68E-06	7.00E-05		5'UTR;1stEx	GJC2;GJC2	1
799	cg05626290	2.10571	0.0371473	0.92716	0.921837	-1.37414	3.84E-05	0.0004674	N_Shelf	Body	PDCD7	15
800	cg11996741	-2.106	0.03725121	0.79886	0.815738	1.687193	5.40E-07	9.74E-06		Body	CCDC157	22
801	cg17174566	2.10709	0.03725137	0.8906	0.875362	-1.91279	1.69E-08	3.99E-07		Body	ACVR2A	2
802	cg05937392	2.1064	0.03730478	0.91782	0.908421	-1.89449	2.26E-08	5.24E-07		3'UTR	RDH14	2
803	cg26873832	2.10529	0.03733069	0.86846	0.853894	-1.72571	3.06E-07	5.77E-06	N_Shelf			2
804	cg12750031	-2.10405	0.03733777	0.84659	0.856171	1.480654	9.69E-06	0.0001355		Body	LRRC69	8
805	cg22020299	-2.10814	0.0373536	0.86009	0.870653	2.373766	5.71E-12	2.39E-10		5'UTR	MRPL14	6
806	cg20604957	2.10434	0.03736845	0.90329	0.891909	-1.61607	1.51E-06	2.49E-05	N_Shore	Body;Body	UHRF1;UHRF1	19

807	cg08109646	-2.10845	0.03745554	0.83523	0.851066	2.701235	1.03E-14	6.48E-13		5'UTR;5'UTR	ZNF683;ZNF683	1
808	cg16651262	-2.10534	0.03746267	0.89072	0.89818	2.044673	1.93E-09	5.37E-08	S_Shore	5'UTR;TSS15	BRE;RBKS;LOC10(	2
809	cg24770569	-2.10244	0.03746838	0.06011	0.064211	-1.45012	1.45E-05	0.0001949	S_Shore	TSS200	PIGL	17
810	cg12334636	2.10189	0.03749568	0.80782	0.794757	-1.39538	2.93E-05	0.0003679		1stExon	TMEM182	2
811	cg02227034	2.09992	0.03766545	0.89841	0.886176	-1.37969	3.58E-05	0.0004396	S_Shelf			10
812	cg21883291	-2.09895	0.03780085	0.84414	0.853836	1.499681	7.52E-06	0.0001078		Body;Body	CANX;CANX	5
813	cg27535648	-2.09851	0.03784812	0.05131	0.056298	-1.51722	5.94E-06	8.69E-05	Island	TSS200	TBC1D22A	22
814	cg20586051	-2.1006	0.03788769	0.89952	0.908034	2.048118	1.82E-09	5.09E-08	N_Shelf	Body;Body	PTPN18;PTPN18	2
815	cg16923137	-2.10025	0.03790547	0.91447	0.920963	2.017162	3.06E-09	8.24E-08	N_Shelf	Body	FAM171A1	10
816	cg07933563	-2.09773	0.03801923	0.89727	0.905181	1.75597	1.94E-07	3.80E-06	S_Shore			20
817	cg25900280	-2.09652	0.03812663	0.08013	0.087847	-1.75215	2.06E-07	4.01E-06	Island	Body	TBX4	17
818	cg06954855	-2.09955	0.03812734	0.79551	0.811611	2.39202	4.07E-12	1.74E-10		Body	SLAMF8	1
819	cg12377972	-2.09883	0.03817475	0.83782	0.853722	2.350435	8.79E-12	3.56E-10		5'UTR;1stEx(	IL4;IL4;IL4;IL4	5
820	cg06270074	2.09445	0.03820681	0.90907	0.898479	-1.49968	7.52E-06	0.0001078		5'UTR	FBXW7	4
821	cg10552942	-2.09745	0.03824344	0.87847	0.888176	2.216344	9.94E-11	3.40E-09	N_Shelf	Body	SILV	12
822	cg26101942	-2.09393	0.03826884	0.82497	0.842727	1.534847	4.67E-06	6.99E-05		5'UTR	ZNF395	8
823	cg00146612	2.09299	0.0382816	0.93513	0.927442	-1.35854	4.66E-05	0.000556		Body;Body;E	MYH11;MYH11;M	16
824	cg18943946	-2.0958	0.03830413	0.80709	0.821548	2.010782	3.41E-09	9.08E-08	S_Shelf	Body	ZZEF1	17
825	cg06531034	-2.09815	0.0383284	0.77899	0.794721	2.578423	1.16E-13	6.27E-12	S_Shelf			2
826	cg00898190	2.09245	0.03835584	0.71731	0.700634	-1.42047	2.13E-05	0.0002756				14
827	cg14200859	2.09415	0.0383679	0.86784	0.856528	-1.81422	7.98E-08	1.68E-06		5'UTR	RALGPS1	9
828	cg11432069	2.09544	0.03838043	0.86982	0.853662	-2.11328	6.02E-10	1.81E-08	N_Shelf	Body	MCM3AP	21
829	cg20677939	-2.09616	0.03839658	0.05108	0.055131	-2.30301	2.10E-11	8.02E-10	S_Shore	Body;Body;E	ARID1B;ARID1B;A	6
830	cg24021343	2.09388	0.03840007	0.89431	0.878237	-1.83179	6.07E-08	1.30E-06				3
831	cg27520776	2.09406	0.03845828	0.09545	0.0885	2.002546	3.90E-09	1.03E-07	S_Shore	TSS1500	ESRRG	1
832	cg09636525	-2.09439	0.03849283	0.86124	0.871623	2.151996	3.08E-10	9.73E-09	Island	Body	TADA2B	4
833	cg12981843	-2.09043	0.0385525	0.83137	0.840899	1.450234	1.45E-05	0.0001947	Island	Body;3'UTR	HAGHL;HAGHL	16
834	cg09614956	2.09129	0.03857011	0.81141	0.789743	-1.67948	6.05E-07	1.08E-05		3'UTR;3'UTR	IMPA1;IMPA1;IM	8
835	cg17748645	-2.09794	0.03860133	0.83212	0.847679	3.296759	3.50E-20	4.96E-18	N_Shore	Body;Body	KCNK17;KCNK17	6
836	cg19393323	2.09017	0.03861079	0.89357	0.88074	-1.53344	4.76E-06	7.11E-05		Body	FIGN	2

837	cg04286206	-2.09113	0.03865768	0.12939	0.139886	-1.84782	4.73E-08	1.04E-06		5'UTR;Body;	CASP8;CASP8;CAS	2
838	cg09930167	2.09594	0.03866166	0.88385	0.865642	-2.93226	9.06E-17	7.73E-15				12
839	cg04550367	2.08893	0.03872681	0.90035	0.889141	-1.54064	4.32E-06	6.50E-05		TSS1500;TSS	SNORD114-27;SN	14
840	cg18488623	-2.08703	0.03881719	0.05705	0.060538	-1.33622	6.14E-05	0.0007127	Island	TSS200;TSS2	TPD52L2;TPD52L2	20
841	cg25094081	-2.09272	0.03884209	0.90364	0.911142	2.631558	4.11E-14	2.37E-12	N_Shore	Body	NEU1	6
842	cg13442152	2.0873	0.03897613	0.89109	0.883953	-1.77165	1.53E-07	3.06E-06		3'UTR;3'UTR	DCAF6;DCAF6	1
843	cg19087888	2.08852	0.03899387	0.90038	0.882841	-2.07115	1.24E-09	3.53E-08		Body	ACER3	11
844	cg17377658	-2.08589	0.03900458	0.91743	0.92165	1.536351	4.58E-06	6.86E-05		Body	MYO1E	15
845	cg14400258	2.08429	0.03910726	0.87702	0.870508	-1.42731	1.95E-05	0.0002546	Island			18
846	cg22437723	2.08573	0.0391846	0.85291	0.843117	-1.91704	1.58E-08	3.75E-07		Body	WDR8	1
847	cg14548542	-2.0835	0.03920813	0.76296	0.775857	1.495333	7.97E-06	0.0001136	S_Shore	Body;Body	EGFL7;EGFL7	9
848	cg15895132	-2.08288	0.03923377	0.65039	0.665057	1.418738	2.18E-05	0.000281				1
849	cg26838787	-2.0833	0.03923636	0.84083	0.852085	1.518381	5.84E-06	8.56E-05	N_Shore	Body	GALNT9	12
850	cg14453641	-2.08202	0.03931474	0.04378	0.047527	-1.42103	2.11E-05	0.0002738	N_Shore	TSS1500;TSS	AGL;AGL;AGL;AGI	1
851	cg14245235	-2.08302	0.03933218	0.86623	0.873616	1.681828	5.84E-07	1.05E-05				10
852	cg24946177	2.08372	0.0393388	0.91911	0.912455	-1.84442	4.99E-08	1.09E-06				15
853	cg27104413	2.08507	0.03938456	0.87752	0.864154	-2.23762	6.81E-11	2.39E-09		3'UTR;3'UTR	SENP7;SENP7	3
854	cg25196440	-2.08519	0.03940155	0.88471	0.894355	2.305258	2.01E-11	7.72E-10	N_Shelf	5'UTR	FHL3	1
855	cg10390473	2.08201	0.03942452	0.92911	0.91662	-1.67642	6.32E-07	1.12E-05		Body	Mar-01	4
856	cg01009486	-2.08436	0.039444	0.86484	0.876237	2.224189	8.65E-11	2.99E-09	N_Shore	Body	SULT1A1	16
857	cg18423737	2.08275	0.03950032	0.91819	0.912627	-2.00775	3.58E-09	9.52E-08	N_Shore			18
858	cg16527552	-2.08591	0.03950354	0.80607	0.821219	2.721212	6.89E-15	4.47E-13	N_Shore	TSS1500	TMEM145	19
859	cg25245744	2.07951	0.03951984	0.88091	0.867052	-1.34935	5.22E-05	0.000616	S_Shelf			17
860	cg13534580	2.0831	0.03952159	0.9179	0.908227	-2.13129	4.41E-10	1.36E-08		Body;Body;E	LRRFIP1;LRRFIP1;	2
861	cg12240975	2.08052	0.03957626	0.85904	0.849329	-1.70741	4.01E-07	7.41E-06		Body;Body	KIF23;KIF23	15
862	cg16058975	2.0819	0.03965072	0.8404	0.82732	-2.17276	2.14E-10	6.94E-09				1
863	cg03075791	2.08374	0.03965517	0.92218	0.909273	-2.59433	8.53E-14	4.68E-12	S_Shelf	5'UTR	EPB41L5	2
864	cg18013066	2.07957	0.03968341	0.87483	0.863981	-1.74852	2.17E-07	4.22E-06		Body;Body	FAM190A;FAM19	4
865	cg03641740	2.07781	0.03970477	0.8899	0.877622	-1.41452	2.30E-05	0.0002952		Body;Body;E	PPP3CA;PPP3CA;I	4
866	cg04296250	-2.07987	0.03975944	0.89343	0.901857	1.986433	5.10E-09	1.32E-07	N_Shelf	Body	PXT1	6

867	cg20516763	2.07722	0.03978174	0.93296	0.929147	-1.46622	1.17E-05	0.0001609	Island	3'UTR	SETBP1	18
868	cg12077460	-2.078	0.03978201	0.72385	0.740602	1.63866	1.09E-06	1.85E-05		Body	MFHAS1	8
869	cg10279055	2.07766	0.03978276	0.89564	0.886961	-1.56527	3.07E-06	4.77E-05		3'UTR	VPS26B	11
870	cg07369094	-2.07603	0.03983756	0.87484	0.884309	1.328593	6.74E-05	0.000774		Body	FBXL17	5
871	cg18862260	2.07731	0.03984484	0.87179	0.854835	-1.63275	1.19E-06	2.00E-05		TSS200	MSTN	2
872	cg19250935	2.07774	0.03986678	0.90339	0.893247	-1.77654	1.42E-07	2.86E-06		Body	INPP5A	10
873	cg06804311	2.07589	0.03986771	0.90293	0.896962	-1.37043	4.02E-05	0.0004871	Island	Body	RNF222	17
874	cg03029752	-2.07575	0.03997745	0.70551	0.720605	1.601805	1.84E-06	2.99E-05	N_Shore	TSS1500;TSS	NLRX1;NLRX1	11
875	cg21698385	2.08108	0.04004041	0.91005	0.898315	-2.95667	5.43E-17	4.78E-15		Body;Body;E	MAP4K4;MAP4K4	2
876	cg14958956	2.07531	0.04004276	0.88367	0.870107	-1.65734	8.34E-07	1.45E-05		3'UTR	FAM178A	10
877	cg10600647	-2.0747	0.04010211	0.70606	0.721167	1.661252	7.89E-07	1.37E-05		Body;Body;E	BCL11A;BCL11A;E	2
878	cg21472198	-2.08013	0.04013971	0.86745	0.878615	2.986738	2.88E-17	2.65E-15	N_Shore	Body	LOC400927	22
879	cg16723803	-2.07247	0.04020923	0.07603	0.081432	-1.41594	2.26E-05	0.0002904	Island	1stExon;5'U	PIGO;PIGO;PIGO	9
880	cg02322969	-2.07326	0.04021175	0.90726	0.915372	1.59861	1.93E-06	3.12E-05				2
881	cg01371477	-2.0731	0.04028264	0.08358	0.091561	-1.72699	3.00E-07	5.67E-06	Island	TSS1500;Bo	ANP32E;ANP32E;	1
882	cg12560421	-2.07301	0.0403091	0.73417	0.751132	1.768052	1.62E-07	3.22E-06				7
883	cg00713925	-2.07088	0.04031415	0.04518	0.048056	-1.30501	8.97E-05	0.0009969		TSS200	C1orf85	1
884	cg02477042	2.07494	0.04034355	0.9168	0.908618	-2.26503	4.17E-11	1.51E-09	N_Shore	5'UTR	LASS3	15
885	cg24597466	2.07112	0.04036149	0.87977	0.866688	-1.47521	1.04E-05	0.0001446		TSS1500	SIPA1L2	1
886	cg23008404	2.07134	0.04038621	0.90516	0.899572	-1.58222	2.43E-06	3.85E-05	Island	TSS200;Body	CLDN18;CLDN18	3
887	cg20468325	-2.07268	0.04039456	0.91193	0.918578	1.890407	2.41E-08	5.56E-07				4
888	cg00545886	-2.06934	0.04053276	0.87606	0.885522	1.479946	9.78E-06	0.0001367	N_Shelf	3'UTR;3'UTR	BLCAP;BLCAP;BLC	20
889	cg04335815	-2.07025	0.04054034	0.81922	0.830032	1.697441	4.65E-07	8.49E-06	N_Shelf	Body	BLK	8
890	cg24132989	2.07069	0.04054623	0.8516	0.826675	-1.80562	9.11E-08	1.90E-06		3'UTR;3'UTR	C6orf162;C6orf16	6
891	cg12660383	2.06973	0.04056797	0.91643	0.906796	-1.64874	9.45E-07	1.62E-05		Body	SPATA5	4
892	cg20018396	2.07231	0.04057922	0.88528	0.870513	-2.23256	7.45E-11	2.60E-09				7
893	cg27386563	2.0695	0.04060021	0.88339	0.871113	-1.67116	6.83E-07	1.21E-05	N_Shelf	Body;Body	SPG11;SPG11	15
894	cg13119097	-2.0691	0.04060593	0.86231	0.871135	1.597776	1.95E-06	3.15E-05	S_Shelf	Body	RNF157	17
895	cg27192088	-2.06922	0.04069112	0.08506	0.091819	-1.81793	7.53E-08	1.59E-06	Island	TSS200	SLIT1	10
896	cg03147461	-2.07107	0.04071335	0.86971	0.8777	2.270123	3.80E-11	1.39E-09				1

897	cg00455523	2.06724	0.04072198	0.87843	0.870517	-1.45252	1.40E-05	0.0001894		Body;Body;3	ITIH5;ITIH5;ITIH5	10
898	cg03575041	-2.06791	0.040749	0.69026	0.707062	1.664362	7.54E-07	1.32E-05		TSS1500	LILRA4	19
899	cg17748092	-2.06801	0.04076315	0.84184	0.851171	1.717477	3.46E-07	6.46E-06		3'UTR	MYO1F	19
900	cg08885381	2.07202	0.04079885	0.87306	0.851798	-2.69847	1.09E-14	6.82E-13	S_Shelf	Body	TNPO1	5
901	cg08015503	2.06767	0.04080181	0.92661	0.919185	-1.73147	2.81E-07	5.34E-06	S_Shelf	Body;Body;E	ACSF3;ACSF3;ACS	16
902	cg03432948	2.06636	0.04084065	0.93108	0.925983	-1.53194	4.86E-06	7.24E-05				13
903	cg27600432	-2.06777	0.04087671	0.04669	0.052173	-1.92283	1.44E-08	3.44E-07	N_Shore			4
904	cg17953646	-2.06609	0.04090425	0.8869	0.894651	1.621034	1.40E-06	2.33E-05		3'UTR;3'UTR	SLC36A3;SLC36A3	5
905	cg07453857	-2.07197	0.04100316	0.89608	0.906272	3.258677	8.12E-20	1.09E-17		Body;Body;E	TNIK;TNIK;TNIK;T	3
906	cg03005062	2.06793	0.04101227	0.87771	0.86931	-2.26643	4.06E-11	1.48E-09				1
907	cg04182207	-2.06725	0.0410167	0.76933	0.784243	2.127854	4.68E-10	1.44E-08		TSS200	CLCN1	7
908	cg16256902	-2.06504	0.04101721	0.83518	0.845268	1.647871	9.57E-07	1.64E-05		Body;Body	DCTD;DCTD	4
909	cg16993936	2.06355	0.0411817	0.87775	0.868073	-1.69452	4.85E-07	8.83E-06		5'UTR;5'UTR	OPTN;OPTN;OPTN	10
910	cg13731703	2.06593	0.04120983	0.69531	0.680247	-2.28034	3.16E-11	1.17E-09		3'UTR	MOBKL2B	9
911	cg20749212	2.06413	0.0412425	0.89048	0.876583	-1.95891	8.00E-09	2.00E-07				11
912	cg09508556	-2.06313	0.04127726	0.84515	0.854225	1.819534	7.35E-08	1.56E-06		Body;1stExo	PSORS1C1;PSORS	6
913	cg26722544	2.06086	0.04131417	0.929	0.921271	-1.39748	2.86E-05	0.0003591		Body	RNF130	5
914	cg11192787	2.06012	0.04136631	0.84519	0.822996	-1.35045	5.15E-05	0.0006086				5
915	cg01578073	-2.05997	0.04137807	0.06125	0.067574	-1.34342	5.62E-05	0.0006584	Island	Body;1stExo	NARS2;NARS2;NA	11
916	cg06159980	-2.06789	0.04139894	0.81793	0.833141	3.272864	5.94E-20	8.16E-18		3'UTR	HSPB7	1
917	cg23186098	-2.06142	0.04142198	0.76187	0.777573	1.773645	1.49E-07	2.98E-06	S_Shore	Body	TNXB	6
918	cg19467313	-2.06368	0.04142393	0.05311	0.058059	-2.27296	3.61E-11	1.32E-09	N_Shore	TSS200	C3orf1	3
919	cg14860393	2.06706	0.04143179	0.87672	0.857497	-3.13152	1.30E-18	1.45E-16		3'UTR	KIF21A	12
920	cg07419975	-2.05947	0.04143279	0.93447	0.939326	1.358745	4.65E-05	0.0005548				11
921	cg03663456	-2.06324	0.0414846	0.92129	0.928145	2.313947	1.72E-11	6.66E-10				7
922	cg13039585	-2.06313	0.04156067	0.89213	0.899944	2.468468	9.66E-13	4.53E-11				5
923	cg08884029	2.06197	0.04157116	0.91399	0.903946	-2.23052	7.73E-11	2.69E-09	S_Shore	5'UTR;TSS15	MVK;MMAB;MVK	12
924	cg08989290	-2.05946	0.04158974	0.87546	0.883544	1.721463	3.26E-07	6.12E-06	Island	TSS1500	NHLRC4	16
925	cg19273101	2.05842	0.04159622	0.93754	0.934601	-1.50432	7.06E-06	0.0001018				2
926	cg18178844	-2.05852	0.04165613	0.84331	0.850612	1.665273	7.44E-07	1.30E-05		TSS200;Body	TNXB;TNXB	6

927	cg02461502	2.06086	0.04169069	0.82447	0.813048	-2.25771	4.75E-11	1.71E-09	N_Shore	3'UTR;3'UTR	CBFA2T3;CBFA2T:	16
928	cg01203579	2.05776	0.04169072	0.88749	0.876931	-1.57453	2.70E-06	4.24E-05				7
929	cg01466825	-2.0579	0.04169211	0.05413	0.058921	-1.60861	1.68E-06	2.74E-05		TSS1500;TSS	RNF5P1;RNF5;AG	6
930	cg09163021	-2.05697	0.04170231	0.77695	0.788773	1.420687	2.12E-05	0.0002749		TSS200;TSS2	RAPSN;RAPSN	11
931	cg20741844	-2.05737	0.04176079	0.05667	0.064066	-1.64747	9.62E-07	1.65E-05	Island	TSS200;TSS1	GNB2L1;SNORD9!	5
932	cg04317031	-2.05696	0.04178285	0.05838	0.065008	-1.60534	1.75E-06	2.86E-05	S_Shore	5'UTR	FAM73B	9
933	cg20572915	2.05536	0.04185239	0.92287	0.91645	-1.40196	2.70E-05	0.0003409		1stExon;1stE	C18orf1;C18orf1;	18
934	cg20731121	2.05719	0.04185357	0.83358	0.819613	-1.81645	7.71E-08	1.63E-06		Body;Body;E	PHLDB2;PHLDB2;I	3
935	cg03786424	2.05754	0.04187635	0.89243	0.885532	-1.94407	1.02E-08	2.51E-07	Island			4
936	cg11496226	2.05493	0.04194822	0.92961	0.922033	-1.52778	5.14E-06	7.63E-05		Body	TMEM132D	12
937	cg17369830	2.05849	0.04197295	0.9149	0.908451	-2.3754	5.54E-12	2.32E-10	Island	Body;Body	EIF2C2;EIF2C2	8
938	cg22509504	-2.05409	0.04198669	0.62379	0.637816	1.426221	1.98E-05	0.0002578				17
939	cg02363202	2.05412	0.04202183	0.86808	0.862715	-1.51549	6.08E-06	8.87E-05		Body	R3HDM2	12
940	cg19616654	2.05625	0.0420264	0.90722	0.897579	-1.99852	4.17E-09	1.10E-07	S_Shore			8
941	cg02944007	2.05453	0.04204516	0.91179	0.900819	-1.6601	8.02E-07	1.40E-05	N_Shore	Body	TSPAN10	17
942	cg11211690	2.05339	0.04209221	0.90273	0.890194	-1.51195	6.37E-06	9.27E-05	S_Shore	Body	CUL1	7
943	cg09856107	-2.05414	0.04217645	0.80312	0.818126	1.869607	3.36E-08	7.54E-07	N_Shelf	5'UTR	RAF1	3
944	cg16760382	-2.05204	0.04222231	0.90391	0.912639	1.50555	6.95E-06	0.0001003		Body	VKORC1L1	7
945	cg03683994	-2.05276	0.0422352	0.89163	0.898975	1.696985	4.68E-07	8.54E-06				16
946	cg00471536	2.05224	0.04225259	0.88651	0.876626	-1.62072	1.41E-06	2.34E-05				11
947	cg17441377	-2.05211	0.04226482	0.87539	0.890475	1.617198	1.48E-06	2.45E-05	N_Shore			17
948	cg01328158	-2.05307	0.04226988	0.90302	0.909703	1.843169	5.09E-08	1.11E-06	N_Shore			2
949	cg17728974	-2.05203	0.04227376	0.1095	0.117673	-1.62022	1.42E-06	2.36E-05	S_Shelf	TSS1500	LIN28B	6
950	cg08080395	-2.05667	0.04228055	0.80435	0.822249	2.689336	1.30E-14	8.09E-13		3'UTR	CILP	15
951	cg05795005	-2.05052	0.04240482	0.03913	0.04217	-1.57785	2.58E-06	4.07E-05	Island	TSS200;Body	LIN7C;BDNFOS	11
952	cg24789424	-2.05186	0.04241505	0.88482	0.892644	1.900177	2.07E-08	4.81E-07		1stExon;5'U	BDH1;BDH1	3
953	cg15138339	-2.05194	0.04242803	0.8288	0.846169	1.946324	9.82E-09	2.42E-07	Island	1stExon;Bod	COASY;COASY;CO	17
954	cg27000659	-2.05748	0.04243153	0.85595	0.873634	3.339139	1.36E-20	2.05E-18	N_Shelf	Body;Body;E	BAT3;BAT3;BAT3;	6
955	cg09070415	-2.05193	0.04243214	0.86558	0.872767	1.952868	8.83E-09	2.19E-07		TSS1500	TINAGL1	1
956	cg04843046	2.05093	0.04247561	0.88117	0.874056	-1.82997	6.25E-08	1.34E-06	S_Shore	TSS1500	C9orf4	9

957	cg08002861	-2.04985	0.04249039	0.88468	0.892659	1.622944	1.37E-06	2.27E-05		3'UTR;3'UTR	RG9MTD2;RG9M⁻	4
958	cg23321038	-2.04866	0.04249977	0.83726	0.8467	1.369179	4.08E-05	0.0004938				8
959	cg13177275	-2.04821	0.04253818	0.85127	0.861713	1.353558	4.96E-05	0.0005877		Body	NAV1	1
960	cg15018704	-2.0485	0.04265779	0.7547	0.769224	1.698487	4.58E-07	8.37E-06	Island	5'UTR;5'UTR	ADAT3;SCAMP4	19
961	cg02741329	2.04896	0.04266134	0.88541	0.873737	-1.80871	8.69E-08	1.82E-06		Body	LSAMP	3
962	cg17526958	2.04807	0.04271529	0.85546	0.842994	-1.73047	2.85E-07	5.41E-06	S_Shelf	Body	C5orf22	5
963	cg24420717	-2.04813	0.04274234	0.63562	0.650451	1.805454	9.13E-08	1.90E-06		5'UTR	GRN	17
964	cg26474127	2.04772	0.04284026	0.91236	0.903885	-1.93215	1.24E-08	2.99E-07		TSS200	PRHOXNB	13
965	cg24431602	2.04607	0.04285055	0.8899	0.882194	-1.58479	2.34E-06	3.72E-05	N_Shelf	Body	KLHL12	1
966	cg12321011	-2.04903	0.0428721	0.8912	0.898081	2.297706	2.31E-11	8.76E-10	Island	Body;Body	KCNH6;KCNH6	17
967	cg03229183	-2.04514	0.04288841	0.10475	0.113984	-1.45892	1.29E-05	0.0001756	Island	TSS200	TOP3B	22
968	cg06553792	-2.04914	0.04291376	0.78946	0.806987	2.419641	2.43E-12	1.07E-10				17
969	cg01776926	2.04873	0.04292578	0.92611	0.913769	-2.35099	8.70E-12	3.53E-10		Body	MED13L	12
970	cg11565377	-2.04631	0.0429315	0.7725	0.789662	1.820394	7.25E-08	1.54E-06		TSS1500;Bo(	MIR711;COL7A1	3
971	cg16140139	2.04418	0.04298839	0.90634	0.895241	-1.46784	1.15E-05	0.0001579		Body	LRPPRC	2
972	cg05045806	2.04322	0.04308621	0.92318	0.917877	-1.46984	1.12E-05	0.0001542	N_Shelf			3
973	cg16723362	-2.0426	0.04310657	0.05205	0.055308	-1.37211	3.93E-05	0.000478	Island	1stExon	MARS2	2
974	cg15073906	2.04671	0.04314956	0.9197	0.906142	-2.40322	3.30E-12	1.43E-10		3'UTR	RALGPS2	1
975	cg21638712	-2.04204	0.04323742	0.80778	0.819044	1.546445	3.98E-06	6.05E-05	S_Shelf	TSS1500;Bo(	HMBS;HMBS	11
976	cg24759237	-2.04595	0.04324633	0.88305	0.892537	2.450732	1.35E-12	6.21E-11		Body;Body	INPP4B;INPP4B	4
977	cg15621656	-2.042	0.04337653	0.05371	0.060199	-1.84788	4.73E-08	1.04E-06		Body	TNFAIP8	5
978	cg02649279	-2.03972	0.04340798	0.10451	0.123918	-1.39868	2.81E-05	0.0003542	Island			9
979	cg04455682	2.04242	0.04341646	0.91757	0.908657	-2.03066	2.44E-09	6.68E-08		3'UTR	ANLN	7
980	cg08046963	2.04061	0.04344343	0.92615	0.920704	-1.68459	5.61E-07	1.01E-05		Body	TTC7B	14
981	cg23598573	2.04276	0.04356155	0.90452	0.891499	-2.43778	1.73E-12	7.81E-11		Body	ZNF609	15
982	cg24488229	2.03828	0.04357561	0.84961	0.842829	-1.44972	1.46E-05	0.0001959		Body	COL16A1	1
983	cg24597705	-2.03737	0.04361284	0.05314	0.057161	-1.31785	7.68E-05	0.0008694	Island	5'UTR	FBXO6	1
984	cg04260992	2.03856	0.04362071	0.8909	0.878436	-1.61662	1.50E-06	2.47E-05		3'UTR;3'UTR	SYF2;SYF2	1
985	cg13493071	-2.03896	0.04362705	0.84747	0.857414	1.721856	3.24E-07	6.08E-06		Body	GNA12	7
986	cg04838225	2.04331	0.04362954	0.88791	0.873912	-2.73919	4.80E-15	3.18E-13	S_Shelf			13

987	cg24304338	-2.03883	0.04363453	0.67061	0.687687	1.708534	3.95E-07	7.30E-06	S_Shelf	Body	SPOCD1	1
988	cg09783236	-2.03545	0.04380657	0.03936	0.04356	-1.31324	8.12E-05	0.0009131	Island	5'UTR;1stEx	DDRGK1;DDRGK1	20
989	cg03599988	2.04088	0.04382137	0.90018	0.89078	-2.60642	6.73E-14	3.75E-12	N_Shore	5'UTR;Body;	PRKCZ;PRKCZ;PRK	1
990	cg12804176	2.03579	0.04388019	0.91325	0.908712	-1.56653	3.02E-06	4.70E-05		3'UTR	PIP4K2B	17
991	cg05924537	2.03509	0.04388517	0.92161	0.917727	-1.41417	2.31E-05	0.0002964		TSS1500	LRRC31	3
992	cg00229868	-2.03631	0.04389652	0.82691	0.836709	1.721929	3.23E-07	6.08E-06	S_Shore			16
993	cg13260916	2.0349	0.04396205	0.9271	0.922947	-1.54505	4.06E-06	6.16E-05	N_Shelf	Body;Body;E	SRRT;SRRT;SRRT;S	7
994	cg04227743	2.03465	0.04397843	0.88558	0.869795	-1.52614	5.26E-06	7.78E-05		3'UTR	KCNJ3	2
995	cg18308535	-2.03359	0.04400332	0.87303	0.880204	1.329457	6.67E-05	0.0007671	N_Shore	Body	FKBP8	19
996	cg01121603	-2.03396	0.04403282	0.7899	0.799469	1.488274	8.76E-06	0.0001237	Island	Body	SNED1	2
997	cg17148134	2.0336	0.04405354	0.91275	0.901659	-1.45028	1.45E-05	0.0001946				2
998	cg07106832	-2.0439	0.04406638	0.03405	0.041416	-4.51903	1.00E-32	1.12E-29	Island	TSS200;TSS2	SLC25A19;SLC25A	17
999	cg09638516	2.03323	0.04411262	0.88863	0.877667	-1.49972	7.51E-06	0.0001077	N_Shelf	3'UTR	ADI1	2
####	cg18065728	-2.03704	0.04413924	0.77935	0.794116	2.432353	1.91E-12	8.60E-11		3'UTR;3'UTR	CD74;CD74;CD74	5
####	cg03602698	-2.03352	0.04419303	0.91483	0.920793	1.747391	2.21E-07	4.28E-06		Body;Body;E	NRP1;NRP1;NRP1	10
####	cg15705930	2.03156	0.04422667	0.836	0.814653	-1.36448	4.33E-05	0.0005202		3'UTR	ZNF608	5
####	cg02848711	-2.03238	0.04434389	0.88959	0.896639	1.822071	7.06E-08	1.50E-06	S_Shelf			7
####	cg17213381	2.02996	0.04437327	0.9066	0.897247	-1.3221	7.30E-05	0.0008302		5'UTR;5'UTR	AGPAT1;AGPAT1	6
####	cg02068832	2.03095	0.04444025	0.88302	0.872093	-1.70878	3.93E-07	7.27E-06	S_Shore	Body	CACNA2D4	12
####	cg10809507	-2.0322	0.04450764	0.84073	0.849263	2.141497	3.70E-10	1.15E-08				12
####	cg03313700	2.03202	0.04453762	0.89431	0.886124	-2.16812	2.33E-10	7.49E-09	N_Shelf			2
####	cg11253785	-2.03041	0.04456343	0.89082	0.897382	1.857989	4.03E-08	8.93E-07	N_Shore	TSS1500;5'U	HIF3A;HIF3A;HIF3	19
####	cg26972479	-2.02853	0.04456601	0.7105	0.72644	1.429576	1.89E-05	0.000248	N_Shelf			17
####	cg24318537	-2.03325	0.04463456	0.87892	0.889822	2.688794	1.32E-14	8.17E-13	N_Shelf	3'UTR	UNC119B	12
####	cg24981181	2.02779	0.04464578	0.85896	0.848197	-1.43671	1.73E-05	0.0002284	N_Shelf	Body	GTSE1	22
####	cg08811314	-2.02782	0.04465972	0.08614	0.093571	-1.47686	1.02E-05	0.0001418	S_Shore	Body	CMIP	16
####	cg16908898	2.02883	0.04466359	0.90157	0.890617	-1.71989	3.33E-07	6.25E-06		Body	EML6	2
####	cg11010976	-2.02876	0.04466732	0.881	0.888871	1.710795	3.82E-07	7.08E-06				8
####	cg06769989	2.02836	0.04468738	0.88459	0.87448	-1.66378	7.60E-07	1.33E-05				5
####	cg00919509	-2.02774	0.0447284	0.88471	0.891173	1.612452	1.59E-06	2.61E-05	N_Shore	TSS1500;TSS	MYH14;MYH14;M	19

####	cg17306638	-2.02745	0.04473108	0.06436	0.070025	-1.552	3.69E-06	5.64E-05	Island			19
####	cg03577970	-2.03062	0.04476139	0.86733	0.877491	2.345763	9.58E-12	3.86E-10	S_Shore			10
####	cg15420153	-2.02759	0.04478204	0.06643	0.071145	-1.69809	4.60E-07	8.42E-06	Island	TSS200	SGPL1	10
####	cg03487269	2.02852	0.04482487	0.91314	0.90124	-2.00403	3.81E-09	1.01E-07	S_Shelf			4
####	cg21956815	2.02549	0.04483075	0.87043	0.855933	-1.30897	8.56E-05	0.0009559		3'UTR	LRP6	12
####	cg11952239	2.02628	0.04483911	0.88245	0.868132	-1.5199	5.72E-06	8.40E-05		Body	TSG1	6
####	cg09892459	2.0254	0.04489129	0.88468	0.869942	-1.43043	1.87E-05	0.0002456		Body;Body;E	PDE4D;PDE4D;PD	5
####	cg01629545	-2.02511	0.0448926	0.67882	0.695608	1.361399	4.50E-05	0.0005383	N_Shore	Body	SLC6A18	5
####	cg05593726	2.02741	0.04496476	0.91353	0.901999	-2.05757	1.56E-09	4.38E-08		3'UTR;3'UTR	ANKRD12;ANKRD	18
####	cg06531741	-2.02859	0.04498476	0.88395	0.891419	2.377974	5.28E-12	2.22E-10		TSS200	HTR3B	11
####	cg00670465	-2.02564	0.0451065	0.03221	0.035609	-1.96517	7.23E-09	1.82E-07	S_Shore	5'UTR;5'UTR	CCDC77;CCDC77;[illegible]	12
####	cg10226257	2.02731	0.04511821	0.86972	0.854963	-2.37724	5.36E-12	2.25E-10		Body	ODZ3	4
####	cg23707478	-2.02396	0.045137	0.78131	0.789922	1.646555	9.75E-07	1.67E-05	S_Shelf	Body	HCCA2	11
####	cg12732734	2.02357	0.04521313	0.90524	0.890272	-1.72455	3.11E-07	5.86E-06	N_Shelf	3'UTR;3'UTR	PIGX;PIGX	3
####	cg19630665	2.02162	0.04524113	0.85402	0.846003	-1.32268	7.24E-05	0.0008249				10
####	cg00078746	2.02361	0.04524654	0.91589	0.910329	-1.80633	9.01E-08	1.88E-06	Island	Body	GPR68	14
####	cg25354926	-2.02603	0.04527655	0.94075	0.948768	2.435696	1.80E-12	8.10E-11	Island	Body	CPLX1	4
####	cg21981298	-2.02246	0.04535343	0.70786	0.718869	1.778417	1.38E-07	2.78E-06				11
####	cg24953514	-2.02074	0.04537778	0.06774	0.07415	-1.42921	1.90E-05	0.0002491		Body;Body;E	GAS2;GAS2;GAS2	11
####	cg05270007	2.02182	0.04537958	0.88279	0.864653	-1.68686	5.43E-07	9.78E-06		Body;Body;3	C6orf204;C6orf20	6
####	cg22912497	-2.02044	0.04539648	0.93514	0.939457	1.397255	2.86E-05	0.0003601	Island	Body;Body	RYR1;RYR1	19
####	cg10937593	-2.02007	0.04540746	0.14211	0.147118	-1.33281	6.40E-05	0.0007397		TSS200;TSS2	SPTB;SPTB	14
####	cg02659086	-2.02145	0.04543954	0.04088	0.04396	-1.7333	2.73E-07	5.21E-06	Island	TSS200	GSTP1	11
####	cg24701694	-2.02306	0.04545269	0.86154	0.869304	2.132097	4.35E-10	1.34E-08				14
####	cg00858738	2.0223	0.04550079	0.86882	0.851572	-2.06406	1.39E-09	3.95E-08				1
####	cg19431235	-2.02353	0.04556776	0.87557	0.883621	2.505748	4.74E-13	2.33E-11		Body	DIAPH3	13
####	cg24102700	2.02096	0.04559706	0.90252	0.889928	-1.96553	7.18E-09	1.81E-07		Body;Body	C17orf85;C17orf8	17
####	cg20165604	2.01978	0.04562369	0.91717	0.91277	-1.75326	2.02E-07	3.95E-06				8
####	cg07236207	-2.01977	0.04564599	0.87836	0.885629	1.798465	1.02E-07	2.10E-06	Island	Body	KLHL17	1
####	cg04101363	2.02237	0.04568722	0.9251	0.918136	-2.50185	5.11E-13	2.50E-11		Body;Body	ZFHX3;ZFHX3	16

####	cg10203943	-2.01779	0.04573883	0.05104	0.055612	-1.54359	4.14E-06	6.27E-05	Island	Body;Body	ZRANB2;ZRANB2	1
####	cg23933289	-2.02292	0.04576265	0.80939	0.82586	2.826315	8.14E-16	6.02E-14	S_Shelf	5'UTR	FAM20B	1
####	cg27560182	2.01716	0.04576452	0.93542	0.930244	-1.45048	1.44E-05	0.0001941		Body;Body	CNKSR1;CNKSR1	1
####	cg04890266	2.01704	0.04578362	0.86461	0.85222	-1.46572	1.18E-05	0.0001619		Body	ADCY5	3
####	cg20889394	-2.01655	0.04579674	0.73687	0.751744	1.374082	3.84E-05	0.0004677	Island	3'UTR;3'UTR	C19orf6;C19orf6	19
####	cg18887759	2.01589	0.04583835	0.912	0.904953	-1.30816	8.64E-05	0.0009641	S_Shelf	Body	MED25	19
####	cg27291238	-2.01709	0.04584956	0.88992	0.897043	1.624791	1.33E-06	2.22E-05	S_Shore			15
####	cg20739984	-2.01814	0.04585401	0.85907	0.867623	1.878342	2.92E-08	6.64E-07				2
####	cg17325911	-2.01899	0.04593839	0.83886	0.849057	2.260078	4.55E-11	1.65E-09	S_Shelf	Body	XRCC5	2
####	cg23893897	-2.02071	0.04594386	0.91209	0.919522	2.698429	1.09E-14	6.82E-13		Body;Body	MAP4K5;MAP4K5	14
####	cg16286055	-2.02391	0.04595399	0.88438	0.898362	3.725998	2.01E-24	5.38E-22	N_Shelf	Body	JAZF1	7
####	cg22189725	-2.01771	0.04596094	0.06205	0.067009	-2.01282	3.29E-09	8.80E-08	Island	TSS200	ADC	1
####	cg15456821	-2.01487	0.04598203	0.71042	0.724838	1.391376	3.09E-05	0.000385		TSS200;TSS2	BEST1;BEST1	11
####	cg18227189	-2.01856	0.04601652	0.89351	0.901225	2.33319	1.21E-11	4.78E-10		Body	PTGFRN	1
####	cg26574247	-2.01503	0.04603609	0.85797	0.867801	1.554578	3.56E-06	5.46E-05	N_Shelf	3'UTR	ENPP7	17
####	cg04802221	-2.01349	0.04609982	0.84961	0.858376	1.323337	7.19E-05	0.0008191		5'UTR;1stEx	EXOC3L;EXOC3L	16
####	cg22260625	-2.01578	0.04613641	0.72332	0.738092	1.94915	9.38E-09	2.32E-07		3'UTR;3'UTR	PLXNB1;PLXNB1	3
####	cg15625324	2.01459	0.04616399	0.8875	0.869803	-1.73337	2.73E-07	5.20E-06		Body;5'UTR	MED12L;P2RY12	3
####	cg02346135	-2.01736	0.04628512	0.7933	0.80587	2.664816	2.13E-14	1.28E-12	Island	Body	RNF126	19
####	cg22870295	-2.01219	0.04630657	0.06562	0.071233	-1.48016	9.75E-06	0.0001363		Body	ATXN7L1	7
####	cg24375748	2.01195	0.04632002	0.87279	0.855746	-1.45395	1.38E-05	0.0001862		Body	C4orf22	4
####	cg19561607	-2.01017	0.04647579	0.87548	0.884195	1.373551	3.86E-05	0.0004705	S_Shore	TSS1500	BCAT1	12
####	cg15836599	2.01063	0.04650753	0.64111	0.62956	-1.5586	3.37E-06	5.19E-05	S_Shelf	Body;Body	ZNF37A;ZNF37A	10
####	cg10216498	-2.01111	0.04652705	0.02613	0.02845	-1.71363	3.66E-07	6.81E-06	Island	Body	TH1L	20
####	cg00993484	-2.01046	0.04656992	0.85889	0.867261	1.656001	8.51E-07	1.47E-05	S_Shelf			2
####	cg25457064	-2.0098	0.04661178	0.84219	0.850455	1.592273	2.11E-06	3.38E-05	Island	Body	ZFR2	19
####	cg21712831	-2.01061	0.04665561	0.05096	0.056944	-1.87816	2.93E-08	6.65E-07				8
####	cg12718874	2.01028	0.04666619	0.92259	0.913662	-1.82253	7.01E-08	1.49E-06	N_Shelf			4
####	cg14188401	-2.01064	0.04671535	0.68792	0.702738	2.014142	3.22E-09	8.62E-08	S_Shelf			3
####	cg05026102	-2.00985	0.04675762	0.04925	0.053799	-1.92203	1.46E-08	3.48E-07	Island	5'UTR;1stEx	ALOX5;ALOX5	10

####	cg12550312	2.00901	0.04677343	0.83249	0.825389	-1.76041	1.82E-07	3.57E-06	N_Shelf	Body;Body	RBM38;RBM38	20
####	cg11943753	-2.00746	0.04678076	0.64331	0.656451	1.405246	2.59E-05	0.0003283	S_Shore	TSS1500;TSS	ANAPC7;ANAPC7	12
####	cg08205396	-2.00816	0.04679095	0.88044	0.887554	1.597895	1.95E-06	3.14E-05	N_Shore			8
####	cg16792312	-2.01267	0.04680196	0.83908	0.851274	2.707019	9.16E-15	5.82E-13	N_Shore	Body;TSS15(	LOC100128788;SI	16
####	cg11757651	2.00974	0.04680723	0.90471	0.892607	-2.00205	3.94E-09	1.04E-07				6
####	cg14993813	-2.00682	0.04686373	0.08073	0.088902	-1.43503	1.76E-05	0.0002328	N_Shore	TSS200	NSUN4	1
####	cg17505852	-2.00994	0.04689027	0.84419	0.858148	2.232531	7.46E-11	2.60E-09	S_Shelf	Body;Body;E	NF2;NF2;NF2;NF2	22
####	cg02935562	-2.0068	0.04695561	0.74456	0.757269	1.637961	1.10E-06	1.87E-05		Body	IQCK	16
####	cg02555274	-2.00767	0.04696347	0.89434	0.901663	1.858649	3.99E-08	8.85E-07	S_Shore	Body	C3orf39	3
####	cg06525193	-2.00886	0.04699132	0.88583	0.894116	2.19716	1.40E-10	4.67E-09				2
####	cg24001728	-2.00543	0.04701675	0.76074	0.777057	1.442854	1.59E-05	0.0002126	N_Shelf	Body	VWCE	11
####	cg05357717	-2.00533	0.0470297	0.78824	0.797846	1.447947	1.49E-05	0.0002001	N_Shore	Body;Body	RTEL1;RTEL1	20
####	cg21080633	2.0067	0.04704853	0.74741	0.732669	-1.8152	7.86E-08	1.66E-06	Island	Body	SFRS8	12
####	cg03688640	-2.00464	0.04706775	0.09158	0.098273	-1.36211	4.46E-05	0.0005342	Island	5'UTR;5'UTR	ACLY;ACLY	17
####	cg04406620	-2.00381	0.04716252	0.76196	0.779069	1.372293	3.93E-05	0.0004771		3'UTR	CYB5B	16
####	cg22725830	-2.00454	0.04717726	0.70134	0.717253	1.585257	2.33E-06	3.70E-05				13
####	cg16128296	2.00664	0.04719773	0.93428	0.925145	-2.12531	4.89E-10	1.50E-08	S_Shelf	Body	ATP9B	18
####	cg21781546	2.00348	0.04724592	0.83315	0.82472	-1.48348	9.33E-06	0.000131	S_Shore	Body;Body	BCS1L;BCS1L	2
####	cg15968925	2.00825	0.0473108	0.89657	0.884465	-2.7905	1.69E-15	1.20E-13		Body;Body	MIR548H3;C6orf1	6
####	cg17966884	2.00785	0.04742364	0.87824	0.861106	-2.96971	4.12E-17	3.69E-15				4
####	cg26822602	2.00095	0.04745882	0.88703	0.87638	-1.33714	6.07E-05	0.0007056		TSS1500	VN1R5	1
####	cg25424525	-2.00321	0.04752461	0.88005	0.889577	2.0254	2.67E-09	7.25E-08		5'UTR;5'UTR	OTUD4;OTUD4;O	4
####	cg16720005	-2.0016	0.04754818	0.75236	0.768085	1.69833	4.59E-07	8.39E-06	N_Shore			15
####	cg05533329	-2.00197	0.04757266	0.8682	0.876418	1.838481	5.47E-08	1.18E-06	N_Shelf	Body	CLCN1	7
####	cg16245495	1.99996	0.04759139	0.91534	0.90597	-1.39514	2.94E-05	0.0003689	N_Shelf			2
####	cg21942815	2.00159	0.04766473	0.92044	0.913713	-1.94755	9.63E-09	2.38E-07		3'UTR;3'UTR	CEPT1;CEPT1	1
####	cg00603110	-1.99953	0.0476863	0.67527	0.688519	1.503461	7.15E-06	0.0001029				6
####	cg03405593	-2.00238	0.04779275	0.89179	0.899298	2.417926	2.51E-12	1.11E-10	Island	3'UTR	MLLT1	19
####	cg02386859	2.00016	0.04780804	0.0762	0.069573	1.918489	1.54E-08	3.67E-07	S_Shore	Body	CPE	4
####	cg08876470	-1.99771	0.04785758	0.05914	0.064124	-1.43843	1.69E-05	0.0002238	N_Shore	Body;Body	GCDH;GCDH	19

####	cg19992629	2.00025	0.04791502	0.85188	0.836516	-2.17064	2.23E-10	7.19E-09		TSS1500	ADAM21P1	14
####	cg02330264	-1.9968	0.04792656	0.10988	0.116671	-1.36722	4.18E-05	0.0005046	N_Shore	Body;Body	KCNK10;KCNK10	14
####	cg01702210	-1.99722	0.04795087	0.69653	0.709947	1.528263	5.11E-06	7.59E-05		TSS1500	SLC35F4	14
####	cg04177456	-1.99859	0.04799	0.88407	0.892217	1.940575	1.08E-08	2.64E-07	Island	Body;Body;E	PTPRN2;PTPRN2;I	7
####	cg02910194	1.99996	0.04806773	0.86533	0.846879	-2.44092	1.63E-12	7.39E-11		Body	NLGN1	3
####	cg01586506	1.99501	0.04812297	0.9391	0.932945	-1.36491	4.30E-05	0.0005177	Island	Body	SOX10	22
####	cg24685167	-1.99509	0.04814128	0.90308	0.909591	1.426964	1.96E-05	0.0002556	N_Shore	5'UTR;3'UTR	GPSM3;NOTCH4	6
####	cg02167732	-1.9945	0.04817446	0.03197	0.034904	-1.35538	4.85E-05	0.0005761	Island	TSS1500;TSS	GTF2A1;GTF2A1	14
####	cg23302558	1.99772	0.04821754	0.89917	0.886792	-2.2253	8.48E-11	2.94E-09		Body;Body	SSFA2;SSFA2	2
####	cg04737350	1.99514	0.04824933	0.88249	0.865006	-1.67963	6.04E-07	1.08E-05		Body;Body;E	PPP1R12A;PPP1R	12
####	cg14025883	-1.9939	0.04826672	0.83197	0.841328	1.413964	2.31E-05	0.0002971	N_Shore	5'UTR	C9orf46	9
####	cg27169610	-1.99673	0.04831994	0.91334	0.919619	2.210556	1.10E-10	3.75E-09		5'UTR;5'UTR	HIVEP3;HIVEP3	1
####	cg16568921	-1.99508	0.04832497	0.88355	0.892938	1.828521	6.39E-08	1.37E-06	S_Shore	Body	DENND3	8
####	cg07200122	1.99293	0.04834102	0.92637	0.920319	-1.33816	6.00E-05	0.0006979	N_Shelf			2
####	cg23564535	-1.99292	0.04841734	0.82469	0.834356	1.507882	6.73E-06	9.74E-05				11
####	cg15793456	1.99469	0.04843613	0.86811	0.848648	-1.97497	6.15E-09	1.57E-07		Body;Body	NCKAP1;NCKAP1	2
####	cg22871610	1.99333	0.04844156	0.86845	0.85078	-1.66229	7.77E-07	1.36E-05		3'UTR	NMU	4
####	cg00773205	-1.99169	0.0484887	0.05094	0.054363	-1.35962	4.60E-05	0.0005493	N_Shore	TSS1500;Bo	TECPR2;CINP	14
####	cg25467855	-1.99154	0.04855813	0.03429	0.038668	-1.48144	9.59E-06	0.0001342	Island	TSS200	LNPEP	5
####	cg01458801	1.99098	0.04857048	0.9016	0.896686	-1.36653	4.22E-05	0.0005086		3'UTR	IRS2	13
####	cg03678722	1.99396	0.0486657	0.88885	0.874249	-2.2963	2.37E-11	8.97E-10		3'UTR;3'UTR	AGFG1;AGFG1;AG	2
####	cg10769615	-1.99535	0.0486997	0.90225	0.909203	2.733204	5.41E-15	3.56E-13	S_Shore	Body	HDAC4	2
####	cg17767931	1.99007	0.04872411	0.87357	0.859245	-1.48685	8.92E-06	0.0001258		5'UTR;Body;	SLC25A26;SLC25A	3
####	cg24803261	1.99274	0.04872491	0.91007	0.903578	-2.1305	4.47E-10	1.38E-08	Island	Body	LOC619207	10
####	cg25861660	1.99142	0.04874732	0.92114	0.912353	-1.86279	3.74E-08	8.33E-07		Body	JAZF1	7
####	cg26571535	1.99205	0.04878965	0.88281	0.86581	-2.10431	7.02E-10	2.09E-08		Body	SNX27	1
####	cg23641267	-1.99178	0.04882804	0.77867	0.792576	2.121284	5.24E-10	1.60E-08	N_Shore	Body;TSS15(	LPXN;LPXN	11
####	cg09514055	-1.9904	0.04893799	0.0337	0.037553	-2.02878	2.52E-09	6.87E-08	Island	5'UTR	HYI	1
####	cg12602653	1.99295	0.04902256	0.88075	0.866907	-2.8745	3.01E-16	2.38E-14		TSS200	MEP1B	18
####	cg05248960	-1.98712	0.04907738	0.82602	0.836153	1.537518	4.50E-06	6.76E-05	S_Shelf	3'UTR;3'UTR	C16orf79;MLST8	16

####	cg23502203	1.98666	0.04919089	0.94962	0.944533	-1.67172	6.77E-07	1.20E-05	Island	Body	RASAL1	12
####	cg17054006	-1.98524	0.04920087	0.84122	0.849847	1.337371	6.05E-05	0.0007038		Body;Body	PDE1A;PDE1A	2
####	cg00865429	-1.98559	0.04927225	0.68884	0.704035	1.587401	2.26E-06	3.60E-05		Body	CLNK	4
####	cg19810092	1.98529	0.04927312	0.91008	0.902156	-1.51405	6.20E-06	9.03E-05		Body;Body;3	LYRM2;LYRM2;AN	6
####	cg03539267	1.98452	0.04930455	0.67834	0.659406	-1.39179	3.07E-05	0.0003832				10
####	cg05740575	1.98508	0.04931466	0.90928	0.904091	-1.55388	3.60E-06	5.51E-05	S_Shelf	Body	CTHRC1	8
####	cg05837753	1.98463	0.0493894	0.88985	0.878731	-1.60759	1.70E-06	2.78E-05		3'UTR	RIMKLB	12
####	cg14221831	-1.98645	0.04939507	0.88782	0.896171	2.058351	1.53E-09	4.33E-08	N_Shelf	1stExon;5'U	CDH16;CDH16	16
####	cg20890243	1.98417	0.04940778	0.89465	0.887718	-1.53335	4.77E-06	7.12E-05				7
####	cg11217666	-1.98291	0.0494753	0.79446	0.804071	1.365677	4.26E-05	0.0005133		Body	EPHB1	3
####	cg03311134	1.9888	0.049488	0.94756	0.940236	-2.8781	2.80E-16	2.21E-14	Island			10
####	cg15326755	1.98371	0.04949323	0.90686	0.90085	-1.60695	1.72E-06	2.80E-05	N_Shore	TSS1500;TSS	WSB1;WSB1	17
####	cg19386714	1.9845	0.04949565	0.86332	0.845372	-1.80489	9.21E-08	1.92E-06		3'UTR	C18orf55	18
####	cg22628851	1.98313	0.04951158	0.89911	0.886777	-1.50409	7.09E-06	0.0001021	S_Shore			8
####	cg09021393	1.98808	0.04952609	0.86802	0.849091	-2.77002	2.57E-15	1.78E-13		TSS1500;Bo	MIR591;SLC25A1	7
####	cg24878990	1.98534	0.0495482	0.87903	0.863347	-2.11798	5.55E-10	1.68E-08		3'UTR	SMURF2	17
####	cg20640749	1.98305	0.04955172	0.74205	0.732158	-1.57263	2.78E-06	4.35E-05		3'UTR	GLP1R	6
####	cg17812253	1.98204	0.04956819	0.92728	0.921729	-1.35292	5.00E-05	0.0005919	N_Shelf			1
####	cg14346872	-1.98247	0.04957885	0.8795	0.885634	1.488181	8.77E-06	0.0001238	S_Shelf	5'UTR;5'UTR	AP4S1;AP4S1	14
####	cg05435224	1.987	0.04961182	0.88911	0.871546	-2.68449	1.44E-14	8.84E-13				18
####	cg00796611	-1.98696	0.04962162	0.92295	0.92964	2.695092	1.16E-14	7.26E-13		Body;Body;E	SRGAP2;SRGAP2;	1
####	cg01415995	1.98178	0.04963348	0.92924	0.924032	-1.43388	1.79E-05	0.000236	Island	Body	GAL3ST3	11
####	cg18586212	-1.98503	0.04965974	0.81959	0.835533	2.284696	2.92E-11	1.09E-09		Body;Body;E	PPP2R2B;PPP2R2	5
####	cg06126864	1.98454	0.04968386	0.94992	0.944844	-2.21594	1.00E-10	3.42E-09				13
####	cg11643099	-1.98059	0.04971532	0.81706	0.826202	1.312004	8.25E-05	0.0009253	N_Shelf	TSS1500	GRRP1	1
####	cg03683171	-1.98334	0.04972568	0.02508	0.028394	-2.01477	3.19E-09	8.54E-08	N_Shore	Body	PFKFB3	10
####	cg21024835	1.98517	0.04975466	0.93165	0.923967	-2.53307	2.80E-13	1.43E-11	S_Shore	Body;Body;E	PTPRN2;PTPRN2;	7
####	cg08226047	1.98033	0.04977628	0.61999	0.600179	-1.38469	3.36E-05	0.0004156	N_Shelf			21
####	cg12343380	1.98142	0.04985974	0.86328	0.850761	-1.83641	5.65E-08	1.22E-06				13
####	cg01752978	-1.9794	0.04987957	0.84178	0.850422	1.377806	3.66E-05	0.0004488		Body;3'UTR;	ACO2;POLR3H;PC	22

####	cg20611230	1.97994	0.04988052	0.89227	0.879019	-1.51838	5.84E-06	8.56E-05		Body	E2F3	6
####	cg13474488	-1.97955	0.04988362	0.87481	0.881847	1.427214	1.95E-05	0.0002548				9
####	cg11669998	-1.97942	0.04993259	0.88187	0.888365	1.504263	7.07E-06	0.0001019		Body;Body	CRTC1;CRTC1	19
####	cg14623805	-1.97968	0.04997116	0.05016	0.053004	-1.65146	9.08E-07	1.56E-05	Island	5'UTR	CCAR1	10
####	cg23123362	-1.97868	0.04998803	0.08235	0.087044	-1.43964	1.66E-05	0.0002206	N_Shore	TSS200	THG1L	5
