# Supplementary material for: Increased DNA methylation variability in rheumatoid arthritis-discordant monozygotic twins
Source: Genome Med. 2018 Sep 4;10:64. doi: 10.1186/s13073-018-0575-9 (PMC6122744; doi:10.1186/s13073-018-0575-9)
Supplement: Supplementary file 3 — Table S2. Pathways enriched in differentially variable positions identified in RA-discordant twins. Pathway analysis was performed using the gometh function in the MissMethyl package. Pathways are ranked by p value (p < 0.05). (PDF 112 KB) (PDF 109 kb) [file 13073_2018_575_MOESM3_ESM.pdf]

Additional file 3: Table S2

Pathways enriched in DVPs identified in RA discordant twins, which were hypervariable in RA co-twins

	Term	Ontology	genes in term	genes with DVP	P-val
GO:0033387	putrescine biosynthetic process from ornithine	BP	3	3	0.00011721
GO:0045176	apical protein localization	BP	10	5	0.00029716
GO:0080135	regulation of cellular response to stress	BP	579	52	0.00033707
GO:0044003	modification by symbiont of host morphology or physiology	BP	43	9	0.00050695
GO:1990452	Parkin-FBXW7-Cul1 ubiquitin ligase complex	CC	3	3	0.000537
GO:0019236	response to pheromone	BP	7	3	0.00071028
GO:0006643	membrane lipid metabolic process	BP	184	20	0.00096098
GO:0044428	nuclear part	CC	4007	247	0.00144151
GO:0006664	glycolipid metabolic process	BP	105	13	0.00145173
GO:1903509	liposaccharide metabolic process	BP	106	13	0.00154319
GO:0009446	putrescine biosynthetic process	BP	5	3	0.00156382
GO:0080134	regulation of response to stress	BP	1243	87	0.0016537
GO:0009987	cellular process	BP	14229	733	0.00166822
GO:0006497	protein lipidation	BP	137	15	0.00195576
GO:0042506	tyrosine phosphorylation of Stat5 protein	BP	22	5	0.00203784
GO:0044068	modulation by symbiont of host cellular process	BP	25	6	0.0024217
GO:0009445	putrescine metabolic process	BP	6	3	0.00247902
GO:0023052	signaling	BP	5753	338	0.0024878
GO:1902269	positive regulation of polyamine transmembrane transport	BP	2	2	0.00253393
GO:0046930	pore complex	CC	17	4	0.00264507
GO:0032185	septin cytoskeleton organization	BP	2	2	0.00276487
GO:0044700	single organism signaling	BP	5746	337	0.00280267
GO:1900034	regulation of cellular response to heat	BP	78	11	0.00281313
GO:0052811	1-phosphatidylinositol-3-phosphate 4-kinase activity	MF	5	3	0.00288787
GO:0006591	ornithine metabolic process	BP	7	3	0.00289872
GO:0051716	cellular response to stimulus	BP	6394	364	0.00314538

GO:0008105	asymmetric protein localization	BP	17	5	0.00333703
GO:0031290	retinal ganglion cell axon guidance	BP	21	6	0.00334272
GO:0046467	membrane lipid biosynthetic process	BP	120	14	0.00335609
GO:0007154	cell communication	BP	5772	337	0.00344003
GO:0042158	lipoprotein biosynthetic process	BP	146	15	0.00345406
GO:0044764	multi-organism cellular process	BP	909	64	0.00349647
GO:0032872	regulation of stress-activated MAPK cascade	BP	193	21	0.00359347
GO:0006672	ceramide metabolic process	BP	74	10	0.00367265
GO:0008654	phospholipid biosynthetic process	BP	261	25	0.00371148
GO:0070302	regulation of stress-activated protein kinase signaling cascade	BP	194	21	0.00393345
GO:0006020	inositol metabolic process	BP	10	3	0.00395012
GO:0043234	protein complex	CC	2867	179	0.00401638
GO:0052745	inositol phosphate phosphatase activity	MF	18	5	0.00415368
GO:0035692	macrophage migration inhibitory factor receptor complex	CC	2	2	0.0041926
GO:0019048	modulation by virus of host morphology or physiology	BP	35	7	0.00425165
GO:1990630	IRE1-RACK1-PP2A complex	CC	2	2	0.00454601
GO:0016032	viral process	BP	903	63	0.0046611
GO:0044403	symbiosis, encompassing mutualism through parasitism	BP	942	65	0.00477655
GO:0008610	lipid biosynthetic process	BP	628	47	0.00478551
GO:0016607	nuclear speck	CC	346	31	0.00489804
GO:0044419	interspecies interaction between organisms	BP	943	65	0.00498939
GO:0038189	neuropilin signaling pathway	BP	2	2	0.00506799
GO:0038190	VEGF-activated neuropilin signaling pathway	BP	2	2	0.00506799
GO:1902336	positive regulation of retinal ganglion cell axon guidance	BP	2	2	0.00506799
GO:0030134	ER to Golgi transport vesicle	CC	72	10	0.00518206
GO:1901890	positive regulation of cell junction assembly	BP	29	6	0.00522658
GO:0015629	actin cytoskeleton	CC	430	39	0.00537396
GO:0031981	nuclear lumen	CC	3670	224	0.00546143
GO:1904071	presynaptic active zone assembly	BP	2	2	0.0056009
GO:0005654	nucleoplasm	CC	3120	195	0.00577945

GO:0016308	1-phosphatidylinositol-4-phosphate 5-kinase activity	MF	7	3	0.00595276
GO:0046292	formaldehyde metabolic process	BP	3	2	0.00603267
GO:0086023	adrenergic receptor signaling pathway involved in heart process	BP	6	3	0.00645875
GO:0002526	acute inflammatory response	BP	129	12	0.00666124
GO:2001201	regulation of transforming growth factor-beta secretion	BP	3	2	0.00668464
GO:0039519	modulation by virus of host autophagy	BP	2	2	0.00689456
GO:0051894	positive regulation of focal adhesion assembly	BP	23	5	0.00714018
GO:0032559	adenyl ribonucleotide binding	MF	1434	101	0.00716579
GO:0051403	stress-activated MAPK cascade	BP	237	23	0.00721209
GO:1901897	regulation of relaxation of cardiac muscle	BP	7	3	0.00729083
GO:0042522	regulation of tyrosine phosphorylation of Stat5 protein	BP	19	4	0.00747008
GO:0008309	double-stranded DNA exodeoxyribonuclease activity	MF	3	2	0.00762377
GO:0006021	inositol biosynthetic process	BP	4	2	0.00764476
GO:0046966	thyroid hormone receptor binding	MF	26	5	0.00764788
GO:0045017	glycerolipid biosynthetic process	BP	246	23	0.00766656
GO:0019054	modulation by virus of host process	BP	22	5	0.00768138
GO:0007289	spermatid nucleus differentiation	BP	18	4	0.00773307
GO:0043084	penile erection	BP	9	3	0.00824165
GO:0042978	ornithine decarboxylase activator activity	MF	3	2	0.00827507
GO:0035745	T-helper 2 cell cytokine production	BP	9	3	0.00835817
GO:0090501	RNA phosphodiester bond hydrolysis	BP	126	13	0.00836227
GO:0030554	adenyl nucleotide binding	MF	1445	101	0.00844092
GO:0007165	signal transduction	BP	5267	301	0.00905068
GO:0033078	extrathymic T cell differentiation	BP	3	2	0.00907498
GO:0030224	monocyte differentiation	BP	32	6	0.00907855
GO:1903131	mononuclear cell differentiation	BP	32	6	0.00907855
GO:0060488	orthogonal dichotomous subdivision of terminal units involved in lung branchin	BP	2	2	0.00909651
GO:0060489	planar dichotomous subdivision of terminal units involved in lung branching mc	BP	2	2	0.00909651
GO:0060490	lateral sprouting involved in lung morphogenesis	BP	2	2	0.00909651
GO:0071407	cellular response to organic cyclic compound	BP	521	42	0.00931679

GO:0016307	phosphatidylinositol phosphate kinase activity	MF	17	4	0.00938723
GO:0030073	insulin secretion	BP	193	20	0.00949439
GO:0046474	glycerophospholipid biosynthetic process	BP	221	21	0.00953309
GO:0006379	mRNA cleavage	BP	24	5	0.00967791
GO:0031098	stress-activated protein kinase signaling cascade	BP	243	23	0.00969233
GO:0046328	regulation of JNK cascade	BP	157	17	0.00973626
GO:0002474	antigen processing and presentation of peptide antigen via MHC class I	BP	88	10	0.0097555
GO:1901898	negative regulation of relaxation of cardiac muscle	BP	2	2	0.00984753
GO:0016309	1-phosphatidylinositol-5-phosphate 4-kinase activity	MF	3	2	0.01002867
GO:0005579	membrane attack complex	CC	7	2	0.01049544
GO:0012507	ER to Golgi transport vesicle membrane	CC	55	8	0.01050409
GO:1904430	negative regulation of t-circle formation	BP	3	2	0.01056236
GO:0097484	dendrite extension	BP	27	6	0.01071722
GO:0051117	ATPase binding	MF	75	9	0.0108841
GO:1903859	regulation of dendrite extension	BP	19	5	0.01125674
GO:0050795	regulation of behavior	BP	63	8	0.01137596
GO:0001954	positive regulation of cell-matrix adhesion	BP	45	7	0.01141635
GO:0052659	inositol-1,3,4,5-tetrakisphosphate 5-phosphatase activity	MF	8	3	0.01149935
GO:0050896	response to stimulus	BP	7795	423	0.01152445
GO:0009266	response to temperature stimulus	BP	217	20	0.01162829
GO:1903393	positive regulation of adherens junction organization	BP	25	5	0.01169776
GO:0006637	acyl-CoA metabolic process	BP	93	10	0.01176336
GO:0035383	thioester metabolic process	BP	93	10	0.01176336
GO:0006626	protein targeting to mitochondrion	BP	142	14	0.0118391
GO:0001866	NK T cell proliferation	BP	5	2	0.01184422
GO:0001952	regulation of cell-matrix adhesion	BP	93	12	0.01184572
GO:0009247	glycolipid biosynthetic process	BP	54	7	0.01188496
GO:0006931	substrate-dependent cell migration, cell attachment to substrate	BP	4	2	0.01194239
GO:0033183	negative regulation of histone ubiquitination	BP	3	2	0.01206369
GO:1901314	regulation of histone H2A K63-linked ubiquitination	BP	3	2	0.01206369

GO:1901315	negative regulation of histone H2A K63-linked ubiquitination	BP	3	2	0.01206369
GO:0043932	ossification involved in bone remodeling	BP	4	2	0.01208474
GO:0006665	sphingolipid metabolic process	BP	135	14	0.0120974
GO:0006198	cAMP catabolic process	BP	19	5	0.01213128
GO:0030127	COPII vesicle coat	CC	11	3	0.01219433
GO:0044446	intracellular organelle part	CC	8183	446	0.012221
GO:0030149	sphingolipid catabolic process	BP	20	4	0.01251657
GO:0035743	CD4-positive, alpha-beta T cell cytokine production	BP	11	3	0.01259614
GO:0030072	peptide hormone secretion	BP	229	22	0.0129202
GO:0035337	fatty-acyl-CoA metabolic process	BP	49	7	0.01295512
GO:0003824	catalytic activity	MF	5403	300	0.013026
GO:0071333	cellular response to glucose stimulus	BP	111	13	0.01305485
GO:0030150	protein import into mitochondrial matrix	BP	14	3	0.01305499
GO:1990709	presynaptic active zone organization	BP	3	2	0.0131097
GO:1901681	sulfur compound binding	MF	217	20	0.0132399
GO:0033130	acetylcholine receptor binding	MF	8	3	0.01328804
GO:0061630	ubiquitin protein ligase activity	MF	188	17	0.01383422
GO:0042157	lipoprotein metabolic process	BP	202	17	0.01394988
GO:0022614	membrane to membrane docking	BP	4	2	0.01396601
GO:0005883	neurofilament	CC	8	3	0.01397079
GO:1903223	positive regulation of oxidative stress-induced neuron death	BP	3	2	0.01408213
GO:1903378	positive regulation of oxidative stress-induced neuron intrinsic apoptotic signal	BP	3	2	0.01408213
GO:0051893	regulation of focal adhesion assembly	BP	49	8	0.01428769
GO:0090109	regulation of cell-substrate junction assembly	BP	49	8	0.01428769
GO:0035384	thioester biosynthetic process	BP	62	8	0.01432758
GO:0071616	acyl-CoA biosynthetic process	BP	62	8	0.01432758
GO:0002438	acute inflammatory response to antigenic stimulus	BP	20	4	0.01454009
GO:0000827	inositol-1,3,4,5,6-pentakisphosphate kinase activity	MF	5	2	0.01454804
GO:0000828	inositol hexakisphosphate kinase activity	MF	5	2	0.01454804
GO:0000829	inositol heptakisphosphate kinase activity	MF	5	2	0.01454804

GO:0000832	inositol hexakisphosphate 5-kinase activity	MF	5	2	0.01454804
GO:0052723	inositol hexakisphosphate 1-kinase activity	MF	5	2	0.01454804
GO:0052724	inositol hexakisphosphate 3-kinase activity	MF	5	2	0.01454804
GO:0008287	protein serine/threonine phosphatase complex	CC	48	7	0.01457179
GO:1903293	phosphatase complex	CC	48	7	0.01457179
GO:0042752	regulation of circadian rhythm	BP	95	11	0.01480754
GO:0044613	nuclear pore central transport channel	CC	5	2	0.01488118
GO:0060448	dichotomous subdivision of terminal units involved in lung branching	BP	3	2	0.01533521
GO:0090521	glomerular visceral epithelial cell migration	BP	4	2	0.01539861
GO:0071331	cellular response to hexose stimulus	BP	114	13	0.01556402
GO:0035773	insulin secretion involved in cellular response to glucose stimulus	BP	55	8	0.01601331
GO:0032991	macromolecular complex	CC	4509	260	0.01614727
GO:1903203	regulation of oxidative stress-induced neuron death	BP	18	4	0.01616113
GO:0071326	cellular response to monosaccharide stimulus	BP	115	13	0.01627942
GO:0006084	acetyl-CoA metabolic process	BP	32	5	0.01632276
GO:0005524	ATP binding	MF	1400	96	0.01644428
GO:0004298	threonine-type endopeptidase activity	MF	21	4	0.01651414
GO:0070003	threonine-type peptidase activity	MF	21	4	0.01651414
GO:0018345	protein palmitoylation	BP	22	4	0.01655366
GO:0061659	ubiquitin-like protein ligase activity	MF	193	17	0.01670946
GO:0042175	nuclear outer membrane-endoplasmic reticulum membrane network	CC	963	63	0.01676647
GO:0038044	transforming growth factor-beta secretion	BP	5	2	0.01678517
GO:0030029	actin filament-based process	BP	639	51	0.01694042
GO:0006066	alcohol metabolic process	BP	284	22	0.01717231
GO:0070663	regulation of leukocyte proliferation	BP	193	16	0.01719067
GO:0051817	modification of morphology or physiology of other organism involved in symbic	BP	91	10	0.01729313
GO:0005839	proteasome core complex	CC	21	4	0.01731163
GO:0043001	Golgi to plasma membrane protein transport	BP	30	5	0.01744506
GO:0032555	purine ribonucleotide binding	MF	1748	114	0.01748953
GO:0071875	adrenergic receptor signaling pathway	BP	20	4	0.01755877

GO:0036475	neuron death in response to oxidative stress	BP	19	4	0.01763383
GO:0032484	Ral protein signal transduction	BP	3	2	0.01779056
GO:0032485	regulation of Ral protein signal transduction	BP	3	2	0.01779056
GO:0030292	protein tyrosine kinase inhibitor activity	MF	4	2	0.01798438
GO:0006596	polyamine biosynthetic process	BP	11	3	0.01816255
GO:0045727	positive regulation of translation	BP	98	11	0.01823228
GO:0060211	regulation of nuclear-transcribed mRNA poly(A) tail shortening	BP	11	3	0.01823919
GO:0060213	positive regulation of nuclear-transcribed mRNA poly(A) tail shortening	BP	11	3	0.01823919
GO:1900045	negative regulation of protein K63-linked ubiquitination	BP	4	2	0.01854957
GO:1902915	negative regulation of protein polyubiquitination	BP	4	2	0.01854957
GO:2000257	regulation of protein activation cascade	BP	31	4	0.01940429
GO:0034399	nuclear periphery	CC	117	13	0.0194098
GO:0034605	cellular response to heat	BP	112	12	0.01971922
GO:0097367	carbohydrate derivative binding	MF	2086	132	0.01973254
GO:0019706	protein-cysteine S-palmitoyltransferase activity	MF	24	4	0.01976061
GO:0019707	protein-cysteine S-acyltransferase activity	MF	24	4	0.01976061
GO:0014731	spectrin-associated cytoskeleton	CC	8	3	0.01983297
GO:0051953	negative regulation of amine transport	BP	21	4	0.01987966
GO:0002857	positive regulation of natural killer cell mediated immune response to tumor ce	BP	7	2	0.02037637
GO:0002860	positive regulation of natural killer cell mediated cytotoxicity directed against tu	BP	7	2	0.02037637
GO:0009311	oligosaccharide metabolic process	BP	53	7	0.02037961
GO:0017076	purine nucleotide binding	MF	1761	114	0.02051775
GO:1903391	regulation of adherens junction organization	BP	52	8	0.02062776
GO:0007218	neuropeptide signaling pathway	BP	95	9	0.02117175
GO:0090534	calcium ion-transporting ATPase complex	CC	1	1	0.02117787
GO:0032819	positive regulation of natural killer cell proliferation	BP	7	2	0.02118271
GO:0023051	regulation of signaling	BP	2929	185	0.02133427
GO:0046466	membrane lipid catabolic process	BP	23	4	0.0214497
GO:0051701	interaction with host	BP	187	17	0.02155885
GO:0070534	protein K63-linked ubiquitination	BP	41	6	0.02162296

GO:0032553	ribonucleotide binding	MF	1765	114	0.02186986
GO:0005672	transcription factor TFIIA complex	CC	5	2	0.02187438
GO:0005280	hydrogen:amino acid symporter activity	MF	6	2	0.02203459
GO:0044711	single-organism biosynthetic process	BP	1404	86	0.02204823
GO:0086103	G-protein coupled receptor signaling pathway involved in heart process	BP	9	3	0.0223807
GO:0016831	carboxy-lyase activity	MF	34	5	0.02257144
GO:0045670	regulation of osteoclast differentiation	BP	61	7	0.02257337
GO:0046904	calcium oxalate binding	MF	1	1	0.02266888
GO:0090407	organophosphate biosynthetic process	BP	613	43	0.02277382
GO:0045189	connective tissue growth factor biosynthetic process	BP	1	1	0.02280599
GO:0070351	negative regulation of white fat cell proliferation	BP	1	1	0.02280599
GO:0005744	mitochondrial inner membrane presequence translocase complex	CC	7	2	0.02295781
GO:0005912	adherens junction	CC	474	40	0.02298922
GO:0042523	positive regulation of tyrosine phosphorylation of Stat5 protein	BP	16	3	0.02305059
GO:0005789	endoplasmic reticulum membrane	CC	944	61	0.02326598
GO:0044267	cellular protein metabolic process	BP	4569	259	0.02328502
GO:0002673	regulation of acute inflammatory response	BP	69	7	0.02332521
GO:0045586	regulation of gamma-delta T cell differentiation	BP	5	2	0.02336352
GO:0043647	inositol phosphate metabolic process	BP	65	8	0.02341029
GO:0071889	14-3-3 protein binding	MF	23	5	0.02372854
GO:0009214	cyclic nucleotide catabolic process	BP	22	5	0.02381331
GO:0071322	cellular response to carbohydrate stimulus	BP	122	13	0.0240213
GO:0045071	negative regulation of viral genome replication	BP	50	6	0.02427301
GO:1902047	polyamine transmembrane transport	BP	5	2	0.02428517
GO:1902267	regulation of polyamine transmembrane transport	BP	5	2	0.02428517
GO:0071495	cellular response to endogenous stimulus	BP	1187	83	0.02440876
GO:0046030	inositol trisphosphate phosphatase activity	MF	10	3	0.02453239
GO:0052743	inositol tetrakisphosphate phosphatase activity	MF	10	3	0.02455946
GO:0006595	polyamine metabolic process	BP	13	3	0.02464934
GO:0015937	coenzyme A biosynthetic process	BP	10	3	0.02468138

GO:2000266	regulation of blood coagulation, intrinsic pathway	BP	1	1	0.0248349
GO:0034250	positive regulation of cellular amide metabolic process	BP	114	12	0.02486851
GO:0032021	NELF complex	CC	4	2	0.02488326
GO:0060600	dichotomous subdivision of an epithelial terminal unit	BP	10	3	0.02491386
GO:0002355	detection of tumor cell	BP	1	1	0.02494431
GO:0016363	nuclear matrix	CC	96	11	0.02495011
GO:0034390	smooth muscle cell apoptotic process	BP	15	3	0.0252691
GO:0034391	regulation of smooth muscle cell apoptotic process	BP	15	3	0.0252691
GO:0046488	phosphatidylinositol metabolic process	BP	207	18	0.02533613
GO:0006929	substrate-dependent cell migration	BP	27	5	0.02544985
GO:0050670	regulation of lymphocyte proliferation	BP	185	15	0.0254955
GO:0003363	lamellipodium assembly involved in ameboidal cell migration	BP	1	1	0.02558725
GO:0021816	extension of a leading process involved in cell motility in cerebral cortex radial [illegible]	BP	1	1	0.02558725
GO:0009313	oligosaccharide catabolic process	BP	13	3	0.02559602
GO:0001621	ADP receptor activity	MF	1	1	0.02612554
GO:0032944	regulation of mononuclear cell proliferation	BP	186	15	0.02630872
GO:0016740	transferase activity	MF	2241	137	0.02638788
GO:0086089	voltage-gated potassium channel activity involved in atrial cardiac muscle cell a	MF	4	2	0.02665573
GO:0006650	glycerophospholipid metabolic process	BP	331	26	0.02679046
GO:0010646	regulation of cell communication	BP	2879	181	0.02679276
GO:0043902	positive regulation of multi-organism process	BP	157	14	0.02689547
GO:0045655	regulation of monocyte differentiation	BP	18	4	0.02691114
GO:0046317	regulation of glucosylceramide biosynthetic process	BP	1	1	0.02703294
GO:0046318	negative regulation of glucosylceramide biosynthetic process	BP	1	1	0.02703294
GO:0046879	hormone secretion	BP	280	24	0.02722349
GO:1901622	positive regulation of smoothened signaling pathway involved in dorsal/ventral	BP	1	1	0.02733785
GO:0008340	determination of adult lifespan	BP	12	3	0.02748455
GO:0034198	cellular response to amino acid starvation	BP	36	5	0.02752315
GO:0042771	intrinsic apoptotic signaling pathway in response to DNA damage by p53 class r	BP	42	6	0.02760919
GO:0004523	RNA-DNA hybrid ribonuclease activity	MF	6	2	0.02772026

GO:0080008	Cul4-RING E3 ubiquitin ligase complex	CC	25	4	0.02783664
GO:0046949	fatty-acyl-CoA biosynthetic process	BP	43	6	0.02790741
GO:0003972	RNA ligase (ATP) activity	MF	1	1	0.02793843
GO:0008452	RNA ligase activity	MF	1	1	0.02793843
GO:0016925	protein sumoylation	BP	87	9	0.0280248
GO:0070535	histone H2A K63-linked ubiquitination	BP	5	2	0.02802512
GO:0006999	nuclear pore organization	BP	15	3	0.02802847
GO:0000165	MAPK cascade	BP	824	57	0.0280611
GO:0004115	3',5'-cyclic-AMP phosphodiesterase activity	MF	15	4	0.02822895
GO:0045779	negative regulation of bone resorption	BP	12	3	0.02858834
GO:0002420	natural killer cell mediated cytotoxicity directed against tumor cell target	BP	9	2	0.02895078
GO:0002423	natural killer cell mediated immune response to tumor cell	BP	9	2	0.02895078
GO:0002855	regulation of natural killer cell mediated immune response to tumor cell	BP	9	2	0.02895078
GO:0002858	regulation of natural killer cell mediated cytotoxicity directed against tumor cel	BP	9	2	0.02895078
GO:0004691	cAMP-dependent protein kinase activity	MF	6	2	0.02895939
GO:0045954	positive regulation of natural killer cell mediated cytotoxicity	BP	17	3	0.02917344
GO:1902914	regulation of protein polyubiquitination	BP	13	3	0.02923699
GO:0034511	U3 snoRNA binding	MF	6	2	0.02925924
GO:1902117	positive regulation of organelle assembly	BP	57	7	0.02925979
GO:0004588	orotate phosphoribosyltransferase activity	MF	1	1	0.02931349
GO:0004590	orotidine-5'-phosphate decarboxylase activity	MF	1	1	0.02931349
GO:0048583	regulation of response to stimulus	BP	3404	204	0.02933015
GO:0015919	peroxisomal membrane transport	BP	6	2	0.02940038
GO:0045046	protein import into peroxisome membrane	BP	6	2	0.02940038
GO:0045671	negative regulation of osteoclast differentiation	BP	25	4	0.02953424
GO:0061077	chaperone-mediated protein folding	BP	64	7	0.02957994
GO:0048002	antigen processing and presentation of peptide antigen	BP	171	15	0.02966673
GO:0015805	S-adenosyl-L-methionine transport	BP	1	1	0.02967396
GO:1901962	S-adenosyl-L-methionine transmembrane transport	BP	1	1	0.02967396
GO:0000095	S-adenosyl-L-methionine transmembrane transporter activity	MF	1	1	0.02967396

GO:0001931	uropod	CC	12	3	0.02987497
GO:0031254	cell trailing edge	CC	12	3	0.02987497
GO:0098590	plasma membrane region	CC	904	65	0.02991434
GO:0045069	regulation of viral genome replication	BP	79	8	0.02994843
GO:0047290	(alpha-N-acetylneuraminyl-2,3-beta-galactosyl-1,3)-N-acetyl-galactosaminide 6	MF	1	1	0.03000895
GO:0007254	JNK cascade	BP	182	17	0.03001442
GO:0072655	establishment of protein localization to mitochondrion	BP	184	16	0.03001671
GO:0021532	neural tube patterning	BP	38	6	0.03003212
GO:0005516	calmodulin binding	MF	183	18	0.03012825
GO:0042164	interleukin-12 alpha subunit binding	MF	1	1	0.0301736
GO:0007196	adenylate cyclase-inhibiting G-protein coupled glutamate receptor signaling pa	BP	9	3	0.0302033
GO:0034284	response to monosaccharide	BP	183	17	0.03026816
GO:0014043	negative regulation of neuron maturation	BP	4	2	0.03032451
GO:0038066	p38MAPK cascade	BP	33	5	0.03046748
GO:0072591	citrate-L-glutamate ligase activity	MF	1	1	0.03063681
GO:2000779	regulation of double-strand break repair	BP	43	6	0.03064347
GO:0006657	CDP-choline pathway	BP	6	2	0.03097751
GO:1904247	positive regulation of polynucleotide adenylyltransferase activity	BP	1	1	0.03097832
GO:0044422	organelle part	CC	8376	450	0.03100172
GO:0051425	PTB domain binding	MF	4	2	0.03110507
GO:0002037	negative regulation of L-glutamate transport	BP	1	1	0.03134414
GO:0001678	cellular glucose homeostasis	BP	124	13	0.03138965
GO:0023014	signal transduction by protein phosphorylation	BP	859	59	0.03183465
GO:0006687	glycosphingolipid metabolic process	BP	59	7	0.03195093
GO:0050778	positive regulation of immune response	BP	621	40	0.03199232
GO:0070585	protein localization to mitochondrion	BP	186	16	0.03210575
GO:1901888	regulation of cell junction assembly	BP	71	9	0.03233193
GO:1903959	regulation of anion transmembrane transport	BP	32	5	0.03248225
GO:0070161	anchoring junction	CC	490	40	0.03250913
GO:0051886	negative regulation of timing of anagen	BP	1	1	0.0326161

GO:2001203	positive regulation of transforming growth factor-beta secretion	BP	1	1	0.0326161
GO:0000268	peroxisome targeting sequence binding	MF	5	2	0.03263853
GO:1903960	negative regulation of anion transmembrane transport	BP	10	3	0.03275077
GO:1900130	regulation of lipid binding	BP	1	1	0.03277561
GO:1900131	negative regulation of lipid binding	BP	1	1	0.03277561
GO:0033328	peroxisome membrane targeting sequence binding	MF	1	1	0.03277561
GO:0036105	peroxisome membrane class-1 targeting sequence binding	MF	1	1	0.03277561
GO:0097110	scaffold protein binding	MF	46	7	0.0328974
GO:0044089	positive regulation of cellular component biogenesis	BP	442	35	0.0329127
GO:1902115	regulation of organelle assembly	BP	137	13	0.03305551
GO:0071788	endoplasmic reticulum tubular network maintenance	BP	1	1	0.03325375
GO:0098826	endoplasmic reticulum tubular network membrane	CC	1	1	0.03325375
GO:0035336	long-chain fatty-acyl-CoA metabolic process	BP	46	6	0.03335009
GO:0050730	regulation of peptidyl-tyrosine phosphorylation	BP	215	18	0.0337464
GO:0050656	3'-phosphoadenosine 5'-phosphosulfate binding	MF	6	2	0.03376717
GO:0032817	regulation of natural killer cell proliferation	BP	9	2	0.03405796
GO:0006661	phosphatidylinositol biosynthetic process	BP	132	13	0.03410812
GO:1990138	neuron projection extension	BP	130	15	0.03415909
GO:0005829	cytosol	CC	4535	257	0.03418401
GO:0006464	cellular protein modification process	BP	3633	212	0.03424801
GO:0036211	protein modification process	BP	3633	212	0.03424801
GO:0048104	establishment of body hair or bristle planar orientation	BP	4	2	0.03427445
GO:0048105	establishment of body hair planar orientation	BP	4	2	0.03427445
GO:0006689	ganglioside catabolic process	BP	6	2	0.03431465
GO:0006796	phosphate-containing compound metabolic process	BP	2934	175	0.03447154
GO:1904429	regulation of t-circle formation	BP	5	2	0.03451695
GO:0002230	positive regulation of defense response to virus by host	BP	19	3	0.03458278
GO:0030101	natural killer cell activation	BP	57	6	0.03484002
GO:0090502	RNA phosphodiester bond hydrolysis, endonucleolytic	BP	63	7	0.03493938
GO:0051132	NK T cell activation	BP	9	2	0.03501998

GO:0016417	S-acyltransferase activity	MF	28	4	0.03502467
GO:0035821	modification of morphology or physiology of other organism	BP	111	10	0.03508084
GO:0061669	spontaneous neurotransmitter secretion	BP	4	2	0.0351669
GO:0098814	spontaneous synaptic transmission	BP	4	2	0.0351669
GO:0009914	hormone transport	BP	290	24	0.03522836
GO:0006793	phosphorus metabolic process	BP	3018	179	0.03542398
GO:0030148	sphingolipid biosynthetic process	BP	82	9	0.03542701
GO:1900273	positive regulation of long-term synaptic potentiation	BP	11	3	0.03544204
GO:0017125	deoxycytidyl transferase activity	MF	1	1	0.03546927
GO:0002684	positive regulation of immune system process	BP	873	54	0.03549935
GO:0006671	phytosphingosine metabolic process	BP	1	1	0.03570446
GO:0071602	phytosphingosine biosynthetic process	BP	1	1	0.03570446
GO:0070774	phytoceramidase activity	MF	1	1	0.03570446
GO:0031867	EP4 subtype prostaglandin E2 receptor binding	MF	1	1	0.03571057
GO:0044743	protein transmembrane import into intracellular organelle	BP	40	5	0.03580137
GO:0042999	regulation of Golgi to plasma membrane CFTR protein transport	BP	1	1	0.03583594
GO:0043002	negative regulation of Golgi to plasma membrane CFTR protein transport	BP	1	1	0.03583594
GO:0043004	cytoplasmic sequestering of CFTR protein	BP	1	1	0.03583594
GO:0033768	SUMO-targeted ubiquitin ligase complex	CC	1	1	0.03601638
GO:1903861	positive regulation of dendrite extension	BP	17	4	0.03611597
GO:0001325	formation of extrachromosomal circular DNA	BP	13	3	0.03624075
GO:0090656	t-circle formation	BP	13	3	0.03624075
GO:0090737	telomere maintenance via telomere trimming	BP	13	3	0.03624075
GO:0006684	sphingomyelin metabolic process	BP	14	3	0.03624968
GO:0016459	myosin complex	CC	70	8	0.03630229
GO:0006367	transcription initiation from RNA polymerase II promoter	BP	164	15	0.03635418
GO:0090386	phagosome maturation involved in apoptotic cell clearance	BP	1	1	0.03637399
GO:0090387	phagolysosome assembly involved in apoptotic cell clearance	BP	1	1	0.03637399
GO:0090389	phagosome-lysosome fusion involved in apoptotic cell clearance	BP	1	1	0.03637399
GO:0001665	alpha-N-acetylgalactosaminide alpha-2,6-sialyltransferase activity	MF	6	2	0.03662235

GO:0042979	ornithine decarboxylase regulator activity	MF	6	2	0.03684325
GO:0031526	brush border membrane	CC	49	6	0.03684393
GO:0090118	receptor-mediated endocytosis involved in cholesterol transport	BP	5	2	0.03715919
GO:0035556	intracellular signal transduction	BP	2439	151	0.03740494
GO:0006397	mRNA processing	BP	441	31	0.03746179
GO:0090259	regulation of retinal ganglion cell axon guidance	BP	5	2	0.03759922
GO:0048511	rhythmic process	BP	304	24	0.0376298
GO:0043130	ubiquitin binding	MF	101	10	0.03774328
GO:1901077	regulation of relaxation of muscle	BP	13	3	0.03778629
GO:1904358	positive regulation of telomere maintenance via telomere lengthening	BP	34	5	0.03781866
GO:0023061	signal release	BP	398	32	0.03816978
GO:0008049	male courtship behavior	BP	1	1	0.03817971
GO:0061734	parkin-mediated stimulation of mitophagy in response to mitochondrial depola	BP	5	2	0.03841248
GO:0035508	positive regulation of myosin-light-chain-phosphatase activity	BP	1	1	0.03845996
GO:0051246	regulation of protein metabolic process	BP	2395	143	0.03859116
GO:0005643	nuclear pore	CC	73	7	0.03869533
GO:0080163	regulation of protein serine/threonine phosphatase activity	BP	1	1	0.03904401
GO:1900073	regulation of neuromuscular synaptic transmission	BP	1	1	0.03909406
GO:1900075	positive regulation of neuromuscular synaptic transmission	BP	1	1	0.03909406
GO:0060405	regulation of penile erection	BP	6	2	0.03923283
GO:1905636	positive regulation of RNA polymerase II regulatory region sequence-specific DI	BP	1	1	0.03932013
GO:0002836	positive regulation of response to tumor cell	BP	9	2	0.0394745
GO:0002839	positive regulation of immune response to tumor cell	BP	9	2	0.0394745
GO:0005861	troponin complex	CC	8	2	0.03950493
GO:0006506	GPI anchor biosynthetic process	BP	30	4	0.03953397
GO:0044425	membrane part	CC	6037	317	0.03957337
GO:1901078	negative regulation of relaxation of muscle	BP	5	2	0.03961167
GO:0060560	developmental growth involved in morphogenesis	BP	191	20	0.03974058
GO:0017178	diphthine-ammonia ligase activity	MF	1	1	0.0399567
GO:1903792	negative regulation of anion transport	BP	22	4	0.03997129

GO:0035255	ionotropic glutamate receptor binding	MF	20	4	0.03998907
GO:0032056	positive regulation of translation in response to stress	BP	6	2	0.04002292
GO:0035693	NOS2-CD74 complex	CC	1	1	0.0401377
GO:0035718	macrophage migration inhibitory factor binding	MF	1	1	0.0401377
GO:0042658	MHC class II protein binding, via antigen binding groove	MF	1	1	0.0401377
GO:0005903	brush border	CC	93	10	0.04022848
GO:0071310	cellular response to organic substance	BP	2134	131	0.04036646
GO:0009749	response to glucose	BP	173	16	0.04038792
GO:0019538	protein metabolic process	BP	5123	283	0.04065971
GO:0035639	purine ribonucleoside triphosphate binding	MF	1706	108	0.04073502
GO:0006085	acetyl-CoA biosynthetic process	BP	18	3	0.04084107
GO:0070887	cellular response to chemical stimulus	BP	2584	154	0.04088728
GO:0050691	regulation of defense response to virus by host	BP	31	4	0.04095202
GO:0048256	flap endonuclease activity	MF	6	2	0.04098976
GO:0007160	cell-matrix adhesion	BP	192	18	0.0412168
GO:0046643	regulation of gamma-delta T cell activation	BP	7	2	0.04124802
GO:0033857	diphosphoinositol-pentakisphosphate kinase activity	MF	2	1	0.04131061
GO:0090090	negative regulation of canonical Wnt signaling pathway	BP	161	15	0.04140686
GO:0042129	regulation of T cell proliferation	BP	140	11	0.04148912
GO:0004445	inositol-polyphosphate 5-phosphatase activity	MF	5	2	0.04162157
GO:0018160	peptidyl-pyrromethane cofactor linkage	BP	1	1	0.04162557
GO:0004418	hydroxymethylbilane synthase activity	MF	1	1	0.04162557
GO:0036064	ciliary basal body	CC	94	9	0.04186574
GO:0044828	negative regulation by host of viral genome replication	BP	5	2	0.04187006
GO:0070986	left/right axis specification	BP	13	3	0.04208399
GO:1900036	positive regulation of cellular response to heat	BP	1	1	0.04213934
GO:0030263	apoptotic chromosome condensation	BP	5	2	0.04214868
GO:0050806	positive regulation of synaptic transmission	BP	119	13	0.04229792
GO:0016604	nuclear body	CC	673	48	0.04233445
GO:0000796	condensin complex	CC	7	2	0.04257256

GO:2000319	regulation of T-helper 17 cell differentiation	BP	10	2	0.04281405
GO:0043031	negative regulation of macrophage activation	BP	8	2	0.04297666
GO:0007045	cell-substrate adherens junction assembly	BP	68	9	0.04300132
GO:0048041	focal adhesion assembly	BP	68	9	0.04300132
GO:0030302	deoxynucleotide transport	BP	1	1	0.04300693
GO:0030974	thiamine pyrophosphate transport	BP	1	1	0.04300693
GO:0030233	deoxynucleotide transmembrane transporter activity	MF	1	1	0.04300693
GO:0090422	thiamine pyrophosphate transporter activity	MF	1	1	0.04300693
GO:0044087	regulation of cellular component biogenesis	BP	761	55	0.04302278
GO:0006505	GPI anchor metabolic process	BP	31	4	0.04303704
GO:0001573	ganglioside metabolic process	BP	15	3	0.04321035
GO:0034333	adherens junction assembly	BP	78	10	0.0432404
GO:0033158	regulation of protein import into nucleus, translocation	BP	18	3	0.04351022
GO:0005388	calcium-transporting ATPase activity	MF	13	3	0.0435773
GO:0004529	exodeoxyribonuclease activity	MF	17	3	0.04404099
GO:0016895	exodeoxyribonuclease activity, producing 5'-phosphomonoesters	MF	17	3	0.04404099
GO:0098914	membrane repolarization during atrial cardiac muscle cell action potential	BP	5	2	0.04416072
GO:0006352	DNA-templated transcription, initiation	BP	220	18	0.04425791
GO:0070647	protein modification by small protein conjugation or removal	BP	1026	65	0.04439083
GO:0006686	sphingomyelin biosynthetic process	BP	6	2	0.04439781
GO:0004867	serine-type endopeptidase inhibitor activity	MF	87	7	0.04459125
GO:0032780	negative regulation of ATPase activity	BP	9	2	0.04470934
GO:0006487	protein N-linked glycosylation	BP	65	7	0.04481264
GO:0031974	membrane-enclosed lumen	CC	4643	261	0.04488453
GO:0043233	organelle lumen	CC	4643	261	0.04488453
GO:0070013	intracellular organelle lumen	CC	4643	261	0.04488453
GO:0015187	glycine transmembrane transporter activity	MF	7	2	0.0449844
GO:1902177	positive regulation of oxidative stress-induced intrinsic apoptotic signaling path	BP	6	2	0.04506931
GO:0004132	dCMP deaminase activity	MF	1	1	0.04511259
GO:0046851	negative regulation of bone remodeling	BP	14	3	0.04512044

GO:0072594	establishment of protein localization to organelle	BP	630	42	0.04512682
GO:0048488	synaptic vesicle endocytosis	BP	29	5	0.04518742
GO:0018738	S-formylglutathione hydrolase activity	MF	1	1	0.04527032
GO:0030864	cortical actin cytoskeleton	CC	52	7	0.04527426
GO:0033365	protein localization to organelle	BP	873	57	0.04533228
GO:0015936	coenzyme A metabolic process	BP	14	3	0.04533797
GO:0006463	steroid hormone receptor complex assembly	BP	1	1	0.04535339
GO:0002437	inflammatory response to antigenic stimulus	BP	40	5	0.0453541
GO:0070208	protein heterotrimerization	BP	13	3	0.04545941
GO:0060291	long-term synaptic potentiation	BP	54	7	0.04548486
GO:0001787	natural killer cell proliferation	BP	11	2	0.0455515
GO:0003844	1,4-alpha-glucan branching enzyme activity	MF	1	1	0.04560608
GO:0002717	positive regulation of natural killer cell mediated immunity	BP	19	3	0.04561164
GO:0003916	DNA topoisomerase activity	MF	7	2	0.0456179
GO:0035732	nitric oxide storage	BP	1	1	0.04579248
GO:0035730	S-nitrosoglutathione binding	MF	1	1	0.04579248
GO:0035731	dinitrosyl-iron complex binding	MF	1	1	0.04579248
GO:0043651	linoleic acid metabolic process	BP	16	3	0.04585591
GO:0010477	response to sulfur dioxide	BP	1	1	0.04590476
GO:1900151	regulation of nuclear-transcribed mRNA catabolic process, deadenylation-depe	BP	16	3	0.04595086
GO:1900153	positive regulation of nuclear-transcribed mRNA catabolic process, deadenylati	BP	16	3	0.04595086
GO:0015846	polyamine transport	BP	7	2	0.0461413
GO:0009154	purine ribonucleotide catabolic process	BP	31	5	0.04622128
GO:0004586	ornithine decarboxylase activity	MF	1	1	0.04624372
GO:0044444	cytoplasmic part	CC	8613	457	0.04637099
GO:1903900	regulation of viral life cycle	BP	174	14	0.04637615
GO:1903902	positive regulation of viral life cycle	BP	93	9	0.04648108
GO:0003713	transcription coactivator activity	MF	283	23	0.04648773
GO:0032433	filopodium tip	CC	12	3	0.04665599
GO:0043168	anion binding	MF	2523	153	0.04691102

GO:0032839	dendrite cytoplasm	CC	21	4	0.04702615
GO:0007623	circadian rhythm	BP	190	16	0.04711311
GO:0007274	neuromuscular synaptic transmission	BP	29	4	0.04748668
GO:0007166	cell surface receptor signaling pathway	BP	2480	153	0.04753355
GO:0008753	NADPH dehydrogenase (quinone) activity	MF	1	1	0.04768257
GO:0033116	endoplasmic reticulum-Golgi intermediate compartment membrane	CC	65	7	0.04773445
GO:0008117	sphinganine-1-phosphate aldolase activity	MF	1	1	0.04780014
GO:0009746	response to hexose	BP	178	16	0.0478675
GO:0051908	double-stranded DNA 5'-3' exodeoxyribonuclease activity	MF	1	1	0.04789316
GO:0003746	translation elongation factor activity	MF	16	3	0.04819121
GO:0000289	nuclear-transcribed mRNA poly(A) tail shortening	BP	35	5	0.04822575
GO:0002296	T-helper 1 cell lineage commitment	BP	2	1	0.04835887
GO:0030228	lipoprotein particle receptor activity	MF	16	3	0.0487206
GO:0004361	glutaryl-CoA dehydrogenase activity	MF	1	1	0.04891465
GO:0032601	connective tissue growth factor production	BP	2	1	0.04904092
GO:0005925	focal adhesion	CC	378	31	0.0490874
GO:0030048	actin filament-based movement	BP	117	12	0.04916131
GO:1902080	regulation of calcium ion import into sarcoplasmic reticulum	BP	2	1	0.04938843
GO:1902081	negative regulation of calcium ion import into sarcoplasmic reticulum	BP	2	1	0.04938843
GO:0044448	cell cortex part	CC	114	12	0.04944927
GO:0000921	septin ring assembly	BP	1	1	0.0496141
GO:0031106	septin ring organization	BP	1	1	0.0496141
GO:0000209	protein polyubiquitination	BP	278	21	0.04963809
GO:0008135	translation factor activity, RNA binding	MF	77	8	0.04964337
GO:0006409	tRNA export from nucleus	BP	33	4	0.04968908
GO:0071431	tRNA-containing ribonucleoprotein complex export from nucleus	BP	33	4	0.04968908
GO:1901876	regulation of calcium ion binding	BP	2	1	0.04982339
GO:1901877	negative regulation of calcium ion binding	BP	2	1	0.04982339
