# Supplementary material for: Increased DNA methylation variability in rheumatoid arthritis-discordant monozygotic twins
Source: Genome Med. 2018 Sep 4;10:64. doi: 10.1186/s13073-018-0575-9 (PMC6122744; doi:10.1186/s13073-018-0575-9)
Supplement: Supplementary file 4 — Table S3. Pathways enriched in differentially variable positions identified in RA-discordant twins, restricted to sites which were hypervariable in healthy co-twins. Pathway analysis was performed using the gometh function in the MissMethyl package. Pathways are ranked by p value (p < 0.05). (PDF 87 KB) (PDF 84 kb) [file 13073_2018_575_MOESM4_ESM.pdf]

Additional file 4: Table S3

Pathways enriched in DVPs identified in RA discordant twins, which were hypervariable in healthy co-twins

	Term	Ontology	No. of genes in GO term	No. of genes with DVP	P-val
GO:0035745	T-helper 2 cell cytokine production	BP	9	3	0.00050456
GO:0038189	neuropilin signaling pathway	BP	2	2	0.00068662
GO:0038190	VEGF-activated neuropilin signaling pathway	BP	2	2	0.00068662
GO:1902336	positive regulation of retinal ganglion cell axon guidance	BP	2	2	0.00068662
GO:0035743	CD4-positive, alpha-beta T cell cytokine production	BP	11	3	0.00072328
GO:0046930	pore complex	CC	17	3	0.00100638
GO:1904430	negative regulation of t-circle formation	BP	3	2	0.00124238
GO:0016323	basolateral plasma membrane	CC	192	11	0.00129969
GO:0042092	type 2 immune response	BP	32	4	0.0015885
GO:0018193	peptidyl-amino acid modification	BP	1125	35	0.00197866
GO:0002526	acute inflammatory response	BP	129	7	0.00225049
GO:0015629	actin cytoskeleton	CC	430	19	0.00235134
GO:0042522	regulation of tyrosine phosphorylation of Stat5 protein	BP	19	3	0.00258606
GO:1905898	positive regulation of response to endoplasmic reticulum stress	BP	32	4	0.00285081
GO:0038066	p38MAPK cascade	BP	33	4	0.00318725
GO:0042506	tyrosine phosphorylation of Stat5 protein	BP	22	3	0.00389153
GO:0030033	microvillus assembly	BP	15	3	0.00437349
GO:1904429	regulation of t-circle formation	BP	5	2	0.00448695
GO:0014043	negative regulation of neuron maturation	BP	4	2	0.00459183
GO:2000257	regulation of protein activation cascade	BP	31	3	0.00471381
GO:0090259	regulation of retinal ganglion cell axon guidance	BP	5	2	0.00521307
GO:0002367	cytokine production involved in immune response	BP	71	5	0.00565376
GO:0043031	negative regulation of macrophage activation	BP	8	2	0.00577091
GO:0035092	sperm chromatin condensation	BP	8	2	0.00664808
GO:0002369	T cell cytokine production	BP	25	3	0.00669391
GO:0044425	membrane part	CC	6037	121	0.00678168

GO:1901142	insulin metabolic process	BP	7	2	0.00698178
GO:0071679	commissural neuron axon guidance	BP	7	2	0.00702877
GO:0006352	DNA-templated transcription, initiation	BP	220	10	0.00707583
GO:0045189	connective tissue growth factor biosynthetic process	BP	1	1	0.00774631
GO:0070351	negative regulation of white fat cell proliferation	BP	1	1	0.00774631
GO:0008375	acetylglucosaminyltransferase activity	MF	44	4	0.007937
GO:0005516	calmodulin binding	MF	183	10	0.00821202
GO:0042771	intrinsic apoptotic signaling pathway in response to DNA damag	BP	42	4	0.00843215
GO:1903430	negative regulation of cell maturation	BP	8	2	0.00853854
GO:1900073	regulation of neuromuscular synaptic transmission	BP	1	1	0.00907213
GO:1900075	positive regulation of neuromuscular synaptic transmission	BP	1	1	0.00907213
GO:2000266	regulation of blood coagulation, intrinsic pathway	BP	1	1	0.00927294
GO:0017178	diphthine-ammonia ligase activity	MF	1	1	0.00943707
GO:0035693	NOS2-CD74 complex	CC	1	1	0.0095178
GO:0035718	macrophage migration inhibitory factor binding	MF	1	1	0.0095178
GO:0042658	MHC class II protein binding, via antigen binding groove	MF	1	1	0.0095178
GO:0001650	fibrillar center	CC	123	7	0.00965147
GO:0003363	lamellipodium assembly involved in ameboidal cell migration	BP	1	1	0.00970185
GO:0021816	extension of a leading process involved in cell motility in cerebr	BP	1	1	0.00970185
GO:0044452	nucleolar part	CC	165	8	0.00975547
GO:0003707	steroid hormone receptor activity	MF	57	5	0.01005683
GO:0050670	regulation of lymphocyte proliferation	BP	185	8	0.01016606
GO:0018160	peptidyl-pyrromethane cofactor linkage	BP	1	1	0.01022891
GO:0004418	hydroxymethylbilane synthase activity	MF	1	1	0.01022891
GO:0032944	regulation of mononuclear cell proliferation	BP	186	8	0.01032138
GO:0032528	microvillus organization	BP	23	3	0.01083251
GO:0005741	mitochondrial outer membrane	CC	152	7	0.01102454
GO:1902946	protein localization to early endosome	BP	5	2	0.01104745
GO:0050863	regulation of T cell activation	BP	287	11	0.01118606
GO:0022407	regulation of cell-cell adhesion	BP	376	14	0.01120504

GO:0030134	ER to Golgi transport vesicle	CC	72	5	0.01127901
GO:0007159	leukocyte cell-cell adhesion	BP	330	12	0.0114309
GO:0050892	intestinal absorption	BP	33	3	0.01147124
GO:0080134	regulation of response to stress	BP	1243	33	0.01189664
GO:0004132	dCMP deaminase activity	MF	1	1	0.01205643
GO:0070663	regulation of leukocyte proliferation	BP	193	8	0.01232124
GO:0005903	brush border	CC	93	6	0.01242585
GO:0002376	immune system process	BP	2523	56	0.01245454
GO:0098858	actin-based cell projection	CC	178	9	0.01252132
GO:0004586	ornithine decarboxylase activity	MF	1	1	0.01266174
GO:0006367	transcription initiation from RNA polymerase II promoter	BP	164	8	0.01292805
GO:0016459	myosin complex	CC	70	5	0.01309414
GO:0001932	regulation of protein phosphorylation	BP	1224	34	0.0131191
GO:0090336	positive regulation of brown fat cell differentiation	BP	7	2	0.01315117
GO:0009925	basal plasma membrane	CC	29	3	0.01352338
GO:0006338	chromatin remodeling	BP	148	7	0.01379998
GO:0051879	Hsp90 protein binding	MF	29	3	0.01381742
GO:0033865	nucleoside bisphosphate metabolic process	BP	33	3	0.01406294
GO:0033875	ribonucleoside bisphosphate metabolic process	BP	33	3	0.01406294
GO:0034032	purine nucleoside bisphosphate metabolic process	BP	33	3	0.01406294
GO:0050671	positive regulation of lymphocyte proliferation	BP	122	6	0.01412344
GO:0008140	cAMP response element binding protein binding	MF	8	2	0.01431171
GO:0038024	cargo receptor activity	MF	62	4	0.01433985
GO:0032946	positive regulation of mononuclear cell proliferation	BP	123	6	0.0143852
GO:0001764	neuron migration	BP	127	8	0.01460395
GO:1900744	regulation of p38MAPK cascade	BP	27	3	0.01481563
GO:0002673	regulation of acute inflammatory response	BP	69	4	0.01481924
GO:0032839	dendrite cytoplasm	CC	21	3	0.01485293
GO:0043408	regulation of MAPK cascade	BP	632	20	0.01505771
GO:0060982	coronary artery morphogenesis	BP	8	2	0.01538823

GO:1903037	regulation of leukocyte cell-cell adhesion	BP	302	11	0.01588073
GO:0030175	filopodium	CC	91	6	0.01603668
GO:0045859	regulation of protein kinase activity	BP	708	22	0.01608828
GO:0045860	positive regulation of protein kinase activity	BP	460	16	0.01619488
GO:0048842	positive regulation of axon extension involved in axon guidance	BP	7	2	0.01624358
GO:1902669	positive regulation of axon guidance	BP	7	2	0.01624358
GO:0032872	regulation of stress-activated MAPK cascade	BP	193	9	0.0163416
GO:1903570	regulation of protein kinase D signaling	BP	1	1	0.01664116
GO:1903572	positive regulation of protein kinase D signaling	BP	1	1	0.01664116
GO:0042942	D-serine transport	BP	2	1	0.01671924
GO:0016900	oxidoreductase activity, acting on the CH-OH group of donors, d	MF	2	1	0.0167497
GO:0047057	vitamin-K-epoxide reductase (warfarin-sensitive) activity	MF	2	1	0.0167497
GO:0005883	neurofilament	CC	8	2	0.01714151
GO:0070665	positive regulation of leukocyte proliferation	BP	128	6	0.0171537
GO:0070302	regulation of stress-activated protein kinase signaling cascade	BP	194	9	0.01720096
GO:0002702	positive regulation of production of molecular mediator of imm	BP	68	4	0.01724893
GO:0051403	stress-activated MAPK cascade	BP	237	10	0.01757334
GO:0097475	motor neuron migration	BP	7	2	0.01759375
GO:0002296	T-helper 1 cell lineage commitment	BP	2	1	0.01762968
GO:0032601	connective tissue growth factor production	BP	2	1	0.01784725
GO:0000146	microfilament motor activity	MF	22	3	0.01786707
GO:0050730	regulation of peptidyl-tyrosine phosphorylation	BP	215	9	0.0180395
GO:0072677	eosinophil migration	BP	15	2	0.01813814
GO:0042129	regulation of T cell proliferation	BP	140	6	0.01825831
GO:0098590	plasma membrane region	CC	904	28	0.01844407
GO:0043030	regulation of macrophage activation	BP	31	3	0.01846585
GO:0001771	immunological synapse formation	BP	12	2	0.01849905
GO:0002920	regulation of humoral immune response	BP	46	3	0.01850794
GO:0002116	semaphorin receptor complex	CC	6	2	0.01909211
GO:0036290	protein trans-autophosphorylation	BP	1	1	0.0193822

GO:1990579	peptidyl-serine trans-autophosphorylation	BP	1	1	0.0193822
GO:1990332	Ire1 complex	CC	1	1	0.0193822
GO:0042523	positive regulation of tyrosine phosphorylation of Stat5 protein	BP	16	2	0.01947554
GO:0098665	serotonin receptor complex	CC	2	1	0.01981383
GO:1904602	serotonin-activated cation-selective channel complex	CC	2	1	0.01981383
GO:0005247	voltage-gated chloride channel activity	MF	10	2	0.01981988
GO:0060081	membrane hyperpolarization	BP	11	2	0.01993462
GO:0051174	regulation of phosphorus metabolic process	BP	1547	40	0.020126
GO:0015055	secretin receptor activity	MF	1	1	0.02043737
GO:0001935	endothelial cell proliferation	BP	109	6	0.0205292
GO:0031290	retinal ganglion cell axon guidance	BP	21	3	0.02062131
GO:0031098	stress-activated protein kinase signaling cascade	BP	243	10	0.02070867
GO:0032653	regulation of interleukin-10 production	BP	39	3	0.02106762
GO:0030159	receptor signaling complex scaffold activity	MF	24	3	0.02119211
GO:0051766	inositol trisphosphate kinase activity	MF	11	2	0.02169215
GO:0031224	intrinsic component of membrane	CC	4984	97	0.02178952
GO:0042325	regulation of phosphorylation	BP	1309	35	0.02185191
GO:0003994	aconitate hydratase activity	MF	2	1	0.02213544
GO:0031968	organelle outer membrane	CC	172	7	0.02217273
GO:0008794	arsenate reductase (glutaredoxin) activity	MF	1	1	0.02229749
GO:0030611	arsenate reductase activity	MF	1	1	0.02229749
GO:0004105	choline-phosphate cytidylyltransferase activity	MF	1	1	0.02235109
GO:0003708	retinoic acid receptor activity	MF	7	2	0.02242933
GO:0044710	single-organism metabolic process	BP	4012	84	0.02244772
GO:0015937	coenzyme A biosynthetic process	BP	10	2	0.022633
GO:0047894	flavonol 3-sulfotransferase activity	MF	3	1	0.02264024
GO:0001848	complement binding	MF	19	2	0.02285852
GO:0002690	positive regulation of leukocyte chemotaxis	BP	84	4	0.02291675
GO:0004595	pantetheine-phosphate adenylyltransferase activity	MF	1	1	0.0230987
GO:2000422	regulation of eosinophil chemotaxis	BP	2	1	0.02343711

GO:2000424	positive regulation of eosinophil chemotaxis	BP	2	1	0.02343711
GO:0007221	positive regulation of transcription of Notch receptor target	BP	7	2	0.02346651
GO:0030544	Hsp70 protein binding	MF	34	3	0.02411458
GO:0019867	outer membrane	CC	174	7	0.02412192
GO:0022610	biological adhesion	BP	1293	37	0.02420698
GO:0032481	positive regulation of type I interferon production	BP	68	4	0.02426584
GO:0032613	interleukin-10 production	BP	41	3	0.02440013
GO:0071629	ubiquitin-dependent catabolism of misfolded proteins by cytop	BP	1	1	0.02484995
GO:0002225	positive regulation of antimicrobial peptide production	BP	3	1	0.02486525
GO:0002760	positive regulation of antimicrobial humoral response	BP	3	1	0.02486525
GO:0002803	positive regulation of antibacterial peptide production	BP	3	1	0.02486525
GO:0022408	negative regulation of cell-cell adhesion	BP	132	6	0.02532673
GO:0002830	positive regulation of type 2 immune response	BP	15	2	0.02556685
GO:0035384	thioester biosynthetic process	BP	62	4	0.02562379
GO:0071616	acyl-CoA biosynthetic process	BP	62	4	0.02562379
GO:1904294	positive regulation of ERAD pathway	BP	11	2	0.02565471
GO:0016021	integral component of membrane	CC	4860	94	0.025748
GO:0060600	dichotomous subdivision of an epithelial terminal unit	BP	10	2	0.02622398
GO:0016337	single organismal cell-cell adhesion	BP	564	18	0.02623514
GO:0001569	branching involved in blood vessel morphogenesis	BP	30	3	0.02649247
GO:0043183	vascular endothelial growth factor receptor 1 binding	MF	2	1	0.026695
GO:0033116	endoplasmic reticulum-Golgi intermediate compartment memb	CC	65	4	0.02681441
GO:1903959	regulation of anion transmembrane transport	BP	32	3	0.02681468
GO:0006898	receptor-mediated endocytosis	BP	274	10	0.02707148
GO:0043209	myelin sheath	CC	160	7	0.02716024
GO:0001325	formation of extrachromosomal circular DNA	BP	13	2	0.0271968
GO:0090656	t-circle formation	BP	13	2	0.0271968
GO:0090737	telomere maintenance via telomere trimming	BP	13	2	0.0271968
GO:0007289	spermatid nucleus differentiation	BP	18	2	0.0271991
GO:0007586	digestion	BP	157	6	0.02747125

GO:0002215	defense response to nematode	BP	3	1	0.02761556
GO:0070528	protein kinase C signaling	BP	27	3	0.0279091
GO:0030890	positive regulation of B cell proliferation	BP	37	3	0.02817994
GO:0080135	regulation of cellular response to stress	BP	579	18	0.02825203
GO:0019220	regulation of phosphate metabolic process	BP	1536	39	0.02839562
GO:0034512	box C/D snoRNA binding	MF	1	1	0.02854108
GO:0030224	monocyte differentiation	BP	32	3	0.0285793
GO:1903131	mononuclear cell differentiation	BP	32	3	0.0285793
GO:0071288	cellular response to mercury ion	BP	3	1	0.02873929
GO:0051246	regulation of protein metabolic process	BP	2395	55	0.02894285
GO:0046651	lymphocyte proliferation	BP	229	8	0.02896684
GO:0051249	regulation of lymphocyte activation	BP	371	12	0.02918867
GO:0005590	collagen type VII trimer	CC	1	1	0.02942301
GO:0098648	collagen anchoring fibril	CC	1	1	0.02942301
GO:0098652	collagen type VII anchoring fibril	CC	1	1	0.02942301
GO:0006684	sphingomyelin metabolic process	BP	14	2	0.02955126
GO:1900273	positive regulation of long-term synaptic potentiation	BP	11	2	0.02978548
GO:0033674	positive regulation of kinase activity	BP	492	16	0.02978892
GO:0032943	mononuclear cell proliferation	BP	231	8	0.02994811
GO:0008308	voltage-gated anion channel activity	MF	16	2	0.03005583
GO:0002440	production of molecular mediator of immune response	BP	151	6	0.0301305
GO:0004747	ribokinase activity	MF	1	1	0.03026372
GO:0001971	negative regulation of activation of membrane attack complex	BP	1	1	0.03059651
GO:0005576	extracellular region	CC	4313	83	0.0307852
GO:0051538	3 iron, 4 sulfur cluster binding	MF	3	1	0.0310447
GO:0070633	transepithelial transport	BP	17	2	0.03114158
GO:0015936	coenzyme A metabolic process	BP	14	2	0.03186011
GO:0031296	B cell costimulation	BP	4	1	0.03214863
GO:0032954	regulation of cytokinetic process	BP	2	1	0.03228194
GO:1903436	regulation of mitotic cytokinetic process	BP	2	1	0.03228194

GO:1903438	positive regulation of mitotic cytokinetic process	BP	2	1	0.03228194
GO:1903490	positive regulation of mitotic cytokinesis	BP	2	1	0.03228194
GO:0014041	regulation of neuron maturation	BP	11	2	0.03229081
GO:0032479	regulation of type I interferon production	BP	112	5	0.03270893
GO:0002784	regulation of antimicrobial peptide production	BP	4	1	0.03286496
GO:0002786	regulation of antibacterial peptide production	BP	4	1	0.03286496
GO:0004461	lactose synthase activity	MF	3	1	0.03312986
GO:0009019	tRNA (guanine-N1-)-methyltransferase activity	MF	4	1	0.03326342
GO:0032606	type I interferon production	BP	113	5	0.03327028
GO:0016773	phosphotransferase activity, alcohol group as acceptor	MF	736	23	0.03344233
GO:0043560	insulin receptor substrate binding	MF	11	2	0.03344694
GO:0098602	single organism cell adhesion	BP	618	19	0.03345646
GO:0035691	macrophage migration inhibitory factor signaling pathway	BP	2	1	0.0336114
GO:0097363	protein O-GlcNAc transferase activity	MF	1	1	0.03366889
GO:1903317	regulation of protein maturation	BP	80	4	0.03380791
GO:0016020	membrane	CC	8385	158	0.03386797
GO:0038192	gastric inhibitory peptide signaling pathway	BP	1	1	0.03402948
GO:0016519	gastric inhibitory peptide receptor activity	MF	1	1	0.03402948
GO:0023014	signal transduction by protein phosphorylation	BP	859	24	0.03443104
GO:0030961	peptidyl-arginine hydroxylation	BP	3	1	0.03445546
GO:0005136	interleukin-4 receptor binding	MF	3	1	0.0345677
GO:0031399	regulation of protein modification process	BP	1572	39	0.03458946
GO:0001716	L-amino-acid oxidase activity	MF	1	1	0.0346953
GO:1905897	regulation of response to endoplasmic reticulum stress	BP	69	4	0.03479849
GO:0048101	calcium- and calmodulin-regulated 3',5'-cyclic-GMP phosphodie	MF	2	1	0.0348345
GO:0007155	cell adhesion	BP	1287	36	0.03490464
GO:0099617	matrix side of mitochondrial inner membrane	CC	1	1	0.03496003
GO:1903659	regulation of complement-dependent cytotoxicity	BP	3	1	0.03523857
GO:1903660	negative regulation of complement-dependent cytotoxicity	BP	3	1	0.03523857
GO:0002901	mature B cell apoptotic process	BP	4	1	0.03536807

GO:0002905	regulation of mature B cell apoptotic process	BP	4	1	0.03536807
GO:0002906	negative regulation of mature B cell apoptotic process	BP	4	1	0.03536807
GO:1904535	positive regulation of telomeric loop disassembly	BP	1	1	0.03553674
GO:0008037	cell recognition	BP	134	6	0.03561473
GO:0021649	vestibulocochlear nerve structural organization	BP	1	1	0.03576183
GO:0060301	positive regulation of cytokine activity	BP	1	1	0.03576183
GO:0061552	ganglion morphogenesis	BP	1	1	0.03576183
GO:1902378	VEGF-activated neuropilin signaling pathway involved in axon g	BP	1	1	0.03576183
GO:1904835	dorsal root ganglion morphogenesis	BP	1	1	0.03576183
GO:1905040	otic placode development	BP	1	1	0.03576183
GO:0030510	regulation of BMP signaling pathway	BP	78	5	0.0359235
GO:0034057	RNA strand-exchange activity	MF	2	1	0.03601968
GO:0002684	positive regulation of immune system process	BP	873	22	0.03611204
GO:0034236	protein kinase A catalytic subunit binding	MF	14	2	0.03614665
GO:0043564	Ku70:Ku80 complex	CC	3	1	0.0361605
GO:1900220	semaphorin-plexin signaling pathway involved in bone trabecul	BP	2	1	0.0362238
GO:0030513	positive regulation of BMP signaling pathway	BP	31	3	0.03632836
GO:0036010	protein localization to endosome	BP	12	2	0.03638169
GO:0017053	transcriptional repressor complex	CC	79	5	0.03640259
GO:0000165	MAPK cascade	BP	824	23	0.03640761
GO:0043549	regulation of kinase activity	BP	761	22	0.0366068
GO:0032736	positive regulation of interleukin-13 production	BP	14	2	0.03660832
GO:0048384	retinoic acid receptor signaling pathway	BP	30	3	0.03700308
GO:0022829	wide pore channel activity	MF	20	2	0.03710697
GO:0071475	cellular hyperosmotic salinity response	BP	3	1	0.03723109
GO:0001651	dense fibrillar component	CC	2	1	0.03731637
GO:0070053	thrombospondin receptor activity	MF	4	1	0.03749866
GO:0098552	side of membrane	CC	400	13	0.03773285
GO:0042098	T cell proliferation	BP	163	6	0.03777019
GO:0032838	cell projection cytoplasm	CC	58	4	0.03868492

GO:0006972	hyperosmotic response	BP	22	2	0.03887999
GO:0071624	positive regulation of granulocyte chemotaxis	BP	30	2	0.03970738
GO:0001165	RNA polymerase I upstream control element sequence-specific	MF	1	1	0.03973146
GO:0032433	filopodium tip	CC	12	2	0.03987409
GO:0034185	apolipoprotein binding	MF	16	2	0.0399286
GO:0090335	regulation of brown fat cell differentiation	BP	13	2	0.04006859
GO:0048569	post-embryonic animal organ development	BP	15	2	0.04008263
GO:0006102	isocitrate metabolic process	BP	4	1	0.04009709
GO:0070661	leukocyte proliferation	BP	244	8	0.04012927
GO:1902630	regulation of membrane hyperpolarization	BP	1	1	0.04016539
GO:1902631	negative regulation of membrane hyperpolarization	BP	1	1	0.04016539
GO:0016301	kinase activity	MF	797	24	0.04020675
GO:0050294	steroid sulfotransferase activity	MF	4	1	0.04023401
GO:0002775	antimicrobial peptide production	BP	5	1	0.04035631
GO:0002778	antibacterial peptide production	BP	5	1	0.04035631
GO:0061844	antimicrobial humoral immune response mediated by antimicro	BP	5	1	0.04035631
GO:0046601	positive regulation of centriole replication	BP	3	1	0.04036744
GO:0006066	alcohol metabolic process	BP	284	9	0.04048214
GO:0002694	regulation of leukocyte activation	BP	428	13	0.04056484
GO:0002759	regulation of antimicrobial humoral response	BP	5	1	0.04058526
GO:0003945	N-acetyllactosamine synthase activity	MF	4	1	0.04064112
GO:0050865	regulation of cell activation	BP	462	14	0.04065513
GO:0044459	plasma membrane part	CC	2402	57	0.04073024
GO:0030149	sphingolipid catabolic process	BP	20	2	0.04076557
GO:0071218	cellular response to misfolded protein	BP	18	2	0.04089485
GO:0031208	POZ domain binding	MF	2	1	0.04117734
GO:0043931	ossification involved in bone maturation	BP	17	2	0.04125438
GO:0033082	regulation of extrathymic T cell differentiation	BP	2	1	0.04137327
GO:0050868	negative regulation of T cell activation	BP	86	4	0.04140719
GO:0002688	regulation of leukocyte chemotaxis	BP	99	4	0.04145941

GO:0000470	maturation of LSU-rRNA	BP	22	2	0.04154052
GO:0001934	positive regulation of protein phosphorylation	BP	840	23	0.04163576
GO:0016358	dendrite development	BP	180	9	0.04164184
GO:0016581	NuRD complex	CC	16	2	0.04165915
GO:0090545	CHD-type complex	CC	16	2	0.04165915
GO:0019912	cyclin-dependent protein kinase activating kinase activity	MF	2	1	0.04171974
GO:0030658	transport vesicle membrane	CC	169	7	0.04173752
GO:0034383	low-density lipoprotein particle clearance	BP	18	2	0.0419197
GO:0033387	putrescine biosynthetic process from ornithine	BP	3	1	0.04199893
GO:0004998	transferrin receptor activity	MF	2	1	0.04217131
GO:0098656	anion transmembrane transport	BP	252	9	0.04268454
GO:0002325	natural killer cell differentiation involved in immune response	BP	4	1	0.0427707
GO:0032826	regulation of natural killer cell differentiation involved in immu	BP	4	1	0.0427707
GO:0047325	inositol tetrakisphosphate 1-kinase activity	MF	1	1	0.04311568
GO:0052725	inositol-1,3,4-trisphosphate 6-kinase activity	MF	1	1	0.04311568
GO:0052726	inositol-1,3,4-trisphosphate 5-kinase activity	MF	1	1	0.04311568
GO:0052825	inositol-1,3,4,5,6-pentakisphosphate 1-phosphatase activity	MF	1	1	0.04311568
GO:0052830	inositol-1,3,4,6-tetrakisphosphate 6-phosphatase activity	MF	1	1	0.04311568
GO:0052831	inositol-1,3,4,6-tetrakisphosphate 1-phosphatase activity	MF	1	1	0.04311568
GO:0052835	inositol-3,4,6-trisphosphate 1-kinase activity	MF	1	1	0.04311568
GO:0030449	regulation of complement activation	BP	30	2	0.0432557
GO:0035645	enteric smooth muscle cell differentiation	BP	1	1	0.04325891
GO:0015106	bicarbonate transmembrane transporter activity	MF	19	2	0.04328583
GO:0017154	semaphorin receptor activity	MF	9	2	0.04331763
GO:0046328	regulation of JNK cascade	BP	157	7	0.04338081
GO:0019955	cytokine binding	MF	90	4	0.04343401
GO:0030011	maintenance of cell polarity	BP	15	2	0.04346225
GO:0061441	renal artery morphogenesis	BP	2	1	0.04347365
GO:0098971	anterograde dendritic transport of neurotransmitter receptor c	BP	2	1	0.04355572
GO:0033866	nucleoside bisphosphate biosynthetic process	BP	16	2	0.04359862

GO:0034030	ribonucleoside bisphosphate biosynthetic process	BP	16	2	0.04359862
GO:0034033	purine nucleoside bisphosphate biosynthetic process	BP	16	2	0.04359862
GO:0032793	positive regulation of CREB transcription factor activity	BP	14	2	0.04368727
GO:0008093	cytoskeletal adaptor activity	MF	11	2	0.04388339
GO:0034237	protein kinase A regulatory subunit binding	MF	16	2	0.0439479
GO:0003858	3-hydroxybutyrate dehydrogenase activity	MF	2	1	0.04398158
GO:1904799	regulation of neuron remodeling	BP	1	1	0.04401908
GO:1904800	negative regulation of neuron remodeling	BP	1	1	0.04401908
GO:2000173	negative regulation of branching morphogenesis of a nerve	BP	1	1	0.04401908
GO:0042104	positive regulation of activated T cell proliferation	BP	26	2	0.04412493
GO:0008157	protein phosphatase 1 binding	MF	18	2	0.04440681
GO:0022850	serotonin-gated cation-selective channel activity	MF	5	1	0.04465668
GO:0060977	coronary vasculature morphogenesis	BP	13	2	0.04469619
GO:0008194	UDP-glycosyltransferase activity	MF	134	6	0.04475758
GO:0043410	positive regulation of MAPK cascade	BP	451	14	0.04476854
GO:0001819	positive regulation of cytokine production	BP	375	11	0.0449033
GO:0036114	medium-chain fatty-acyl-CoA catabolic process	BP	1	1	0.04496298
GO:0036115	fatty-acyl-CoA catabolic process	BP	1	1	0.04496298
GO:0036116	long-chain fatty-acyl-CoA catabolic process	BP	1	1	0.04496298
GO:1900533	palmitic acid metabolic process	BP	1	1	0.04496298
GO:1900535	palmitic acid biosynthetic process	BP	1	1	0.04496298
GO:0035692	macrophage migration inhibitory factor receptor complex	CC	2	1	0.04498089
GO:0033592	RNA strand annealing activity	MF	3	1	0.04499421
GO:0031673	H zone	CC	1	1	0.04502484
GO:0042102	positive regulation of T cell proliferation	BP	90	4	0.04503646
GO:0007218	neuropeptide signaling pathway	BP	95	4	0.04512517
GO:0046726	positive regulation by virus of viral protein levels in host cell	BP	4	1	0.04521635
GO:0031672	A band	CC	40	3	0.04524608
GO:0008349	MAP kinase kinase kinase kinase activity	MF	4	1	0.04524997
GO:0018105	peptidyl-serine phosphorylation	BP	253	10	0.04533935

GO:0050858	negative regulation of antigen receptor-mediated signaling patl	BP	21	2	0.04534404
GO:0097374	sensory neuron axon guidance	BP	2	1	0.04552331
GO:0035964	COPI-coated vesicle budding	BP	4	1	0.04566802
GO:0048200	Golgi transport vesicle coating	BP	4	1	0.04566802
GO:0048205	COPI coating of Golgi vesicle	BP	4	1	0.04566802
GO:0006796	phosphate-containing compound metabolic process	BP	2934	65	0.04571555
GO:0002438	acute inflammatory response to antigenic stimulus	BP	20	2	0.04580707
GO:0060218	hematopoietic stem cell differentiation	BP	17	2	0.0458997
GO:0043405	regulation of MAP kinase activity	BP	311	11	0.04596409
GO:0032874	positive regulation of stress-activated MAPK cascade	BP	132	6	0.04626419
GO:0099568	cytoplasmic region	CC	279	11	0.0466814
GO:0006955	immune response	BP	1753	36	0.04670705
GO:1902412	regulation of mitotic cytokinesis	BP	3	1	0.04684614
GO:0033326	cerebrospinal fluid secretion	BP	3	1	0.04709229
GO:0051247	positive regulation of protein metabolic process	BP	1439	35	0.04732366
GO:0005161	platelet-derived growth factor receptor binding	MF	14	2	0.04733418
GO:0035338	long-chain fatty-acyl-CoA biosynthetic process	BP	42	3	0.04733488
GO:0045178	basal part of cell	CC	47	3	0.04734001
GO:0048010	vascular endothelial growth factor receptor signaling pathway	BP	89	5	0.04749544
GO:0032733	positive regulation of interleukin-10 production	BP	27	2	0.0477101
GO:0070542	response to fatty acid	BP	76	4	0.04772529
GO:0098589	membrane region	CC	347	12	0.04774569
GO:0051347	positive regulation of transferase activity	BP	626	18	0.04779274
GO:1990630	IRE1-RACK1-PP2A complex	CC	2	1	0.04812798
GO:0016772	transferase activity, transferring phosphorus-containing groups	MF	933	26	0.04826464
GO:1903038	negative regulation of leukocyte cell-cell adhesion	BP	93	4	0.04870941
GO:0070304	positive regulation of stress-activated protein kinase signaling c	BP	133	6	0.04874784
GO:0032268	regulation of cellular protein metabolic process	BP	2224	50	0.04875983
GO:0045064	T-helper 2 cell differentiation	BP	16	2	0.04896121
GO:1902359	Notch signaling pathway involved in somitogenesis	BP	2	1	0.04908528

GO:1902366	regulation of Notch signaling pathway involved in somitogenesi	BP	2	1	0.04908528
GO:1902367	negative regulation of Notch signaling pathway involved in som	BP	2	1	0.04908528
GO:0018205	peptidyl-lysine modification	BP	341	11	0.0491019
GO:0072697	protein localization to cell cortex	BP	2	1	0.0491193
GO:0060994	regulation of transcription from RNA polymerase II promoter in	BP	2	1	0.04913173
GO:0097232	lamellar body membrane	CC	2	1	0.04915651
GO:0097233	alveolar lamellar body membrane	CC	2	1	0.04915651
GO:0046949	fatty-acyl-CoA biosynthetic process	BP	43	3	0.04941378
GO:1903860	negative regulation of dendrite extension	BP	2	1	0.04973755
GO:0019538	protein metabolic process	BP	5123	102	0.04983053
GO:0050778	positive regulation of immune response	BP	621	16	0.04986302
GO:1990829	C-rich single-stranded DNA binding	MF	2	1	0.04988326
GO:0031528	microvillus membrane	CC	21	2	0.04997832
